# Supplementary material for: Comparative Proteomic Analysis Reveals Mx1 Inhibits Senecavirus A Replication in PK-15 Cells by Interacting with the Capsid Proteins VP1, VP2 and VP3
Source: Viruses. 2022 Apr 21;14(5):863. doi: 10.3390/v14050863 (PMC9147370; doi:10.3390/v14050863)
Supplement: Supplementary file 1 [file viruses-14-00863-s001.zip › Supplementary Table S1.pdf]

**Supplementary Table S1 The differentially expressed proteins identified by TMT labeling coupled with LC-MS/MS analysis of PK-15 cells in response to SVA infection at 12 hpi**

| Protein name | Uniport accession No. | $Log_2$ ratios (infection/control) | peptides | Sequence coverage (%) | P-values | functions |
|---|---|---|---|---|---|---|
| **Upregulated proteins in PK-15-infected cells (a total of 314 proteins)** | | | | | | |
| Genome polyprotein | A0A5C2GZI4 | 2.392431 | 1 | 39 | 0.000729 | Function unknown |
| Interferon-induced GTP-binding protein Mx1 | A0A4X1TKB5 | 1.536855 | 6 | 78 | 0.00925 | Immune effector process; negative regulation of viral process |
| Inflammatory response protein 6 | A0A287B6T9 | 1.486117 | 14 | 44 | 0.007366 | T cell differentiation; negative regulation of viral process |
| Genome polyprotein | A0A649YC94 | 1.469757 | 1 | 30 | 0.004355 | Function unknown |
| Interferon-induced GTP-binding protein Mx2 | A0A480HPX5 | 1.421313 | 26 | 54 | 0.00044 | Small molecule binding, establishment of localization in cell |
| 2'-5' oligoadenylate synthase | Q56VQ1 | 1.247479 | 30 | 54 | 0.002377 | Ribonucleotide binding |
| Uncharacterized protein | A0A5G2RJF6 | 1.218727 | 1 | 1 | 0.000446 | Function unknown |
| Tumor-associated calcium signal transducer 2 | F1S7A4 | 1.200562 | 8 | 34 | 0.006537 | Regulation of stem cell differentiation; positive regulation of stem cell differentiation |
| Interferon-stimulated protein 60 | F1SCY2 | 1.114459 | 14 | 41 | 0.002878 | Protein binding, immune effector process |
| C-C motif chemokine (Fragment) | A1YM33 | 1.110968 | 3 | 47 | 0.01546 | Catalytic activity, positive regulation of protein phosphorylation |
| Interferon-stimulated protein 56 | J7FIC7 | 1.107499 | 30 | 64 | 0.002542 | Regulation of defense response to virus, regulation of response to exteRNAl stimulus |

| | | | | | | |
|---|---|---|---|---|---|---|
| Interferon-induced protein with tetratricopeptide repeats 2 | K9J6I9 | 1.069138 | 18 | 34 | 0.003941 | Cellular response to stimulus, response to cytokine |
| DNA topoisomerase I | I3LTP0 | 1.052397 | 1 | 2 | 0.03377 | Function unknown |
| mRNA-capping enzyme | A0A4X1VEE3 | 1.01955 | 3 | 7 | 0.02165 | Function unknown |
| Genome polyprotein | A0A6B9QIJ0 | 1.014207 | 1 | 30 | 0.000258 | Function unknown |
| RNA helicase | A8CYB8 | 1.000579 | 31 | 41 | 0.002151 | Helicase activity, regulation of locomotion |
| Uncharacterized protein | A0A287A9J8 | 0.985966 | 1 | 8 | 0.000852 | Function unknown |
| TLDc domain-containing protein | I3LMG2 | 0.985906 | 1 | 45 | 0.00663 | Function unknown |
| G domain-containing protein | F1S9T2 | 0.954928 | 1 | 39 | 0.001392 | GTP binding |
| Arf-GAP with Rho-GAP domain, ANK repeat and PH domain-containing protein 1 isoform c | A0A481BP67 | 0.95051 | 1 | 10 | 0.01857 | Function unknown |
| Protein PML isoform 2 | A0A480JXC7 | 0.946937 | 2 | 50 | 0.02126 | Function unknown |
| FIP-RBD domain-containing protein | A0A4X1UDM9 | 0.937329 | 1 | 32 | 0.02466 | Function unknown |
| Receptor-transporting protein 4 | A0A287B9T3 | 0.934535 | 5 | 12 | 0.006303 | Function unknown |
| Ubiquitin-like modifier | A0A287AB58 | 0.930694 | 6 | 50 | 0.002502 | Integrin binding; intracellular organelle |
| IRF tryptophan pentad repeat domain-containing protein | A0A5G2RH09 | 0.930143 | 6 | 20 | 0.004793 | Transcription regulator activity |
| GFO_IDH_MocA domain-containing protein | A0A4X1UEI6 | 0.922326 | 1 | 2 | 0.02394 | Oxidoreductase activity |
| PH domain-containing protein | A0A287BS42 | 0.902609 | 1 | 1 | 0.04178 | Integrin-mediated signaling pathway, cellular process; regulation of cellular process |
| Urokinase-type plasminogen | A0A286ZXK2 | 0.902583 | 9 | 24 | 0.00041 | Function unknown |

| | | | | | | |
|---|---|---|---|---|---|---|
| activator | | | | | | |
| Protein-serine/threonine phosphatase | A0A4X1U1Y2 | 0.901906 | 1 | 1 | 0.04074 | Function unknown |
| Uncharacterized protein | A0A287B4N8 | 0.884001 | 5 | 40 | 0.008604 | Function unknown |
| Transmembrane protein 147 | A0A4X1TJ50 | 0.871487 | 2 | 13 | 0.005086 | Macromolecular complex, organelle membrane, integral component of membrane |
| Noelin-2 isoform 2 | A0A480XA75 | 0.862249 | 1 | 2 | 0.03464 | Extrinsic component of plasma membrane; protein localization |
| Uncharacterized protein | A0A287B916 | 0.861888 | 1 | 2 | 0.03854 | Heterocyclic compound binding |
| 2',3'-cyclic-nucleotide 3'-phosphodiesterase | A0A287A9L8 | 0.86153 | 26 | 59 | 0.004682 | Binding |
| Uncharacterized protein | A0A4X1SEB3 | 0.858628 | 13 | 24 | 0.002072 | Function unknown |
| ANK_REP_REGION domain-containing protein | A0A4X1U612 | 0.852998 | 1 | 14 | 0.01982 | Function unknown |
| Kelch-like protein 36 | A0A480YWF8 | 0.852718 | 1 | 2 | 2.00E-05 | Function unknown |
| Uncharacterized protein | F1RM98 | 0.838745 | 6 | 19 | 0.000753 | Protein binding; positive regulation of cellular process |
| 2'-5' oligoadenylate synthase | Q5MX78 | 0.836352 | 2 | 27 | 0.004357 | Ribonucleotide binding |
| Uncharacterized protein | A0A4X1WD16 | 0.834263 | 1 | 6 | 0.03558 | Function unknown |
| DNA_MISMATCH_REPAIR_2 domain-containing protein | I3L5Z8 | 0.824216 | 2 | 2 | 0.01612 | Function unknown |
| TLDc domain-containing protein | A0A4X1UQ31 | 0.818645 | 7 | 43 | 0.006877 | Function unknown |
| Galectin-3-binding protein | A0A5G2RLP2 | 0.814593 | 13 | 34 | 0.005314 | Function unknown |
| Uncharacterized protein | F1SS06 | 0.810289 | 1 | 1 | 0.03231 | Function unknown |
| Galectin | A0A287A342 | 0.798613 | 11 | 45 | 0.01226 | Function unknown |
| Caspase 4, apoptosis-related cysteine peptidase | A0A0B8RT56 | 0.797204 | 9 | 28 | 0.000606 | Function unknown |
| FAM184 domain-containing | I3LV88 | 0.796314 | 1 | 1 | 0.03617 | Function unknown |

| | | | | | | |
|---|---|---|---|---|---|---|
| protein | | | | | | |
| CCHC-type domain-containing protein | F1SMW0 | 0.793917 | 2 | 3 | 0.01277 | Function unknown |
| Nuclear body protein SP140 isoform 5 | A0A480PYU5 | 0.779843 | 5 | 26 | 0.01075 | Function unknown |
| Apolipoprotein H | A0A140TAK8 | 0.767339 | 3 | 13 | 0.008719 | Function unknown |
| GB1/RHD3-type G domain-containing protein | A0A4X1U8M2 | 0.765946 | 1 | 6 | 0.02652 | Function unknown |
| Poly [ADP-ribose] polymerase | A0A4X1TD13 | 0.765145 | 45 | 31 | 0.00127 | Function unknown |
| RNA helicase | A0A287AKR1 | 0.76304 | 20 | 21 | 0.001596 | Binding |
| RECA_2 domain-containing protein | A0A4X1TR78 | 0.761278 | 1 | 3 | 3.40E-05 | Anion binding; DNA metabolic process |
| Fibroblast growth factor | A0A287BGK8 | 0.760152 | 1 | 3 | 0.02677 | Function unknown |
| Nuclear autoantigen Sp-100 isoform 4 | R4L6V8 | 0.75475 | 2 | 28 | 0.03474 | Intracellular membrane-bounded organelle; nucleus |
| Uncharacterized protein | A0A4X1URS1 | 0.749866 | 1 | 42 | 0.02308 | Function unknown |
| Transporter 2, ATP-binding cassette, sub-family B (MDR/TAP) | A5D9J7 | 0.747746 | 16 | 31 | 0.01017 | Ribonucleotide binding |
| GB1/RHD3-type G domain-containing protein | A0A287BSF6 | 0.745783 | 2 | 30 | 0.02444 | Ribonucleotide binding |
| Interferon regulatory factor 7 | X2KPB3 | 0.743615 | 2 | 5 | 0.002987 | Transcription factor activity; sequence-specific DNA binding; adaptive immune response |
| Tumor necrosis factor receptor superfamily member 5 | Q8SQ34 | 0.739224 | 6 | 30 | 0.03582 | Signaling receptor activity; positive regulation of cellular biosynthetic process |
| Pleckstrin homology-like domain family A member 1 | F1SGD8 | 0.736577 | 1 | 2 | 0.00788 | Function unknown |

| | | | | | | |
|---|---|---|---|---|---|---|
| CCDC66 domain-containing protein | I3L7C7 | 0.73314 | 1 | 1 | 0.02096 | Function unknown |
| Uncharacterized protein | A0A5G2QLT7 | 0.731775 | 2 | 4 | 0.02382 | Function unknown |
| Aminopeptidase | K9J4P1 | 0.730795 | 7 | 7 | 0.03046 | Function unknown |
| RNA helicase | F1S0P6 | 0.729356 | 12 | 22 | 0.004083 | Anion binding; negative regulation of cellular process |
| Interferon regulatory factor | A0A5S6GDS9 | 0.722825 | 5 | 24 | 0.000597 | Transcription factor activity; sequence-specific DNA binding, negative regulation of macromolecule biosynthetic process |
| Homeobox protein cut-like | A0A4X1U3F5 | 0.713423 | 2 | 2 | 0.01952 | Function unknown |
| Epithelial-stromal interaction protein 1 isoform 2 | A0A286ZSG6 | 0.711802 | 9 | 34 | 0.007058 | Function unknown |
| UBC core domain-containing protein | A0A5G2Q9I0 | 0.710535 | 6 | 79 | 0.005605 | Ribonucleotide binding |
| Reticulon | A0A4X1VKW2 | 0.709841 | 1 | 11 | 0.01387 | Function unknown |
| 2'-5'-oligoadenylate synthase-like protein isoform a | F1RIV0 | 0.697316 | 13 | 44 | 0.03516 | Ribonucleotide binding |
| GTPase-activating Rap/Ran-GAP domain-like protein 3 isoform X1 | I3LIY1 | 0.691048 | 1 | 1 | 0.03553 | Function unknown |
| Electron transfer flavoprotein-ubiquinone oxidoreductase | F1RW89 | 0.686931 | 1 | 46 | 0.002947 | Oxidoreductase activity |
| Interferon-induced GTP-binding protein Mx1 | A0A481BA72 | 0.686812 | 1 | 77 | 0.01551 | Catalytic activity; cellular metabolic process |
| Armadillo repeat-containing protein 4 isoform X3 | A0A480MFY5 | 0.68409 | 2 | 2 | 0.04023 | Function unknown |
| Nucleotide-binding oligomerization domain 1 | B0FSM7 | 0.679421 | 1 | 1 | 0.02509 | Ribonucleotide binding |

| | | | | | | |
|---|---|---|---|---|---|---|
| 2'-5'-oligoadenylate synthase 1 | Q29599 | 0.677282 | 3 | 45 | 0.006649 | 2'-5'-oliprimary metabolic process; cellular metabolic process |
| Fibroblast growth factor-inducible 14 | B7SLY2 | 0.676496 | 2 | 23 | 0.01422 | Positive regulation of developmental growth; regulation of developmental growth |
| PH domain-containing protein | A0A4X1VUK6 | 0.673899 | 8 | 13 | 0.00743 | Function unknown |
| Exonuclease domain-containing protein | A0A4X1W796 | 0.666049 | 7 | 46 | 0.002538 | Function unknown |
| LRRC37AB_C domain-containing protein | A0A286ZIV7 | 0.665644 | 2 | 2 | 0.008392 | Function unknown |
| Phospholipid-transporting ATPase | A0A4X1ULA4 | 0.661048 | 3 | 3 | 0.03414 | Transmembrane transporter activity; leukocyte degranulation |
| Uncharacterized protein | I3LB77 | 0.66024 | 5 | 17 | 0.000693 | MRNA 3'-utr binding, organic substance transport |
| IFITM2 | F4ZS15 | 0.657588 | 1 | 28 | 0.00304 | Integral component of membrane; cellular anatomical entity |
| Uncharacterized protein | A0A4X1VHT0 | 0.651654 | 21 | 33 | 0.001172 | Transferase activity, transferring pentosyl groups; regulation of mRNA metabolic process |
| Uncharacterized protein | I3L5Z9 | 0.648297 | 60 | 42 | 0.002487 | Function unknown |
| Polypyrimidine tract-binding protein 1 | A0A286ZPA4 | 0.647217 | 1 | 61 | 0.003047 | Function unknown |
| Uncharacterized protein | A0A4X1V406 | 0.643524 | 20 | 37 | 0.004326 | Ribonucleotide binding |
| Dynamin GTPase | A0A4X1VG57 | 0.629307 | 1 | 41 | 0.004953 | Function unknown |
| Non-specific serine/threonine protein kinase | F1RQR3 | 0.626525 | 3 | 3 | 0.000665 | Function unknown |
| Coagulation factor III (Fragment) | A0A481D288 | 0.624166 | 3 | 15 | 0.001827 | Lipid binding, positive regulation of biological process; protein processing |
| Putative E3 ubiquitin-protein ligase HERC6 isoform 1 | A0A480M1Q1 | 0.616749 | 1 | 33 | 0.02974 | Function unknown |
| Uncharacterized protein | A0A287BKF1 | 0.614608 | 24 | 42 | 0.006977 | Ribonucleotide binding |

| | | | | | | |
|---|---|---|---|---|---|---|
| Uncharacterized protein | A0A5G2RNJ9 | 0.614085 | 1 | 2 | 0.004226 | Function unknown |
| Ribonuclease H | A0A5G2R922 | 0.612383 | 1 | 7 | 0.01585 | Function unknown |
| Uncharacterized protein | F1SL31 | 0.611186 | 1 | 34 | 0.015 | Function unknown |
| Zinc finger protein 555 isoform 2 | A0A287A1D7 | 0.609357 | 1 | 6 | 0.04632 | Function unknown |
| Plasminogen activator inhibitor 1 | P79335 | 0.606957 | 12 | 40 | 0.000552 | Serine-type endopeptidase inhibitor activity; negative regulation of endopeptidase activity |
| Myeloid cell nuclear differentiation antigen | A0A287AP08 | 0.603852 | 4 | 10 | 0.004269 | Function unknown |
| Inhibitor_Mig-6 domain-containing protein | A0A287A7S3 | 0.603145 | 2 | 4 | 0.003714 | Function unknown |
| Collagen IV NC1 domain-containing protein | A0A4X1U499 | 0.602292 | 1 | 1 | 0.002095 | Function unknown |
| E3 ubiquitin-protein ligase RNF213 isoform 3 | A0A480YJW7 | 0.600502 | 27 | 34 | 0.005266 | Function unknown |
| Uncharacterized protein | A0A4X1W154 | 0.596663 | 2 | 0 | 0.01719 | Function unknown |
| RING-type domain-containing protein | A0A4X1UID3 | 0.596581 | 2 | 30 | 0.02149 | Function unknown |
| J domain-containing protein | A0A5G2R4I8 | 0.591194 | 1 | 42 | 0.03637 | Function unknown |
| Arylamine N-acetyltransferase | A0A480RQR3 | 0.589917 | 1 | 41 | 0.008489 | Function unknown |
| Syndecan | A0A5S6HZW1 | 0.587846 | 8 | 24 | 0.01365 | Locomotion, cell motility |
| Poly ADP-ribose polymerase 12 | A0A480N6U6 | 0.580134 | 3 | 8 | 0.02232 | Transferase activity; transferring glycosyl groups |
| Uncharacterized protein | A0A287B544 | 0.576938 | 2 | 38 | 0.006583 | Function unknown |
| Alpha-actinin-4 isoform 1 | A0A480ZDQ8 | 0.575463 | 1 | 72 | 0.002139 | Protein binding, protein transport; signal transduction |
| Procollagen-proline 4- | A1X898 | 0.575381 | 1 | 59 | 0.04202 | Binding |

| | | | | | | |
|---|---|---|---|---|---|---|
| dioxygenase | | | | | | |
| Phospholipid-transporting ATPase | M3TYF0 | 0.573945 | 1 | 1 | 0.0323 | Function unknown |
| Uncharacterized protein | A0A287ARP2 | 0.573711 | 1 | 17 | 0.001977 | Function unknown |
| Uridine-cytidine kinase | A0A4X1T9P1 | 0.571887 | 2 | 13 | 0.03183 | Small molecule binding; biosynthetic process |
| HECT domain-containing protein | A0A4X1U9D8 | 0.570222 | 25 | 33 | 0.01593 | Function unknown |
| Uncharacterized protein | F1S5B5 | 0.562586 | 2 | 5 | 0.004215 | Function unknown |
| BLOC-1-related complex subunit 7 | A0A4X1TT04 | 0.561276 | 1 | 4 | 0.02158 | Function unknown |
| F-BAR domain-containing protein | A0A5G2RMN3 | 0.560004 | 1 | 1 | 0.003662 | Function unknown |
| Tripartite motif-containing protein 26 | B6ICV1 | 0.559532 | 5 | 11 | 0.04198 | Binding |
| Thiopurine S-methyltransferase | F1RUH7 | 0.559354 | 1 | 68 | 0.001714 | Methyltransferase activity; methylation |
| Voltage-dependent anion-selective channel protein 2 | A0A4X1UZQ9 | 0.559175 | 1 | 15 | 0.01559 | Function unknown |
| Uncharacterized protein | A0A4X1UW90 | 0.558469 | 27 | 18 | 0.02264 | Function unknown |
| Cationic amino acid transporter 2 | A0A4X1TZW6 | 0.55 | 2 | 5 | 0.003146 | Function unknown |
| Proline dehydrogenase | M3V806 | 0.548795 | 8 | 18 | 0.004382 | Function unknown |
| Transmembrane gamma-carboxyglutamic acid protein 1 isoform 1 | I3LMT5 | 0.548264 | 4 | 19 | 0.009384 | Calcium ion binding |
| Uncharacterized protein | A0A286ZTV2 | 0.547488 | 1 | 5 | 0.003487 | Integral component of membrane; cellular anatomical entity |

| | | | | | | |
|---|---|---|---|---|---|---|
| UPAR/Ly6 domain-containing protein | A0A287B805 | 0.541485 | 3 | 45 | 0.02681 | Function unknown |
| Zinc fingers and homeoboxes protein 1 | F1RR15 | 0.540952 | 3 | 6 | 0.01713 | Protein heterodimerization activity, negative regulation of macromolecule biosynthetic process |
| ATP-sulfurylase | A0A481BE86 | 0.537697 | 1 | 4 | 0.03342 | Function unknown |
| Ubiquitin carboxyl-terminal hydrolase | F1RTR5 | 0.533451 | 1 | 1 | 0.01852 | Chromatin binding; covalent chromatin modification |
| Urokinase plasminogen activator | A0A0B8RSD8 | 0.528392 | 4 | 16 | 0.000802 | Function unknown |
| Multi-drug resistance protein 1 transcript variant 2 | A0A0E3MY68 | 0.52502 | 1 | 36 | 0.004046 | Ribonucleotide binding |
| Polymeric immunoglobulin receptor | A0A4X1UQL1 | 0.514037 | 3 | 6 | 0.005094 | Integral component of membrane; cellular anatomical entity |
| Ig-like domain-containing protein | A0A287A1Y1 | 0.510542 | 6 | 23 | 0.0171 | Antigen processing and presentation of peptide antigen via mhc class I; antigen processing and presentation |
| Z-DNA-binding protein 1 | A0A481BW17 | 0.507553 | 2 | 39 | 2.10E-05 | Function unknown |
| Class E basic helix-loop-helix protein 41 | A0A287AYR6 | 0.505189 | 2 | 4 | 0.000608 | Function unknown |
| Endo/exonuclease/phosphatase domain-containing protein | A0A4X1V6E3 | 0.504998 | 1 | 2 | 0.006941 | Function unknown |
| Protein MCM10 homolog | A0A287AY37 | 0.502307 | 3 | 3 | 0.02363 | Function unknown |
| Plasminogen | P06867 | 0.499599 | 10 | 15 | 0.01564 | Regulation of blood coagulation; negative regulation of blood coagulation |
| RING-type E3 ubiquitin transferase | I3LRN0 | 0.498269 | 31 | 46 | 0.006589 | Function unknown |
| PARP-1 binding protein | A0A5G2QYX1 | 0.494283 | 1 | 1 | 0.01248 | Function unknown |
| Elongation factor 1-gamma | A0A4X1VKK0 | 0.493786 | 1 | 60 | 0.000305 | Function unknown |

| | | | | | | |
|---|---|---|---|---|---|---|
| VLIG-type G domain-containing protein | F1RMQ6 | 0.493208 | 9 | 4 | 0.006861 | Function unknown |
| Transcription factor jun-B | I3LJU2 | 0.490426 | 5 | 27 | 0.03037 | Transcription factor activity, sequence-specific DNA binding; anatomical structure development |
| Tether containing UBX domain for GLUT4 isoform 2 | A0A480NU10 | 0.485817 | 3 | 34 | 0.02326 | Function unknown |
| Receptor protein-tyrosine kinase | F1SUT8 | 0.482384 | 23 | 37 | 0.0006 | Transmembrane receptor protein kinase activity; negative regulation of signaling |
| Phosphoglycerate mutase | B5KJG2 | 0.481403 | 1 | 12 | 0.04568 | Isomerase activity, organic acid metabolic process |
| Rhomboid-related protein 4 isoform X1 | A0A480YX23 | 0.479941 | 1 | 6 | 0.01091 | Function unknown |
| Reticulon (Fragment) | A0A1Y1FJ11 | 0.479415 | 1 | 42 | 0.006662 | Cadherin binding; positive regulation of locomotion |
| ML domain-containing protein | A0A4X1TT31 | 0.476714 | 6 | 34 | 0.000972 | Function unknown |
| Uncharacterized protein | A0A287ABC3 | 0.476675 | 2 | 5 | 0.005583 | Function unknown |
| Uncharacterized protein | A0A4X1T995 | 0.475462 | 1 | 0 | 0.04474 | Cadherin binding, regulation of cytosolic calcium ion concentration |
| XIAP-associated factor 1 | K9IVW8 | 0.473008 | 6 | 29 | 0.01441 | Function unknown |
| ASD2 domain-containing protein | A0A4X1TVX4 | 0.472839 | 1 | 13 | 0.02071 | Function unknown |
| NPC intracellular cholesterol transporter 1 | A0A5G2RDJ5 | 0.471449 | 1 | 11 | 0.04671 | Small molecule binding, integral component of plasma membrane; establishment of localization in cell |
| Peptidase A1 domain-containing protein | A0A4X1T5P4 | 0.465517 | 1 | 3 | 0.0391 | Function unknown |
| Uncharacterized protein | A0A4X1SW44 | 0.459789 | 3 | 2 | 0.000608 | Protein heterodimerization activity; signal transduction |
| MHC class I antigen (Fragment) | K9LRJ6 | 0.459112 | 3 | 42 | 0.01817 | Antigen processing and presentation; integral component of membrane |
| Aprotinin (Fragment) | B0LXF7 | 0.454334 | 1 | 26 | 0.01914 | Enzyme regulator activity |

| | | | | | | |
|---|---|---|---|---|---|---|
| Tetratricopeptide repeat protein 14 isoform a | A0A287AVL3 | 0.452973 | 1 | 1 | 0.008206 | Binding |
| Uncharacterized protein | A0A4X1U686 | 0.451804 | 1 | 1 | 0.006141 | Function unknown |
| Beta-2-microglobulin | A0A4X1VG41 | 0.451493 | 3 | 32 | 0.03628 | Protein binding; positive regulation of cellular senescence |
| Uncharacterized protein | A0A4X1SXM2 | 0.449796 | 1 | 8 | 0.03855 | Receptor binding; signal transduction |
| TPR_REGION domain-containing protein | A0A4X1U2X7 | 0.446353 | 3 | 9 | 0.0193 | Function unknown |
| Uncharacterized protein | A0A287BD75 | 0.442298 | 1 | 1 | 0.001998 | Function unknown |
| Meiosis-specific nuclear structural protein 1 | F1RZG8 | 0.442128 | 3 | 4 | 0.002777 | Protein binding; positive regulation of biological process |
| Uncharacterized protein | A0A287A1X3 | 0.440465 | 11 | 21 | 0.004206 | Metal ion binding; membrane-bounded organelle |
| KIND domain-containing protein | A0A4X1VVD6 | 0.440438 | 4 | 5 | 0.001202 | Function unknown |
| DALR anticodon-binding domain-containing protein 3 isoform 1 | F1SKI7 | 0.438751 | 1 | 2 | 0.01795 | Function unknown |
| Uncharacterized protein | A0A5G2Q6W7 | 0.436452 | 6 | 11 | 0.0218 | Cell-cell adhesion; integral component of membrane |
| CST complex subunit CTC1 | A0A4X1V8I6 | 0.433298 | 3 | 4 | 0.0332 | Function unknown |
| Protein fem-1 homolog B | I3L6T4 | 0.429318 | 5 | 10 | 0.006546 | Receptor binding, morphogenesis of a branching structure; morphogenesis of an epithelium |
| Aquaporin-5 | A0A4X1WCJ0 | 0.425045 | 1 | 2 | 0.003184 | Transmembrane transporter activity |
| ADP-ribosyl cyclase/cyclic ADP-ribose hydrolase | A0A481D5X2 | 0.424914 | 1 | 32 | 0.002487 | Signaling adaptor activity; phosphate-containing compound metabolic process |
| Signal transducer and activator of transcription | A0A4X1THM1 | 0.422745 | 1 | 60 | 0.04674 | Transcription factor activity; sequence-specific DNA binding; regulation of cell differentiation |
| Uncharacterized protein | F1S6F1 | 0.42059 | 2 | 6 | 0.003557 | Function unknown |

| | | | | | | |
|---|---|---|---|---|---|---|
| Ribosomal RNA processing protein 36 homolog isoform 1 | A0A480TNH7 | 0.417719 | 5 | 24 | 0.0084 | Nucleic acid metabolic process; nucleic acid phosphodiester bond hydrolysis |
| Copine-2 | A0A287AKE4 | 0.417499 | 2 | 6 | 0.032 | Function unknown |
| Uncharacterized protein | A0A5G2R0A9 | 0.416925 | 23 | 55 | 0.00731 | Function unknown |
| Glyco_transf_24 domain-containing protein | A0A5G2Q988 | 0.416884 | 1 | 49 | 0.02689 | Transferase activity, transferring hexosyl groups |
| 60S ribosomal protein L7 (Fragment) | Q6QAS9 | 0.415317 | 1 | 73 | 0.03863 | MRNA binding, nuclear-transcribed mRNA catabolic process, nonsense-mediated decay |
| Uncharacterized protein | A0A4X1SHM4 | 0.414296 | 26 | 47 | 0.001758 | Function unknown |
| Sterol regulatory element-binding protein 1 | O97676 | 0.41369 | 2 | 2 | 0.0342 | Transcription factor activity, sequence-specific DNA binding; regulation of peptide secretion |
| Uncharacterized protein | A0A4X1USN7 | 0.413043 | 2 | 3 | 0.03445 | Zinc ion binding |
| Transcription elongation factor SPT5 | A0A5G2RNJ4 | 0.407706 | 3 | 38 | 0.0027 | Translation elongation factor activity |
| Tyrosine-protein kinase | A0A4X1V771 | 0.406538 | 1 | 6 | 0.02034 | Cellular protein modification process; negative regulation of RNA biosynthetic process |
| Beta-1,4-galactosyltransferase 5 | A0A5S8MJ01 | 0.405399 | 4 | 13 | 0.004927 | Udp-glycosyltransferase activity; lipid biosynthetic process |
| Translocase of outer mitochondrial membrane 6 | A0A287BEP4 | 0.404451 | 2 | 58 | 0.01959 | Function unknown |
| High density lipoprotein binding protein | A0A0B8S073 | 0.403893 | 1 | 51 | 0.02977 | RNA binding |
| Metalloproteinase inhibitor 3 | A0A286ZZX3 | 0.401149 | 3 | 12 | 0.0162 | Peptidase inhibitor activity; regulation of nitrogen compound metabolic process |
| Galectin | A0A286ZYZ8 | 0.396303 | 1 | 38 | 0.03571 | Binding |
| Metallophos domain-containing protein | I3LCM5 | 0.389688 | 2 | 7 | 0.02865 | Function unknown |
| BMP and activin membrane- | A0A480KI92 | 0.38939 | 1 | 3 | 0.003133 | Receptor binding; positive regulation of cell proliferation |

| | | | | | | |
|---|---|---|---|---|---|---|
| bound inhibitor homolog | | | | | | |
| Uncharacterized protein | A0A4X1V704 | 0.385394 | 1 | 1 | 0.03052 | Function unknown |
| Pirh2 | A1YJE5 | 0.383993 | 3 | 10 | 0.01642 | Zinc ion binding |
| Perilipin | R9WHX5 | 0.383354 | 15 | 34 | 0.003386 | Lipid particle, lipid transport |
| Peptidase A1 domain-containing protein | A0A4X1VM25 | 0.382744 | 1 | 2 | 0.000234 | Function unknown |
| Uncharacterized protein | A0A4X1U2C6 | 0.380626 | 1 | 3 | 0.00996 | Function unknown |
| Ras-related protein Rap-2a | Q06AU2 | 0.378881 | 6 | 63 | 0.01732 | Nucleoside-triphosphatase activity; regulation of phosphorus metabolic process |
| Protein-tyrosine sulfotransferase | A0A480JPS8 | 0.377604 | 2 | 5 | 0.03251 | Binding |
| Tudor domain-containing protein 7 | A0A4X1UP96 | 0.372806 | 5 | 5 | 0.02241 | Function unknown |
| Phospholipid-transporting ATPase | A0A4X1TLB7 | 0.372657 | 2 | 2 | 0.03372 | Function unknown |
| BZIP domain-containing protein | A0A4X1VLW8 | 0.371197 | 2 | 23 | 0.02678 | Double-stranded DNA binding |
| SHS2_Rpb7-N domain-containing protein | F1SC14 | 0.370794 | 5 | 18 | 0.03566 | DNA-directed RNA polymerase activity |
| HMG domain-containing protein 4 | A0A287B9A3 | 0.367993 | 1 | 3 | 0.02111 | Function unknown |
| Laminin subunit gamma-2 isoform a | F1S662 | 0.367503 | 36 | 38 | 0.03513 | Function unknown |
| Laminin subunit alpha-1 (Fragment) | A0A480XDX5 | 0.367029 | 1 | 17 | 0.01149 | Function unknown |
| Amino acid transporter | F1S763 | 0.365007 | 1 | 3 | 0.01887 | Function unknown |
| Signal transducer and | A0A4X1W8D9 | 0.364463 | 11 | 16 | 0.03709 | Transcription factor activity, sequence-specific DNA binding; immune effector process, |

| | | | | | | |
|---|---|---|---|---|---|---|
| activator of transcription | | | | | | regulation of protein phosphorylation |
| Ras-associating domain-containing protein | A0A4X1V4G5 | 0.363701 | 1 | 2 | 0.004983 | Function unknown |
| Calponin-homology (CH) domain-containing protein | A0A5G2QI59 | 0.362694 | 7 | 9 | 0.02106 | Function unknown |
| Phospholipid scramblase | I3LHI3 | 0.362388 | 4 | 20 | 0.04363 | Protein binding, organophosphate ester transport; sterol homeostasis |
| Daxx | A0A480ZCA1 | 0.361958 | 3 | 6 | 0.02276 | Function unknown |
| Prolyl 3-hydroxylase 1 isoform 1 | A0A480KI56 | 0.361897 | 1 | 29 | 0.02903 | Function unknown |
| ADAR adenosine deaminase RNA specific | K9J4M6 | 0.361793 | 43 | 46 | 0.00385 | RNA binding, RNA processing |
| Zinc finger MYM-type protein 1 isoform X1 | A0A481C188 | 0.361151 | 2 | 2 | 0.005405 | Function unknown |
| Uncharacterized protein | A0A287APC5 | 0.35985 | 3 | 8 | 0.005894 | Transcription regulator activity |
| Osteopontin | P14287 | 0.357729 | 10 | 40 | 0.0296 | Integrin binding; regulation of tissue remodeling, positive regulation of nucleobase-containing compound metabolic process |
| Heparin cofactor 2 | A0A480RKY5 | 0.35474 | 1 | 3 | 0.009598 | Extracellular space, cellular anatomical entity |
| Complex I-KFYI | A1XQS6 | 0.354278 | 2 | 24 | 0.009943 | Respiratory chain complex I; macromolecular complex |
| Phospholipid scramblase | A0A5G2QX76 | 0.353967 | 5 | 18 | 0.001525 | Function unknown |
| Eukaryotic translation initiation factor 3 subunit K isoform 1 | A0A481D1L8 | 0.353952 | 1 | 50 | 0.03555 | Translation initiation factor activity |
| AKAP2_C domain-containing protein | A0A287AMX5 | 0.352666 | 15 | 21 | 0.01165 | Function unknown |
| GMP_PDE_delta domain-containing protein | A0A5G2QW04 | 0.349534 | 1 | 20 | 0.0328 | Function unknown |
| Uncharacterized protein | F1SBB3 | 0.34926 | 33 | 12 | 0.02509 | Function unknown |

| | | | | | | |
|---|---|---|---|---|---|---|
| Semaphorin-4B isoform 1 | A0A287A766 | 0.349112 | 7 | 9 | 0.005664 | Function unknown |
| RING-type E3 ubiquitin transferase | A0A4X1V887 | 0.347805 | 11 | 27 | 0.01074 | Function unknown |
| Cytochrome P450 | A7KZR2 | 0.347549 | 2 | 46 | 0.02386 | Oxidoreductase activity |
| Dual specificity protein phosphatase | A0A5G2RAD0 | 0.347259 | 1 | 5 | 0.003963 | Function unknown |
| Uncharacterized protein | A0A4X1UQJ8 | 0.346955 | 2 | 10 | 0.01103 | Cellular component assembly; SMN complex |
| Uncharacterized protein | A0A5G2QI85 | 0.3458 | 10 | 68 | 0.009622 | Ribonucleotide binding, positive regulation of cellular process |
| Lysosome membrane protein 2 | F1RYT3 | 0.343871 | 10 | 25 | 0.004294 | Quaternary ammonium group binding; lysosome |
| Pleckstrin homology domain-containing family A member 1 isoform X7 | A0A480QEJ7 | 0.343142 | 4 | 18 | 0.001573 | Function unknown |
| Guanylate binding protein 2 | B2XWS2 | 0.342567 | 5 | 20 | 0.02755 | Ribonucleotide binding |
| G_PROTEIN_RECEP_F3_4 domain-containing protein | A0A4X1V7D7 | 0.342287 | 4 | 14 | 0.00606 | Function unknown |
| Dipeptidyl peptidase 4 | A0A5G2Q7G7 | 0.341889 | 17 | 20 | 0.006007 | Catalytic activity, acting on a protein; positive regulation of t cell activation |
| Solute carrier family 30 member 2 | B5A8K5 | 0.34146 | 1 | 3 | 0.01182 | Intrinsic component of membrane; cellular anatomical entity |
| Tuberin isoform 7 | A0A480W2V1 | 0.341397 | 2 | 6 | 0.03302 | Enzyme activator activity |
| Tripartite motif-containing protein 16 isoform a | A0A481BZY0 | 0.340576 | 8 | 21 | 0.002472 | Function unknown |
| ATPase H(+)-transporting lysosomal accessory protein 2 | A0A5G2QNQ8 | 0.335385 | 1 | 26 | 0.02403 | Function unknown |
| CCN family member 2 | F1S3P6 | 0.334895 | 6 | 20 | 0.03285 | Receptor binding; negative regulation of cellular process; signal transduction |
| Uncharacterized protein | A0A4X1UUL9 | 0.334598 | 1 | 30 | 0.03999 | Function unknown |

| | | | | | | |
|---|---|---|---|---|---|---|
| Uncharacterized protein | A0A286ZRH1 | 0.334329 | 23 | 27 | 0.004041 | Function unknown |
| Uncharacterized protein | A0A5G2RA88 | 0.333895 | 3 | 22 | 0.04709 | Protein localization; endoplasmic reticulum; peptide transport |
| Adenosine deaminase 2 | P58780 | 0.333895 | 2 | 7 | 0.01436 | Adenosine deaminase activity; biosynthetic process |
| Uncharacterized protein | A0A287A7A8 | 0.333093 | 4 | 4 | 0.03562 | Function unknown |
| CBFD_NFYB_HMF domain-containing protein | A0A287AUX2 | 0.33285 | 3 | 33 | 0.01331 | Heterocyclic compound binding |
| Dolichyl-diphosphooligosaccharide--protein glycosyltransferase subunit KCP2 | A0A4X1VZL2 | 0.33243 | 1 | 8 | 0.01074 | Function unknown |
| Caspase-10 isoform 1 preproprotein | A0A286ZWN2 | 0.33241 | 10 | 26 | 0.006527 | Peptidase activity, regulation of cell death |
| G patch domain-containing protein 4 isoform 2 | A0A481ASU0 | 0.332006 | 4 | 9 | 0.008409 | Heterocyclic compound binding |
| Epiplakin isoform X1-like (Fragment) | A0A480PQZ4 | 0.331814 | 14 | 30 | 0.009525 | Function unknown |
| Junctional adhesion molecule 1 | A0A5K1V5T2 | 0.327128 | 10 | 37 | 0.002172 | Function unknown |
| Uncharacterized protein | A0A4X1TWZ9 | 0.321851 | 6 | 2 | 0.04228 | Function unknown |
| G-protein-coupled receptor 39 | F1RY26 | 0.319627 | 5 | 10 | 0.01747 | Metal ion binding; intrinsic component of membrane |
| 2-5A-dependent ribonuclease | A0A286ZPJ7 | 0.317989 | 19 | 37 | 0.007735 | Phosphotransferase activity; alcohol group as acceptor |
| Nidogen-2 isoform X1 | A0A480MU29 | 0.316041 | 1 | 41 | 0.02777 | Function unknown |
| Uncharacterized protein | I3LJ31 | 0.313351 | 3 | 5 | 0.005211 | Integral component of membrane; cellular anatomical entity |
| Uncharacterized protein | A0A4X1TIC9 | 0.313076 | 1 | 21 | 0.00492 | Function unknown |
| Uncharacterized protein | A0A4X1U855 | 0.312799 | 7 | 17 | 0.01091 | Function unknown |
| RING-type domain-containing | A0A287B7H5 | 0.311564 | 1 | 2 | 0.01986 | Function unknown |

| | | | | | | |
|---|---|---|---|---|---|---|
| protein | | | | | | |
| V-type proton ATPase subunit a | A0A4X1STL1 | 0.310445 | 13 | 21 | 0.003733 | Function unknown |
| CW-type domain-containing protein | A0A5G2QF40 | 0.310284 | 8 | 10 | 0.02577 | Function unknown |
| Oxysterol-binding protein | A0A5G2QUB9 | 0.309421 | 1 | 3 | 0.04428 | Function unknown |
| AAA domain-containing protein | F2Z5H2 | 0.307856 | 13 | 48 | 0.000479 | Binding; positive regulation of endocytosis |
| Anaphase-promoting complex subunit 10 | D5L7X1 | 0.307211 | 3 | 22 | 0.04413 | Transferase complex; regulation of cell cycle process |
| Integrator complex subunit 14 | A0A5K1U644 | 0.306957 | 5 | 16 | 0.03426 | Function unknown |
| Uncharacterized protein | F1SED4 | 0.305812 | 14 | 50 | 0.002509 | Binding |
| Proteasome subunit beta | Q2PYM7 | 0.305577 | 6 | 37 | 0.04716 | Peptidase activity, acting on l-amino acid peptides; regulation of proteolysis |
| RUN and TBC1 domain-containing protein 3 | A0A480PGU9 | 0.305377 | 3 | 5 | 0.02247 | Function unknown |
| Transforming growth factor beta-1 proprotein | P07200 | 0.304799 | 9 | 27 | 0.001228 | Receptor binding; immune system process |
| N-acetyltransferase domain-containing protein | A0A4X1W7X4 | 0.303804 | 5 | 23 | 8.00E-05 | Transferase activity, transferring acyl groups other than amino-acyl groups |
| BTB domain-containing protein | A0A287B5G3 | 0.300476 | 7 | 14 | 0.03854 | Negative regulation of apoptotic signaling pathway; negative regulation of metabolic process |
| Phosphoenolpyruvate carboxykinase, cytosolic [GTP] | A0A4X1UM84 | 0.299862 | 12 | 32 | 0.007555 | Positive regulation of nitrogen compound metabolic process; response to chemical |
| Ribosomal_L7Ae domain-containing protein | I3LCJ7 | 0.299802 | 5 | 31 | 0.00829 | Organic cyclic compound binding; endonuclease complex |
| SAM_MT_RSMB_NOP | A0A4X1UJD8 | 0.298059 | 6 | 22 | 0.00133 | Binding |

| | | | | | | |
|---|---|---|---|---|---|---|
| domain-containing protein | | | | | | |
| Cytochrome c oxidase subunit | A0A287BGN0 | 0.297583 | 2 | 43 | 0.03747 | Hydrogen ion transmembrane transporter activity; mitochondrial protein complex |
| Acyl-coenzyme A oxidase | A0A5G2R961 | 0.29504 | 2 | 5 | 0.04846 | Function unknown |
| TBC1 domain family member 1 isoform X4 | I3LNR0 | 0.293977 | 6 | 6 | 0.00042 | Function unknown |
| Plectin (Fragment) | K9IVQ6 | 0.291673 | 1 | 71 | 0.008935 | Function unknown |
| Nucleolar transcription factor 1 isoform X1 | F1RQY4 | 0.288216 | 1 | 40 | 0.03351 | DNA binding; membrane-bounded organelle |
| Uncharacterized protein | A0A4X1UCR9 | 0.286767 | 24 | 43 | 0.004056 | Cytokine receptor activity |
| Formin-like protein 2 isoform X5 | A0A480RHY5 | 0.286477 | 14 | 19 | 0.02293 | Function unknown |
| Lipoprotein lipase | I3LUA0 | 0.286373 | 6 | 20 | 0.01412 | Triglyceride lipase activity; endosome; early endosome |
| IF rod domain-containing protein | A0A4X1WBS5 | 0.286095 | 2 | 10 | 0.000663 | Function unknown |
| Phosphate transporter | M3V824 | 0.285935 | 5 | 9 | 0.02096 | Function unknown |
| DNA helicase | A0A5K1VUK9 | 0.285876 | 23 | 19 | 0.003624 | Function unknown |
| Integrin-associated protein | A0A4X1SPG5 | 0.285161 | 2 | 14 | 0.03335 | Thrombospondin receptor activity; extracellular vesicle |
| TNF receptor-associated factor | A0A480SEQ9 | 0.284987 | 3 | 7 | 0.007375 | Function unknown |
| [Histone H3]-lysine(4) N-trimethyltransferase | A0A287A913 | 0.284839 | 1 | 0 | 0.03028 | Function unknown |
| Hexosyltransferase | A0A287AWP7 | 0.284049 | 3 | 7 | 0.02412 | Function unknown |
| Ubiquitinyl hydrolase 1 | F1RXF7 | 0.283781 | 8 | 38 | 0.04263 | Thiol-dependent ubiquitin-specific protease activity |
| Longin domain-containing protein | A0A287B7S4 | 0.282275 | 2 | 11 | 0.02907 | Function unknown |
| ADP/ATP translocase 3 | A0A287AXS7 | 0.282015 | 7 | 65 | 0.01064 | Adp transmembrane transporter activity |

| | | | | | | |
|---|---|---|---|---|---|---|
| AB hydrolase-1 domain-containing protein | A0A4X1VWB4 | 0.279886 | 2 | 9 | 0.0345 | Intrinsic component of membrane; cellular anatomical entity |
| Exocyst complex component | A0A480UT78 | 0.279845 | 4 | 8 | 0.002696 | Exocytic process; macromolecular complex |
| t-SNARE coiled-coil homology domain-containing protein | A0A4X1VQQ8 | 0.279353 | 2 | 20 | 0.003373 | Snare binding, endomembrane system |
| Inter-alpha-trypsin inhibitor heavy chain H2 | A0A5G2QEV5 | 0.278699 | 9 | 10 | 0.0156 | Serine-type endopeptidase inhibitor activity; oxoacid metabolic process |
| 3-hydroxy-3-methylglutaryl coenzyme A reductase | A0A4X1SQE9 | 0.27824 | 8 | 11 | 0.02539 | Function unknown |
| Sorting nexin-24 isoform X4 | A0A480Q2P1 | 0.277886 | 1 | 12 | 0.01988 | Binding |
| Uncharacterized protein | A0A4X1UTU8 | 0.275916 | 2 | 7 | 0.01086 | Function unknown |
| Hermansky-Pudlak syndrome 3 protein homolog | A5A772 | 0.27398 | 5 | 6 | 0.009781 | Organelle organization; pigmentation |
| ATP-binding cassette, sub-family C (CFTR/MRP), member 13 | K9J4N7 | 0.273938 | 20 | 22 | 0.008823 | Function unknown |
| Secretory carrier-associated membrane protein | A0A4X1VTN9 | 0.273283 | 3 | 14 | 0.003668 | Function unknown |
| Tumor protein p73 | A0A4X1WA24 | 0.271375 | 4 | 7 | 0.003205 | Function unknown |
| Lethal(2) giant larvae protein homolog 2 | A0A481CW44 | 0.270849 | 29 | 37 | 0.007905 | Function unknown |
| Uncharacterized protein | F1S1J8 | 0.270179 | 6 | 35 | 0.03383 | Function unknown |
| USP domain-containing protein | A0A4X1UCD6 | 0.270157 | 1 | 5 | 0.03346 | Peptidase activity, acting on l-amino acid peptides; cellular protein metabolic process |
| Uncharacterized protein | A0A286ZMQ7 | 0.269031 | 8 | 26 | 0.002548 | Function unknown |
| Uncharacterized protein | A0A4X1TDN4 | 0.265297 | 18 | 26 | 0.01732 | Protein binding |

| | | | | | | |
|---|---|---|---|---|---|---|
| Polynucleotide phosphorylase 1 | A0A287AMV5 | 0.264709 | 44 | 67 | 0.02717 | Organic cyclic compound binding; aromatic compound catabolic process; RNA processing |
| Uncharacterized protein | A0A4X1TMR0 | 0.264278 | 4 | 45 | 0.01409 | Acyl-coa hydrolase activity; purine nucleoside bisphosphate metabolic process |
| Tr-type G domain-containing protein | A0A4X1WBZ8 | 0.263849 | 21 | 32 | 0.04751 | Translation initiation factor activity |
| Uncharacterized protein | A0A4X1UBH5 | 0.263142 | 1 | 13 | 0.04905 | Function unknown |
| **Downregulated proteins in PK-15-infected cells (a total of 131 proteins)** | | | | | | |
| Glyceraldehyde-3-phosphate dehydrogenase | A0A076KRX0 | -1.03138 | 3 | 77 | 0.02485 | Regulation of translation; oxidoreductase activity, acting on the aldehyde or oxo group of donors |
| Cellular retinol binding protein 1 | Q53J08 | -0.672103 | 2 | 57 | 0.03177 | Terpenoid biosynthetic process; binding |
| SUI1 domain-containing protein | A0A5G2QLD8 | -0.635261 | 3 | 78 | 0.002729 | Regulation of translation; translation initiation factor activity |
| Olfactory receptor | A0A287AX70 | -0.608824 | 1 | 2 | 0.03294 | Signal transduction; olfactory receptor activity |
| Uncharacterized protein | A0A5G2QVV0 | -0.605594 | 1 | 68 | 0.03066 | Function unknown |
| SGF29 C-terminal domain-containing protein | A0A4X1U8F2 | -0.576793 | 4 | 25 | 0.000249 | Covalent chromatin modification; protein binding |
| Thiopurine S-methyltransferase | A0A481DB59 | -0.569263 | 2 | 74 | 0.007656 | Methylation; methyltransferase activity |
| Syndecan | A0A287B0S3 | -0.540026 | 3 | 9 | 0.000242 | Function unknown |
| Heterogeneous nuclear ribonucleoprotein K | A0A4X1UTE2 | -0.537037 | 2 | 62 | 0.04311 | Positive regulation of cellular process; transcription factor activity, sequence-specific DNA binding |
| Syndecan | F1S0L5 | -0.535399 | 5 | 21 | 0.007086 | Cellular_component:cellular_component; |
| Uncharacterized protein | A0A4X1VCI3 | -0.514081 | 1 | 7 | 0.02767 | Function unknown |
| TIMELESS-interacting protein | A0A287BGF4 | -0.511401 | 3 | 8 | 0.02252 | Negative regulation of cellular process |
| Thymosin beta-4 | Q95274 | -0.491832 | 3 | 59 | 0.003064 | Positive regulation of cellular process; protein binding |

| | | | | | | |
|---|---|---|---|---|---|---|
| Uncharacterized protein | A0A5G2QSQ7 | -0.479801 | 1 | 30 | 0.0225 | Response to stimulus; protein binding |
| Uncharacterized protein | A0A4X1VI11 | -0.471701 | 1 | 5 | 0.02426 | Function unknown |
| Integral membrane protein 2 | F1RK12 | -0.471287 | 2 | 73 | 0.02785 | Negative regulation of cellular process; binding |
| ATP-binding cassette sub-family F member 1 isoform X1 | A0A480K1E2 | -0.465445 | 2 | 46 | 0.01248 | Regulation of nitrogen compound metabolic process; ribonucleotide binding |
| Hydroperoxy icosatetraenoate dehydratase | F1SIE9 | -0.454951 | 13 | 27 | 0.001535 | Hydrogen peroxide metabolic process; binding |
| SPARC | P20112 | -0.454331 | 14 | 47 | 0.004519 | Calcium ion binding |
| Tyrosine-protein kinase receptor | A0A480U2S6 | -0.443617 | 3 | 5 | 0.01908 | Function unknown |
| Uncharacterized protein | A0A4X1V3D9 | -0.439777 | 6 | 53 | 0.02406 | Function unknown |
| Glucose-6-phosphate 1-dehydrogenase | A0A0B8RVW4 | -0.439304 | 2 | 68 | 0.04662 | Function unknown |
| Proteasome subunit alpha type | F2Z5N0 | -0.434372 | 3 | 59 | 0.0341 | Muscle tissue development; peptidase activity, acting on L-amino acid peptides |
| Hepatocyte nuclear factor 1-beta | A0A4X1T4P3 | -0.431195 | 1 | 24 | 0.02259 | Function unknown |
| DNA-directed RNA polymerases I, II, and III subunit RPABC5 | A0A4X1VYD0 | -0.428678 | 2 | 30 | 0.03602 | Aromatic compound biosynthetic process; zinc ion binding |
| 6-pyruvoyl tetrahydrobiopterin synthase | A0A4X1TBY4 | -0.42707 | 6 | 46 | 0.005413 | Organic hydroxy compound metabolic process; protein binding |
| Ribosomal protein S6 kinase | I3LS09 | -0.423196 | 2 | 3 | 0.003568 | Binding |
| Solute carrier family 3 member 1 | B2D2K8 | -0.421081 | 10 | 17 | 0.001091 | Function unknown |
| Hepatocyte nuclear factor 1- | A0A5G2R7E8 | -0.419994 | 1 | 19 | 0.02184 | Positive regulation of transcription, DNA-templated; heterocyclic compound binding |

| | | | | | | |
|---|---|---|---|---|---|---|
| beta | | | | | | |
| Gag protein | R4JTP7 | -0.4183 | 2 | 24 | 0.0376 | Symbiosis, encompassing mutualism through parasitism; binding |
| Uncharacterized protein | A0A5G2RKV1 | -0.401257 | 1 | 20 | 0.02429 | Function unknown |
| PKS_ER domain-containing protein | A0A4X1U010 | -0.399864 | 1 | 5 | 0.02424 | Metabolic process; protein dimerization activity |
| PCNA-associated factor | A0A5G2QZV8 | -0.398 | 1 | 6 | 0.008994 | Regulation of biological process |
| Autophagy-related protein 3 | A0A480PFQ0 | -0.392698 | 7 | 34 | 0.006294 | Negative regulation of cellular process; catalytic activity, acting on a protein |
| Glutathione transferase | F1RL37 | -0.392157 | 4 | 16 | 0.01781 | Organic substance metabolic process |
| Tetraspanin-13 | F1SEI8 | -0.391466 | 3 | 21 | 0.01072 | Function unknown |
| CBFD_NFYB_HMF domain-containing protein | A0A5G2QBZ0 | -0.387762 | 4 | 24 | 0.04324 | Regulation of macromolecule biosynthetic process; heterocyclic compound binding |
| APC-binding protein EB1 | F6Q3L0 | -0.385105 | 12 | 57 | 0.0161 | Positive regulation of cellular component biogenesis; tubulin binding |
| Envelope protein | Q6W4V3 | -0.38431 | 4 | 38 | 0.03044 | Cellular_component:cellular_component |
| Importin-7 | I3LDM3 | -0.38163 | 5 | 45 | 0.004492 | Response to stimulus; protein binding |
| VPS10 domain-containing protein | A0A287A1U0 | -0.378575 | 11 | 13 | 0.00172 | Function unknown |
| Rab GDP dissociation inhibitor | I3L893 | -0.373655 | 15 | 64 | 0.006841 | Negative regulation of cellular process; enzyme regulator activity |
| Eukaryotic translation initiation factor 4H | I3LRD5 | -0.372767 | 10 | 38 | 0.001385 | Multi-organism reproductive process; translation initiation factor activity |
| Uncharacterized protein | A0A286ZPG4 | -0.369254 | 2 | 68 | 0.0343 | Cellular component organization; protein binding |
| Protein phosphatase 1 regulatory subunit 14B | Q8MIK9 | -0.367976 | 3 | 48 | 0.02723 | Protein serine/threonine phosphatase inhibitor activity |
| Uncharacterized protein | A0A287APU9 | -0.366285 | 3 | 5 | 0.0314 | Function unknown |
| WD_REPEATS_REGION domain-containing protein | A0A4X1W8T2 | -0.366168 | 4 | 12 | 0.001112 | Function unknown |
| Protein S100 | A0A4X1VVF5 | -0.365378 | 4 | 34 | 0.0126 | Response to stimulus; metal ion binding |

| | | | | | | |
|---|---|---|---|---|---|---|
| Protein S100 (Fragment) | A0A480ZBF9 | -0.362396 | 5 | 29 | 0.01537 | Positive regulation of cellular process; receptor binding |
| Small EDRK-rich factor 1 isoform 2 | A0A480K1R7 | -0.360411 | 1 | 13 | 0.0115 | Function unknown |
| Uncharacterized protein | A0A4X1UY47 | -0.358777 | 3 | 4 | 0.003993 | Function unknown |
| Fatty acid-binding protein, heart | O02772 | -0.354665 | 8 | 86 | 0.008223 | Cellular_component:cytoplasm; binding |
| Fructose-bisphosphate aldolase | A0A4X1U602 | -0.349246 | 1 | 69 | 0.03048 | ATP generation from ADP; fructose-bisphosphate aldolase activity |
| Uncharacterized protein | A0A4X1SXE8 | -0.349142 | 3 | 28 | 0.001458 | Catabolic process; |
| Jupiter microtubule associated homolog 1 isoform 1 | A0A480VV84 | -0.347829 | 5 | 64 | 0.001733 | Function unknown |
| UBC core domain-containing protein | A0A5G2RGI4 | -0.345932 | 4 | 58 | 0.004976 | Function unknown |
| Proteasome assembly chaperone 1 | I3LT95 | -0.345219 | 6 | 29 | 0.03587 | Function unknown |
| ANK_REP_REGION domain-containing protein | A0A4X1VC59 | -0.343459 | 6 | 24 | 0.0416 | Response to endoplasmic reticulum stress |
| Low-density lipoprotein receptor-related protein 2 | C0HL13 | -0.341928 | 2 | 24 | 0.02891 | Function unknown |
| Heterogeneous nuclear ribonucleoprotein D0 isoform c | A0A480M1B4 | -0.341029 | 15 | 50 | 0.03272 | Molecular_function:organic cyclic compound binding; RNA binding |
| Mannose-1-phosphate guanyltransferase alpha | A0A480QK53 | -0.339951 | 3 | 27 | 0.04944 | Function unknown |
| PPP1R35_C domain-containing protein | A0A4X1UE31 | -0.33722 | 1 | 3 | 0.02551 | Negative regulation of cellular process; protein binding |
| Uncharacterized protein | A0A5G2RAN0 | -0.334439 | 8 | 22 | 0.004152 | Positive regulation of cellular process |

| | | | | | | |
|---|---|---|---|---|---|---|
| Uncharacterized protein | F1RRD3 | -0.33427 | 1 | 52 | 0.04782 | Function unknown |
| Annexin | F2Z5C1 | -0.334113 | 22 | 74 | 0.006475 | Response to stimulus; lipid binding |
| Uncharacterized protein | A0A4X1SLB5 | -0.333214 | 6 | 21 | 0.02839 | Function unknown |
| Peptidylprolyl isomerase | I3LR51 | -0.331864 | 11 | 43 | 0.02369 | Cellular_component:intracellular membrane-bounded organelle; isomerase activity |
| Small nuclear ribonucleoprotein Sm D3 | A0A4X1V3C3 | -0.331206 | 4 | 48 | 0.04849 | Protein binding |
| 4a-hydroxytetrahydrobiopterin dehydratase | A0A4X1TCL6 | -0.331086 | 3 | 37 | 0.01264 | Positive regulation of cellular process; phenylalanine 4-monooxygenase activity |
| Splicing factor, arginine/serine-rich 11 | Q19QT0 | -0.326117 | 5 | 13 | 0.001402 | RNA processing; RNA binding |
| DUF4604 domain-containing protein | A0A4X1TEH0 | -0.32309 | 1 | 7 | 0.02072 | Function unknown |
| Uncharacterized protein | A0A4X1TZ18 | -0.322717 | 1 | 26 | 0.005439 | Function unknown |
| Meiosis protein 5 homolog | A0A4X1TKV7 | -0.322413 | 3 | 22 | 0.001124 | Regulation of nitrogen compound metabolic process; ligand-dependent nuclear receptor transcription coactivator activity |
| Uncharacterized protein | I3LNG8 | -0.320585 | 1 | 65 | 0.01913 | Response to organic substance; binding |
| Phosphoglycerate mutase | A0A286ZQ31 | -0.31627 | 9 | 38 | 0.00292 | ATP generation from ADP; isomerase activity |
| 39S ribosomal protein L33, mitochondrial | A0A4X1TLP9 | -0.315596 | 3 | 49 | 0.03032 | Translation; structural constituent of ribosome |
| Dynamin-like 120 kDa protein, form S1 | F1SFG7 | -0.314733 | 1 | 52 | 0.02394 | Negative regulation of cellular process; pyrophosphatase activity |
| Craniofacial development protein 1 | A0A5G2R143 | -0.30966 | 7 | 36 | 0.03173 | Function unknown |
| Triosephosphate isomerase | A0A288CFT0 | -0.307356 | 19 | 77 | 0.03669 | Function unknown |
| Uncharacterized protein | A0A4X1TNF0 | -0.306645 | 6 | 80 | 0.02288 | Function unknown |
| Leukotriene A(4) hydrolase | K9J6K4 | -0.306507 | 12 | 47 | 0.000167 | Icosanoid biosynthetic process; zinc ion binding |

| | | | | | | |
|---|---|---|---|---|---|---|
| 5-oxoprolinase isoform X1 | A0A480MWT6 | -0.306491 | 1 | 19 | 0.01972 | Function unknown |
| NTF2 domain-containing protein | A0A4X1UHL0 | -0.305242 | 5 | 83 | 0.006007 | Positive regulation of cellular process; structural constituent of nuclear pore |
| Uncharacterized protein | A0A4X1U6L2 | -0.303812 | 2 | 1 | 0.007438 | Function unknown |
| HIT domain-containing protein | F1RKI3 | -0.303653 | 5 | 53 | 0.01566 | Positive regulation of cellular process; catalytic activity |
| 15-oxoprostaglandin 13-reductase | A0A287B7R3 | -0.303482 | 15 | 57 | 0.02019 | Function unknown |
| Uncharacterized protein | A0A287B283 | -0.301796 | 10 | 33 | 0.001642 | Function unknown |
| Uncharacterized protein | A0A4X1UC72 | -0.301053 | 7 | 31 | 0.005238 | Positive regulation of cellular process; repressing transcription factor binding |
| CRIP2 | A0A165DE67 | -0.300866 | 8 | 45 | 0.00391 | Molecular_function:cation binding; ion binding |
| Thioredoxin | P82460 | -0.299789 | 8 | 89 | 0.008625 | Negative regulation of cellular process; disulfide oxidoreductase activity |
| Coiled-coil domain-containing protein 72 | F2Z5V1 | -0.298137 | 2 | 30 | 0.003451 | Function unknown |
| Malic enzyme | A0A5G2RAJ8 | -0.297189 | 21 | 54 | 0.002204 | Small molecule metabolic process; malate dehydrogenase (decarboxylating |
| Actin-related protein 2/3 complex subunit 3 | B5APU8 | -0.296622 | 6 | 35 | 0.01568 | Arp2/3 complex-mediated actin nucleation; |
| J domain-containing protein | A0A5G2R2L7 | -0.29571 | 10 | 38 | 0.03685 | Cellular_component:intracellular membrane-bounded organelle; protein binding |
| Telomerase RNA component interacting RNAse isoform 1 | F1SEX4 | -0.294522 | 8 | 48 | 0.004331 | Aromatic compound catabolic process; nuclease activity |
| G_PROTEIN_RECEP_F2_4 domain-containing protein | F1RF36 | -0.294061 | 7 | 14 | 0.0264 | Function unknown |
| Annexin | F1SEN1 | -0.293648 | 21 | 74 | 0.005457 | Function unknown |
| EKC/KEOPS complex subunit GON7 | P0C8B5 | -0.293036 | 3 | 48 | 0.01694 | Cellular_component:cellular_component; |
| Hypoxanthine-guanine phosphoribosyltransferase | Q45FY6 | -0.292491 | 2 | 67 | 0.02459 | Glycosyl compound biosynthetic process; small molecule binding |

| | | | | | | |
|---|---|---|---|---|---|---|
| Dynamin GTPase | A0A287B878 | -0.291599 | 2 | 29 | 0.003584 | Function unknown |
| Uncharacterized protein | A0A287B8I3 | -0.291042 | 5 | 18 | 0.02272 | Function unknown |
| Protein archease | A0A4X1VY44 | -0.290657 | 2 | 16 | 0.0247 | Function unknown |
| Seryl-tRNA synthetase | A0A287BJI6 | -0.29056 | 27 | 53 | 0.01824 | Function unknown |
| Stathmin | Q6DUB7 | -0.289364 | 8 | 46 | 0.009694 | Positive regulation of cellular process; protein binding |
| Transaldolase | A0A287BQ72 | -0.287688 | 12 | 30 | 0.02121 | Function unknown |
| Calponin | A0A4X1VU33 | -0.287374 | 11 | 55 | 0.03544 | Function unknown |
| Uncharacterized protein | A0A4X1TSU7 | -0.286066 | 10 | 32 | 0.0114 | Function unknown |
| Threonyl-tRNA synthetase | A0A480F5A7 | -0.285505 | 1 | 53 | 0.006431 | Function unknown |
| 14_3_3 domain-containing protein | A0A4X1V4V6 | -0.285402 | 11 | 68 | 0.007724 | Positive regulation of cellular process; channel regulator activity |
| Uncharacterized protein | A0A4X1UBE7 | -0.284256 | 3 | 38 | 0.000358 | Function unknown |
| Uncharacterized protein | A0A286ZQ23 | -0.283241 | 12 | 32 | 0.01607 | Molecular_function:transferase activity, transferring phosphorus-containing groups; catalytic activity |
| ATP-binding cassette sub-family F member 1 isoform a | A0A480PQG3 | -0.282454 | 2 | 46 | 0.03874 | Regulation of nitrogen compound metabolic process; ribonucleotide binding |
| LRRcap domain-containing protein | A0A4X1TYN7 | -0.28197 | 9 | 34 | 0.03312 | Function unknown |
| Eukaryotic translation initiation factor 3 subunit E | F1S1G0 | -0.281832 | 23 | 60 | 0.03225 | Positive regulation of cellular process; translation factor activity, RNA binding |
| RING-type domain-containing protein | A0A4X1TVR4 | -0.281054 | 5 | 15 | 0.01428 | Positive regulation of cellular process; transition metal ion binding |
| Uncharacterized protein | I3LLQ7 | -0.280242 | 2 | 15 | 0.004602 | Function unknown |
| Annexin | A0A4X1W3B6 | -0.27781 | 24 | 68 | 0.005565 | Icosanoid secretion; |
| MPN domain-containing protein | A0A4X1UW55 | -0.276591 | 15 | 61 | 0.02333 | Positive regulation of cellular process; peptidase activity, acting on L-amino acid peptides |

| | | | | | | |
|---|---|---|---|---|---|---|
| Programmed cell death protein 5 | A0A481D4P9 | -0.27584 | 9 | 74 | 0.03116 | Positive regulation of cellular process; acetyltransferase activator activity |
| Tyrosine-protein phosphatase non-receptor type | A0A5G2QDD3 | -0.275827 | 1 | 48 | 0.01236 | Positive regulation of cellular process; insulin receptor binding |
| Cyclin-dependent kinases regulatory subunit | A0A4X1W0Z6 | -0.275326 | 2 | 33 | 0.01112 | Positive regulation of cellular process; enzyme regulator activity |
| Hsp90 chaperone protein kinase-targeting subunit | Q684M6 | -0.273493 | 14 | 43 | 0.004248 | Positive regulation of cellular process; protein binding |
| Peptidyl-prolyl cis-trans isomerase | A0A4X1W0F9 | -0.273478 | 5 | 51 | 0.003075 | Function unknown |
| PX domain-containing protein | A0A286ZKH8 | -0.273338 | 6 | 57 | 0.01169 | Negative regulation of cellular process; protein binding |
| Uncharacterized protein | F1SGZ8 | -0.27309 | 19 | 56 | 0.002509 | Regulation of cellular component size; protein binding |
| CCDC50_N domain-containing protein | A0A5G2QER5 | -0.272456 | 2 | 3 | 0.03041 | System process; |
| Transcription factor BTF3 | A0A287BJ88 | -0.271433 | 10 | 73 | 0.01123 | Multicellular organism development; |
| Cofilin-2 | Q5G6V9 | -0.270735 | 11 | 72 | 0.01743 | Positive regulation of cellular process; protein binding |
| Uncharacterized protein | A0A287B3B5 | -0.270464 | 3 | 5 | 0.001064 | Anatomical structure development; protein dimerization activity |
| Uncharacterized protein | A0A4X1U9S0 | -0.269536 | 2 | 11 | 0.02041 | Cellular component organization or biogenesis; |
| Uncharacterized protein | A0A4X1U210 | -0.269086 | 14 | 65 | 0.02894 | Signal transduction; phosphatidylglycerol binding |
