# Supplementary material for: Comparative Proteomic Analysis Reveals Mx1 Inhibits Senecavirus A Replication in PK-15 Cells by Interacting with the Capsid Proteins VP1, VP2 and VP3
Source: Viruses. 2022 Apr 21;14(5):863. doi: 10.3390/v14050863 (PMC9147370; doi:10.3390/v14050863)
Supplement: Supplementary file 1 [file viruses-14-00863-s001.zip › Supplementary Table S2.pdf]

Supplementary Table S2 The differentially expressed proteins identified by TMT labeling coupled with LC-MS/MS analysis of PK-15 cells in response to SVA infection at 24 hpi

Protein name	Uniport accession No.	Log_2 ratios (infection/control)	peptides	Sequence coverage (%)	P-values	functions
**Upregulated proteins in PK-15-infected cells (a total of 697 proteins)**						
Peptidase S1 domain-containing protein	A0A4X1SN34	2.095754	1	3	0.01089	Function unknown
Lumican	F1SQ09	2.002196	1	5	0.00604	Positive regulation of cytokine production;
SH3 domain-containing protein	A0A287AA14	1.762886	1	0	0.007784	Function unknown
Nebulin isoform X24	A0A480QQC5	1.688143	1	0	0.01349	Function unknown
DNA topoisomerase I	I3LTP0	1.667966	1	2	0.007448	Function unknown
Protein S100-A14 isoform X1	F1SFV3	1.637145	2	15	0.006909	Positive regulation of cellular process; binding
Interferon-induced GTP-binding protein Mx1	A0A4X1TKB5	1.616903	6	78	0.002908	Receptor metabolic process; catalytic activity
Uncharacterized protein	A0A4X1U802	1.539596	1	0	0.01051	Function unknown
Single-pass membrane and coiled-coil domain-containing protein 1 isoform 1	A0A287BI98	1.535366	1	6	0.001449	Function unknown
2'-5'-oligoadenylate synthase-like protein isoform a	F1RIV0	1.489223	13	44	0.0047	Defense response to other organism; ribonucleotide binding
RNA helicase	A0A4X1TN23	1.478805	1	3	0.04562	Helicase activity
Ubiquitin-like modifier	A0A287AB58	1.461105	6	50	0.000789	Positive regulation of cellular process; integrin binding

Interferon-stimulated protein 60	F1SCY2	1.455918	14	41	0.001934	Negative regulation of cellular process; protein binding
Plasminogen	P06867	1.421735	10	15	0.01642	peptidase activity, acting on L-amino acid peptides
Interferon-induced protein with tetratricopeptide repeats 2	K9J6I9	1.410217	18	34	0.005382	Cellular response to interferon-alpha;
Uncharacterized protein	A0A287AZI1	1.39369	2	9	0.007273	Function unknown
DNA_MISMATCH_REPAIR_2 domain-containing protein	I3L5Z8	1.368318	2	2	0.01564	Function unknown
Uncharacterized protein	A0A4X1V7R2	1.367009	1	0	0.01073	Function unknown
Interferon-stimulated protein 56	J7FIC7	1.366249	30	64	0.002728	Regulation of response to stress;
RNA helicase	A8CYB8	1.346067	31	41	0.002467	Positive regulation of cellular process; helicase activity
Endoplasmic reticulum chaperone BiP (Fragment)	P34935	1.324592	1	56	0.01383	Negative regulation of cellular process; protein domain specific binding
Uncharacterized protein	A0A4X1TPF1	1.323285	1	2	0.01742	Function unknown
2'-5' oligoadenylate synthase	Q56VQ1	1.278108	30	54	0.0112	Defense response to other organism; ribonucleotide binding
Uncharacterized protein	A0A5G2QLT7	1.258677	2	4	0.01937	Function unknown
FIP-RBD domain-containing protein	A0A4X1UDM9	1.258556	1	32	0.02606	Function unknown
Galectin	A0A287A342	1.25283	11	45	0.006949	Function unknown
Tnp_22_trimer domain-containing protein	A0A5G2RBQ9	1.2405	1	3	0.002476	Function unknown
TLDc domain-containing protein	I3LMG2	1.238744	1	45	0.01206	Function unknown
FAM184 domain-containing protein	I3LV88	1.236749	1	1	0.01433	Function unknown

C-C motif chemokine (Fragment)	A1YM33	1.23596	3	47	0.008926	Regulation of phospholipase activity; catalytic activity
Uncharacterized protein	A0A287B4N8	1.231525	5	40	0.00447	Function unknown
TCTP domain-containing protein	A0A4X1UEK2	1.217968	1	19	0.003942	Function unknown
CCDC66 domain-containing protein	I3L7C7	1.21674	1	1	0.009016	Function unknown
Uncharacterized protein	A0A4X1WD16	1.210578	1	6	0.01314	Function unknown
Peptidase A1 domain-containing protein	A0A4X1T5P4	1.206586	1	3	0.005824	Function unknown
Noelin-2 isoform 2	A0A480XA75	1.19701	1	2	0.01006	Positive regulation of cellular process;
Inflammatory response protein 6	A0A287B6T9	1.190487	14	44	0.01034	Positive regulation of cellular process; protein self-association
Exocyst complex component 2	A0A287AU70	1.171492	1	23	0.004869	Function unknown
GB1/RHD3-type G domain-containing protein	A0A287BSF6	1.14999	2	30	0.004196	Ribonucleotide binding
Vitronectin	P48819	1.146206	2	4	0.01448	Immune system process; scavenger receptor activity
Uncharacterized protein	A0A5G2QG99	1.1435	1	0	0.001562	Function unknown
CCHC-type domain-containing protein	F1SMW0	1.11876	2	3	0.0113	Function unknown
Caveolin	A0A286ZW86	1.118634	1	45	0.01698	Positive regulation of cellular process; inward rectifier potassium channel inhibitor activity
Interferon-induced GTP-binding protein Mx2	A0A480HPX5	1.112414	26	54	0.01405	Receptor metabolic process; small molecule binding
PH domain-containing protein	A0A287BS42	1.111361	1	1	0.01574	Regulation of biological process;
Actin-related protein 2	A0A4X1WAK2	1.108757	1	40	0.02414	Positive regulation of cellular process; anion binding
Uncharacterized protein	A0A4X1W7Q7	1.097529	1	2	0.008163	Function unknown

Apolipoprotein H	A0A140TAK8	1.089746	3	13	0.02638	Function unknown
Uncharacterized protein	A0A4X1V704	1.06655	1	1	0.009442	Function unknown
UBC core domain-containing protein	A0A5G2Q9I0	1.066198	6	79	0.002661	Ubiquitin-dependent protein catabolic process; ribonucleotide binding
Tubulin beta chain	A0A480M929	1.061327	1	81	0.04508	Positive regulation of cellular process; purine ribonucleoside binding
ANK_REP_REGION domain-containing protein	A0A4X1U612	1.054509	1	14	0.02359	Function unknown
Arf-GAP with Rho-GAP domain, ANK repeat and PH domain-containing protein 1 isoform c	A0A481BP67	1.053495	1	10	0.01588	Function unknown
TLDc domain-containing protein	A0A4X1UQ31	1.045302	7	43	0.006388	Function unknown
MHC class I antigen	V9PR55	1.043605	1	54	0.0176	Response to stimulus;
DALR anticodon-binding domain-containing protein 3 isoform 1	F1SKI7	1.039808	1	2	0.002969	Function unknown
Interferon-induced GTP-binding protein Mx1	A0A481BA72	1.038371	1	77	0.04084	Receptor metabolic process; catalytic activity
RanGAP1_C domain-containing protein	A0A5G2R6N4	1.038203	1	48	0.006909	Function unknown
Receptor-binding cancer antigen expressed on SiSo cells	F1S1I0	1.019673	2	16	0.01793	Cell death;
Uncharacterized protein	A0A4X1VBX5	1.017506	1	6	0.01983	Function unknown
Vascular smooth muscle	Q27HS3	1.015738	1	73	0.01209	Circulatory system process; binding

alpha-actin (Fragment)						
2'-5' oligoadenylate synthase	Q5MX78	1.007388	2	27	0.002862	Defense response to other organism; ribonucleotide binding
mRNA-capping enzyme	A0A4X1VEE3	1.006176	3	7	0.0306	Function unknown
Uncharacterized protein	A0A287A6X8	1.00551	1	2	0.02216	Negative regulation of cellular process; protein binding
Armadillo repeat-containing protein 4 isoform X3	A0A480MFY5	1.004878	2	2	0.01857	Function unknown
Tumor-associated calcium signal transducer 2	F1S7A4	1.004316	8	34	0.007059	Positive regulation of cellular process;
MAGE domain-containing protein	A0A5G2QV82	0.997196	1	7	0.0138	Function unknown
Transmembrane protein 147	A0A4X1TJ50	0.984298	2	13	0.03501	Function unknown
RanBP2-type domain-containing protein	A0A4X1US75	0.984069	1	2	0.009029	Function unknown
HECT domain-containing protein	A0A4X1U9D8	0.977991	25	33	0.001319	Function unknown
IRF tryptophan pentad repeat domain-containing protein	A0A5G2RH09	0.961219	6	20	0.001988	Transcription regulator activity
Uncharacterized protein	A0A5G2RNJ9	0.959059	1	2	0.03396	Function unknown
Uncharacterized protein	A0A287B916	0.957965	1	2	0.02765	Lymphocyte differentiation; heterocyclic compound binding
Calpain catalytic domain-containing protein	A0A5G2RIY4	0.954803	1	0	0.0447	Function unknown
Uncharacterized protein	F1SS06	0.941919	1	1	0.0113	Function unknown
Phosphoglycerate mutase	B5KJG2	0.9419	1	12	0.0149	ATP generation from ADP; isomerase activity
N-terminal kinase-like-binding protein 1	F1RSD3	0.939941	3	14	0.008499	Function unknown
RNA helicase	F1S0P6	0.932336	12	22	0.007348	Positive regulation of cellular process; anion binding

RNF111_N domain-containing protein	A0A4X1URL2	0.927131	1	6	0.01636	Function unknown
Uncharacterized protein	A0A4X1VY50	0.926466	1	1	0.005985	Function unknown
tRNA (guanine(10)-N2)-methyltransferase homolog	A0A287BI99	0.921938	2	4	0.04437	Methylation; heterocyclic compound binding
MHC class I alpha chain (Fragment)	L7UVI0	0.918987	1	15	0.02273	Function unknown
Gc-globulin	A0A4X1T9I2	0.916498	2	8	0.007916	Organic cyclic compound binding
2',3'-cyclic-nucleotide 3'-phosphodiesterase	A0A287A9L8	0.908866	26	59	0.007494	Nucleoside phosphate metabolic process; binding
Nuclear autoantigen Sp-100 isoform 4	R4L6V8	0.908035	2	28	0.03594	Function unknown
Pyruvate carboxyltransferase domain-containing protein	A0A4X1VY76	0.907145	1	53	0.006018	Function unknown
Uncharacterized protein	A0A4X1UW90	0.902167	27	18	0.002423	Function unknown
Uncharacterized protein	A0A5G2R653	0.89631	1	1	0.01648	Function unknown
Hyaluronoglucosaminidase	F1SJB9	0.896061	3	2	0.005127	Function unknown
GTPase-activating Rap/Ran-GAP domain-like protein 3 isoform X1	I3LIY1	0.89127	1	1	0.01545	Function unknown
Ig-like domain-containing protein	A0A5G2QW86	0.879192	2	44	0.009073	Biological adhesion;
GB1/RHD3-type G domain-containing protein	A0A4X1U8M2	0.875136	1	6	0.004142	Function unknown
Reverse transcriptase domain-containing protein	A0A5G2R2R5	0.871792	1	1	0.003504	Function unknown

G domain-containing protein	F1S9T2	0.867466	1	39	0.01867	GTP binding
RNA helicase	A0A287AKR1	0.862288	20	21	0.004663	Binding
E3 ubiquitin-protein ligase RNF213 isoform 3	A0A480YJW7	0.86219	27	34	0.003105	Function unknown
Receptor-transporting protein 4	A0A287B9T3	0.854579	5	12	0.01067	Function unknown
Uncharacterized protein	A0A5G2QYT9	0.853551	1	1	0.01099	Function unknown
Aminopeptidase	K9J4P1	0.85078	7	7	0.002282	Function unknown
Galectin-3-binding protein	A0A5G2RLP2	0.847301	13	34	0.003767	Function unknown
IF rod domain-containing protein	F1SGI2	0.839965	1	7	0.01498	Function unknown
Phospholipid-transporting ATPase	M3TYF0	0.836716	1	1	0.01124	Function unknown
UPAR/Ly6 domain-containing protein	A0A287B805	0.828444	3	45	0.0263	Function unknown
DNA-dependent activator of IFN-regulatory factor	B8XX91	0.826025	6	46	0.006158	Positive regulation of cellular process; organic cyclic compound binding
J domain-containing protein	A0A5G2R4I8	0.825097	1	42	0.008812	Function unknown
Zinc finger protein 555 isoform 2	A0A287A1D7	0.818426	1	6	0.007455	Function unknown
Glutamine--fructose-6-phosphate transaminase (isomerizing)	F1S5Q1	0.808733	2	12	0.01463	Fructose 6-phosphate metabolic process; binding
Uncharacterized protein	A0A4X1TX07	0.800669	2	14	0.006103	Function unknown
ATP-sulfurylase	A0A481BE86	0.797814	1	4	0.01205	Function unknown
Beta-2-microglobulin	A0A4X1VG41	0.793611	3	32	0.005311	Positive regulation of cellular process; protein binding

Transporter 2, ATP-binding cassette, sub-family B (MDR/TAP)	A5D9J7	0.792526	16	31	0.005508	Immune system process; ribonucleotide binding
Putative E3 ubiquitin-protein ligase HERC6 isoform 1	A0A480M1Q1	0.78479	1	33	0.004606	Function unknown
Protein PML isoform 2	A0A480JXC7	0.784643	2	50	0.002311	Function unknown
Guanylate binding protein 2	B2XWS2	0.780767	5	20	0.00843	Ribonucleotide binding
Tumor necrosis factor receptor superfamily member 5	Q8SQ34	0.77975	6	30	0.008496	Positive regulation of cellular process; signaling receptor activity
Thioredoxin domain-containing protein	A0A4X1V5Q8	0.772105	1	10	0.005117	Function unknown
Exonuclease domain-containing protein	A0A4X1W796	0.771227	7	46	0.02134	Function unknown
Uncharacterized protein	A0A286ZSL2	0.770362	1	36	0.01867	Function unknown
Uncharacterized protein	A0A4X1SE52	0.768832	1	42	0.002574	Localization;
Uncharacterized protein	A0A4X1U2C6	0.754016	1	3	0.012	Function unknown
Hemoglobin subunit epsilon	F1RII6	0.750849	1	13	0.0139	Hydrogen peroxide metabolic process; protein binding
Uncharacterized protein	F1RW93	0.749613	1	2	0.01987	Function unknown
Malic enzyme	A0A4X1UJU2	0.744403	1	65	0.005259	Function unknown
Aprotinin (Fragment)	B0LXF7	0.744352	1	26	0.03092	Enzyme regulator activity
VLIG-type G domain-containing protein	F1RMQ6	0.742851	9	4	0.000902	Function unknown
Olfactory receptor	A0A5G2QYK1	0.741943	1	2	0.009285	Function unknown
Heat shock protein HSP 90-alpha isoform 2-like	A0A480TUW8	0.735092	1	13	0.02327	Ribonucleotide binding
Hemoglobin subunit theta-1	K7GNR8	0.733608	1	11	0.03237	Hydrogen peroxide metabolic process; organic cyclic compound binding

Protein FMC1 homolog (Fragment)	A0A480XJB3	0.733483	4	26	0.00932	Regulation of lipid catabolic process;
MHC class I antigen (Fragment)	A0A3G5MTB4	0.731139	2	55	0.01274	Response to stimulus;
Protein MCM10 homolog	A0A287AY37	0.730859	3	3	0.01106	Function unknown
Uncharacterized protein	A0A4X1URS1	0.730638	1	42	0.01263	Function unknown
Uncharacterized protein	F2Z552	0.725114	1	8	0.005349	Protein transport; small molecule binding
Alpha-2-macroglobulin isoform a	A0A480SMD8	0.722901	4	2	0.02073	Function unknown
Apolipoprotein D	A0A5K1TWC1	0.720607	3	14	0.01074	Negative regulation of cellular process; small molecule binding
Uncharacterized protein	A0A287B544	0.720041	2	38	0.006339	Function unknown
Urokinase-type plasminogen activator	A0A286ZXK2	0.718426	9	24	0.004307	Function unknown
2'-5'-oligoadenylate synthase 1	Q29599	0.71791	3	45	0.001829	Negative regulation of viral process; 2'-5'-oli
VWFA domain-containing protein	A0A4X1SK62	0.716987	1	9	0.01315	Function unknown
ASD2 domain-containing protein	A0A4X1TVX4	0.715689	1	13	0.01975	Function unknown
Uncharacterized protein	A0A5G2QJA8	0.711525	1	1	0.01583	Function unknown
Calponin-homology (CH) domain-containing protein	A0A5G2QI59	0.71064	7	9	0.002304	Function unknown
Calponin 3, acidic (Fragment)	A4H2I3	0.709918	1	83	0.01701	Cellular component organization; calmodulin binding
MHC class I antigen (Fragment)	K9LRJ6	0.709883	3	42	0.003054	Response to stimulus;
Tetratricopeptide repeat	A0A287AVL3	0.70685	1	1	0.01101	Binding

protein 14 isoform a						
Splicing factor 1	A0A480TKX0	0.699274	4	34	0.006672	Function unknown
XIAP-associated factor 1	K9IVW8	0.69877	6	29	0.006508	Function unknown
TPR_REGION domain-containing protein	A0A4X1U2X7	0.6981	3	9	0.002928	Function unknown
C3-beta-c	I3LTB8	0.697496	4	2	0.008172	Positive regulation of cellular process; peptidase inhibitor activity
Proteasome subunit beta	Q09YA9	0.689807	7	33	0.002464	Macromolecule metabolic process; peptidase activity, acting on L-amino acid peptides
Uncharacterized protein	A0A287BNI5	0.689555	3	11	0.01749	Ion binding
Poly [ADP-ribose] polymerase	A0A4X1TD13	0.689547	45	31	0.01362	Function unknown
Uncharacterized protein	A0A5G2QK26	0.68795	1	1	0.008673	Function unknown
Tudor domain-containing protein 7	A0A4X1UP96	0.68377	5	5	0.002886	Function unknown
UBA domain-containing protein	F1RF72	0.679476	8	2	0.02902	Function unknown
Uncharacterized protein	A0A4X1T910	0.679297	1	44	0.01265	Function unknown
Uncharacterized protein	A0A287BKF1	0.667257	24	42	0.002159	Ribonucleotide binding
Brain-specific angiogenesis inhibitor 1-associated protein 2	A0A4X1UGQ7	0.665602	1	56	0.03568	Cellular component organization or biogenesis; binding, bridging
Insulin	P01315	0.661432	2	26	0.01765	Positive regulation of protein phosphorylation; hormone activity
GFO_IDH_MocA domain-containing protein	A0A4X1UEI6	0.657793	1	2	0.00988	Extracellular structure organization; oxidoreductase activity
Histone domain-containing protein	F2Z581	0.656549	1	40	0.02174	Function unknown
Tumor necrosis factor receptor superfamily member 10C	A0A480PT21	0.653754	1	6	0.008974	Function unknown
Protein-serine/threonine	A0A4X1U1Y2	0.653698	1	1	0.04828	Function unknown

phosphatase						
Uncharacterized protein	A0A4X1TA69	0.652608	1	4	0.0057	Function unknown
Importin N-terminal domain-containing protein	A0A4X1ST59	0.648874	1	43	0.01055	Response to stimulus; protein binding
RING-type domain-containing protein	A0A287B7H5	0.648472	1	2	0.01525	Function unknown
Uncharacterized protein	A0A4X1T205	0.644381	8	35	0.006028	Function unknown
Ig-like domain-containing protein	A0A287A1Y1	0.64352	6	23	0.007126	Antigen processing and presentation of peptide antigen;
Protein BANP	A0A481CG23	0.642619	2	6	0.01221	Function unknown
Uncharacterized protein	A0A5G2QTL6	0.638555	2	4	0.03239	Function unknown
Caspase 4, apoptosis-related cysteine peptidase	A0A0B8RT56	0.638371	9	28	0.006323	Function unknown
Gap junction protein	A0A4X1UIU1	0.637867	1	2	0.002131	Function unknown
Ubiquitin-like domain-containing protein	A0A286ZQW6	0.637211	2	10	0.04855	Function unknown
Protein fem-1 homolog B	I3L6T4	0.637055	5	10	0.008316	Morphogenesis of an epithelium; receptor binding
Reverse transcriptase domain-containing protein	A0A5G2QY09	0.635111	1	3	0.01252	Function unknown
Chimaerin	A0A287BPP5	0.632997	2	6	0.008372	Regulation of hydrolase activity; enzyme activator activity
Uncharacterized protein	A0A4X1V406	0.632853	20	37	0.004303	Peptide transport; ribonucleotide binding
PARP-1 binding protein	A0A5G2QYX1	0.632309	1	1	0.003179	Function unknown
Uncharacterized protein	F1RGY5	0.631977	2	3	0.03758	Function unknown
Guanine nucleotide-binding protein subunit gamma	A0A287AHC5	0.631851	1	16	0.0215	Signal transduction; protein binding
Uncharacterized protein	A0A4X1U6K0	0.62951	3	10	0.007294	Function unknown

Proteasome subunit beta	Q2PYM7	0.629085	6	37	0.004456	Protein catabolic process; peptidase activity, acting on L-amino acid peptides
Uncharacterized protein	A0A4X1U946	0.626116	1	2	0.02467	Trans-synaptic signaling; transmembrane transporter activity
Histone acetyltransferase	A0A4X1UTM8	0.625832	10	26	0.01251	Positive regulation of cellular process; DNA binding
Uncharacterized protein	A0A4X1T995	0.624912	1	0	0.001512	Cellular cation homeostasis; cadherin binding
Heme oxygenase 1	P32394	0.624023	1	47	0.02466	Positive regulation of cellular process; protein binding
Uncharacterized protein	F1RM98	0.622442	6	19	0.04238	Positive regulation of cellular process; protein binding
EEF1A lysine methyltransferase 1	A0A481DEK1	0.615495	4	21	0.002142	Function unknown
COAgulation factor III (Fragment)	A0A481D288	0.615129	3	15	0.01221	Positive regulation of cellular process; lipid binding
Voltage-dependent anion-selective channel protein 2	A0A4X1UZQ9	0.60917	1	15	0.03272	Function unknown
Protein FAM25A	K7N7E8	0.607621	1	8	0.02375	Function unknown
Nuclear body protein SP140 isoform 5	A0A480PYU5	0.6041	5	26	0.006619	Function unknown
Alpha-mannosidase	A0A4X1UCM9	0.603523	3	5	0.02163	Metabolic process; binding
GTSE1_N domain-containing protein	F1SM83	0.601089	2	3	0.005892	Function unknown
Laminin subunit gamma-2 isoform a	F1S662	0.600727	36	38	0.01031	Function unknown
Uncharacterized protein	A0A5G2R0A9	0.598038	23	55	0.004554	Function unknown
J domain-containing protein	A0A4X1VG11	0.596897	5	46	0.02307	Function unknown
Guanine nucleotide-binding protein subunit gamma	A0A4X1UZS2	0.591926	1	13	0.01169	Pyrophosphatase activity
Uncharacterized protein	A0A4X1U1R2	0.589318	3	14	0.004437	Lipid biosynthetic process;
SAM domain-containing	A0A4X1U5A2	0.585019	7	6	0.004913	Function unknown

protein						
Uncharacterized protein	F1SM15	0.584501	1	1	0.01584	Function unknown
Tubulin alpha chain	A0A480IZE0	0.584499	1	61	0.00046	Cytoskeleton organization; ribonucleotide binding
60S ribosomal protein L7 (Fragment)	Q6QAS9	0.58128	1	73	0.008241	RNA processing; mRNA binding
Uncharacterized protein	A0A4X1V1X7	0.578954	3	5	0.008549	Function unknown
MHC class I antigen	A1YH92	0.578221	4	22	0.01086	Function unknown
Clusterin	A0A5S8KLN1	0.57565	12	34	0.01098	Positive regulation of cellular process; receptor binding
Epithelial-stromal interaction protein 1 isoform 2	A0A286ZSG6	0.572979	9	34	0.01382	Function unknown
Cytoplasmic protein	B6E314	0.570357	7	21	0.006	Positive regulation of cellular process; scaffold protein binding
Uncharacterized protein	A0A4X1SWR6	0.569595	1	6	0.008597	Function unknown
Uncharacterized protein	A0A287A746	0.569388	3	15	0.002844	Ion binding
Gap junction alpha-1 protein	Q29101	0.569258	1	2	0.003219	Regulation of hormone secretion; gap junction channel activity involved in cell communication by electrical coupling
Inhibitor_Mig-6 domain-containing protein	A0A287A7S3	0.568911	2	4	0.007559	Function unknown
Uncharacterized protein	I3L5Z9	0.56788	60	42	0.003027	Function unknown
LRRC37AB_C domain-containing protein	A0A286ZIV7	0.567458	2	2	0.01504	Function unknown
Letm1 RBD domain-containing protein	A0A286ZLJ0	0.561894	6	21	0.01742	Function unknown
IFITM2	F4ZS15	0.561801	1	28	0.004448	Function unknown
Beta actin (Fragment)	Q00P29	0.556737	2	76	0.01765	Cellular process; ribonucleotide binding
Z-DNA-binding protein 1	A0A481BW17	0.555088	2	39	0.0258	Function unknown
Protein kinase domain-	A0A4X1V4D9	0.554621	2	2	0.04161	Function unknown

containing protein						
Uncharacterized protein	A0A287BDP1	0.553491	1	25	0.008122	Function unknown
Pyroglutamyl-peptidase I	A0A4X1U7H9	0.553212	2	14	0.01245	Proteolysis; cysteine-type peptidase activity
Ras-related protein Rab-4	A0A287AKG6	0.551806	3	22	0.03473	Protein transport; binding
GTP_EFTU_D3 domain-containing protein	A0A287AVL5	0.550003	1	10	0.03904	Function unknown
G_PROTEIN_RECEP_F3_4 domain-containing protein	A0A4X1V7D7	0.54899	4	14	0.009145	Function unknown
Proline dehydrogenase	M3V806	0.547023	8	18	0.01122	Function unknown
Component of oligomeric Golgi complex 2	A0A480K7Y8	0.546559	10	17	0.004555	Function unknown
Nitric-oxide synthase (NADPH) (Fragment)	B4YYD8	0.546437	1	1	0.02967	Nitric oxide metabolic process; ribonucleotide binding
Tripartite motif-containing protein 26	B6ICV1	0.546423	5	11	0.01711	Binding
MYND-type domain-containing protein	A0A4X1VY99	0.545046	5	18	0.004317	Function unknown
Serine incorporator 3	A0A480PEC3	0.542177	2	3	0.01381	Function unknown
AMMECR1 domain-containing protein	A0A4X1VSW4	0.541137	3	17	0.04605	Function unknown
NHL repeat-containing protein 3 isoform a	A0A480W0M6	0.540654	3	12	0.02236	Function unknown
Beta-1,4-N-acetylgalactosaminyltransferase	A0A4X1UFA5	0.539373	2	3	0.01853	Function unknown
Metalloproteinase inhibitor 3	A0A286ZZX3	0.539097	3	12	0.007234	Positive regulation of cellular process; peptidase inhibitor activity

Interferon regulatory factor 7	X2KPB3	0.537999	2	5	0.04072	Positive regulation of cellular process; transcription factor activity, sequence-specific DNA binding
Vitellogenin domain-containing protein	F1SCV9	0.536874	16	3	0.01622	Function unknown
Plasminogen activator	A0A4X1U9U8	0.53583	10	27	0.001867	Function unknown
Integrator complex subunit 14	A0A5K1U644	0.533571	5	16	0.01616	Function unknown
Prothrombin	A0A4X1VLZ9	0.533522	6	9	0.008014	Response to stimulus; peptidase activity, acting on L-amino acid peptides
Pleckstrin homology-like domain family A member 1	F1SGD8	0.530407	1	2	0.02872	Function unknown
Sphingosine-1-phosphate phosphatase 2 isoform a	A0A5G2QIU3	0.529836	5	13	0.018	Function unknown
CD70 antigen	Q3ZDR4	0.529013	1	7	0.02025	Response to stimulus; binding
Translocon-associated protein subunit beta	A0A480W8D2	0.528602	1	3	0.04446	Multicellular organism development;
Uncharacterized protein	A0A2C9F343	0.527574	1	43	0.01705	Function unknown
Uncharacterized protein	A0A5G2QI85	0.526904	10	68	0.002498	Positive regulation of cellular process; ribonucleotide binding
Perilipin	R9WHX5	0.526439	15	34	0.007051	Anion transport;
Uncharacterized protein	A0A4X1SEB3	0.523593	13	24	0.00927	Function unknown
Galectin	A0A286ZYZ8	0.522899	1	38	0.03238	Binding
Carbohydrate sulfotransferase	A0A4X1TJF6	0.522671	1	2	0.01961	Function unknown
Cytospin-A	A0A287B7A6	0.521064	4	5	0.02808	Function unknown
ANK_REP_REGION domain-containing protein	A0A4X1URX0	0.520407	1	1	0.007897	Function unknown
Uncharacterized protein	A0A4X1SEZ6	0.517556	4	29	0.04834	Positive regulation of cellular process; BH domain binding
Uncharacterized protein	A0A288CG62	0.516676	2	4	0.01819	Function unknown
Lethal(3)malignant brain	A0A480QFR5	0.516194	2	2	0.04982	Myeloid cell differentiation; binding

tumor-like protein 3 isoform X2						
Endoribonuclease	F1RSL5	0.515066	2	3	0.001525	Function unknown
Uncharacterized protein	A0A4X1UQJ8	0.51333	2	10	0.02078	Function unknown
HAUS6_N domain-containing protein	A0A5G2QNR3	0.512145	8	12	0.02495	Cellular component organization or biogenesis;
Ras-associating domain-containing protein	F1SD18	0.50894	1	5	0.01616	Function unknown
CCR4-NOT transcription complex subunit 9	A0A5G2QHJ4	0.507578	9	34	0.004468	Aromatic compound catabolic process;
SERPIN domain-containing protein	A0A4X1U844	0.50179	7	20	0.04637	Function unknown
High affinity copper uptake protein 1	Q8WNR0	0.501739	2	16	0.003504	Metal ion transport; protein binding
Protein Spindly	A0A4X1U736	0.501647	5	13	0.005131	Function unknown
PH domain-containing protein	A0A4X1VUK6	0.501609	8	13	0.006281	Function unknown
Uncharacterized protein	A0A4X1SHM4	0.501607	26	47	0.001765	Function unknown
Kinesin-like protein	A0A4X1TA22	0.501467	6	12	0.02411	Microtubule-based movement; ribonucleotide binding
SAP130_C domain-containing protein	A0A5G2QAV4	0.500805	3	4	0.03762	Function unknown
SHS2_Rpb7-N domain-containing protein	F1SC14	0.498287	5	18	0.01513	Transcription, DNA-templated; DNA-directed RNA polymerase activity
Myeloid cell nuclear differentiation antigen	A0A287AP08	0.497784	4	10	0.02617	Function unknown
Uncharacterized protein	F1SL31	0.497709	1	34	0.02628	Function unknown
Uncharacterized protein	A0A5G2QH68	0.497234	1	5	0.00731	Function unknown
Phosphodiesterase	A0A4X1VR14	0.495354	1	1	0.008118	Regulation of cellular process; binding

RING-type E3 ubiquitin transferase	A0A287B425	0.494488	1	8	0.04555	Function unknown
Glyco_transf_24 domain-containing protein	A0A5G2Q988	0.492664	1	49	0.02776	Protein modification process; transferase activity, transferring hexosyl groups
Chitinase	A0A4X1TAK5	0.492333	1	3	0.04343	Function unknown
Ribosomal_L7Ae domain-containing protein	I3LCJ7	0.490658	5	31	0.002925	RNA processing; organic cyclic compound binding
Uncharacterized protein	A0A4X1UUL9	0.489252	1	30	0.01451	Function unknown
BTB domain-containing protein	A0A287B5G3	0.489103	7	14	0.01527	Negative regulation of cell communication;
Endo/exonuclease/phosphatase domain-containing protein	A0A4X1V6E3	0.488996	1	2	0.02746	Function unknown
Uncharacterized protein	A0A4X1UNF8	0.487309	6	11	0.04788	Signal transduction; nucleoside-triphosphatase regulator activity
Uncharacterized protein	F1S1J3	0.486339	3	8	0.01053	Function unknown
Zinc finger protein 75D isoform 1	A0A480WRF9	0.485954	2	6	0.005528	Transcription regulator activity
Uncharacterized protein	A0A4X1VFS0	0.485522	4	11	0.01376	Metal ion transmembrane transporter activity
ADP-ribosylation factor-like protein 6-interacting protein 1	K9IVS1	0.484915	3	23	0.01131	Positive regulation of cellular process; protein binding
Phosphatidylinositol 3-kinase 85 kDa regulatory subunit alpha	A0A480WYP1	0.483343	8	12	0.0156	Function unknown
Nociceptin	A0A4X1VC77	0.483052	1	4	0.03301	Trans-synaptic signaling; receptor binding
Uncharacterized protein	K7GR94	0.483037	7	22	0.01187	Function unknown
Uncharacterized protein	A0A4X1U128	0.482916	7	5	0.03081	Regulation of dendrite morphogenesis; nucleoside-triphosphatase regulator activity
Selenide, water dikinase 2	A1YIZ1	0.481357	5	18	0.01293	Small molecule metabolic process; ribonucleotide binding

Integrin-associated protein	A0A4X1SPG5	0.480792	2	14	0.03833	Positive regulation of cellular process; thrombospondin receptor activity
Arylamine N-acetyltransferase	A0A480RQR3	0.480345	1	41	0.009758	Function unknown
Ras-specific guanine nucleotide-releasing factor RalGPS2 isoform X1	F1S6Y4	0.479958	2	3	0.02502	Regulation of intracellular signal transduction; binding
3-hydroxy-3-methylglutaryl coenzyme A reductase	A0A4X1SQE9	0.478816	8	11	0.02141	Function unknown
Aminopeptidase	K9J6I5	0.478772	3	38	0.0296	Function unknown
Tr-type G domain-containing protein	A0A4X1WBZ8	0.478177	21	32	0.002029	Translational initiation; translation initiation factor activity
Uncharacterized protein	A0A4X1TDI0	0.475825	5	5	0.004227	Negative regulation of cellular process; enzyme activator activity
UBC core domain-containing protein	A0A4X1U068	0.475565	9	66	0.006022	Positive regulation of cellular process; small molecule binding
Signal peptidase complex catalytic subunit SEC11	A0A481CTH0	0.472836	5	18	0.01137	Peptide metabolic process; peptidase activity, acting on L-amino acid peptides
Uncharacterized protein	A0A5G2Q6W7	0.47243	6	11	0.009123	Biological adhesion;
Cyclin B1 transcript variant 1	D2IE28	0.472165	6	19	0.005151	Positive regulation of cellular process; catalytic activity, acting on a protein
Elongation of very long chain fatty acids protein (Fragment)	B3VHV3	0.472039	2	6	0.01382	Fatty acid elongation, saturated fatty acid; 3-oxo-lignoceronyl-COA synthase activity
Tsukushin	A0A480V1E4	0.469185	1	8	0.03375	Negative regulation of cellular process;
Uncharacterized protein	A0A5G2QJ57	0.468477	2	5	0.008982	Function unknown
Ras-related protein Rap-2a	Q06AU2	0.468207	6	63	0.004798	Positive regulation of cellular process; nucleoside-triphosphatase activity
Eyes absent homolog	A0A4X1TSB9	0.467345	1	12	0.003146	Function unknown
GMP reductase	A0A4X1VQX6	0.467062	6	37	0.003135	Carbohydrate derivative metabolic process; oxidoreductase activity, acting on NAD(P
Protein kinase domain-containing protein	A0A287AK13	0.467013	4	7	0.01534	Function unknown

Uncharacterized protein	A0A287A841	0.466915	2	34	0.003472	Function unknown
Myotubularin-related protein 11 isoform a	A0A480EMZ5	0.465595	2	3	0.03774	Function unknown
Zinc finger protein 318 (Fragment)	H9LC64	0.464868	1	2	0.01094	Positive regulation of transcription, DNA-templated; protein dimerization activity
HTH La-type RNA-binding domain-containing protein	A0A4X1SXY3	0.462162	2	5	0.03114	Regulation of translation; heterocyclic compound binding
Uridine-cytidine kinase	A0A4X1T9P1	0.462136	2	13	0.02419	Phosphorus metabolic process; small molecule binding
tRNA pseudouridine synthase	F1RJE9	0.462094	3	16	0.001417	RNA processing; catalytic activity, acting on a tRNA
ATP-dependent dihydroxyacetone kinase	A0A481D2Y6	0.460821	1	49	0.03863	Negative regulation of cellular process; small molecule binding
Histone-lysine N-methyltransferase, H3 lysine-79 specific	A0A4X1VSM5	0.460697	3	2	0.000932	Function unknown
Eukaryotic translation initiation factor 1A domain-containing protein	F1RU32	0.460416	6	46	0.000608	Translation initiation factor activity
Uncharacterized protein	A0A4X1USN7	0.459772	2	3	0.03996	Zinc ion binding
Uncharacterized protein	A0A5G2QQ23	0.458332	3	8	0.04624	Function unknown
Serine/threonine-protein kinase RIO1	A0A4X1SJG2	0.456584	10	23	0.005579	Function unknown
Tetraspanin	F1SKA6	0.456549	1	2	0.01583	Function unknown
RING-type E3 ubiquitin transferase	I3LRN0	0.456405	31	46	0.006452	Function unknown
BLOC-1-related complex subunit 7	A0A4X1TT04	0.45605	1	4	0.02869	Function unknown

Centromere protein X	A0A5G2QZG8	0.452783	3	21	0.01167	Function unknown
Mediator of RNA polymerase II transcription subunit 31	A0A4X1UL35	0.452257	1	13	0.003928	Negative regulation of cellular process; transcription regulator activity
IRF tryptophan pentad repeat domain-containing protein	A0A287B4G6	0.451938	2	4	0.009961	Defense response to other organism; RNA polymerase II transcription factor activity, sequence-specific DNA binding
Daxx	A0A480ZCA1	0.45187	3	6	0.001048	Function unknown
Uncharacterized protein	A0A5G2QG05	0.450502	3	11	0.01634	UDP-glycosyltransferase activity
Uncharacterized protein	A0A4X1TY47	0.45045	2	13	0.04168	Vesicle-mediated transport to the plasma membrane;
Uncharacterized protein	A0A4X1VWJ9	0.449471	2	23	0.03409	Function unknown
2-aminoethanethiol (Cysteamine) dioxygenase	F1RFB8	0.44526	6	34	0.008605	Oxidoreductase activity
TBC1 domain family member 10B	A0A480HHS2	0.444695	5	7	0.01699	Function unknown
TBC1 domain family member 20	A0A287A634	0.443482	2	7	0.01015	Positive regulation of cellular process; enzyme regulator activity
Uncharacterized protein	F1RY93	0.44232	2	7	0.01454	Ribonucleotide binding
Uncharacterized protein	A0A5G2QCE4	0.441123	1	0	0.04386	Function unknown
Spindle and kinetochore-associated protein 1	A0A4X1UHG2	0.440104	2	10	0.02395	Regulation of microtubule cytoskeleton organization; protein binding
COMM domain-containing protein 2 (Fragment)	A0A480M9T9	0.439147	3	21	0.000176	Function unknown
Pirh2	A1YJE5	0.438708	3	10	0.01085	Zinc ion binding
Plectin isoform X1-like (Fragment)	A0A480PNR4	0.437272	5	72	0.000423	Function unknown
Lamin isoform C	A0A480V981	0.437257	1	74	0.0278	Positive regulation of cellular process;
Dolichyl-	A0A4X1VZL2	0.437045	1	8	0.04047	Function unknown

diphosphooligosaccharide--protein glycosyltransferase subunit KCP2						
Uncharacterized protein	A0A287AD66	0.436809	12	28	0.01049	Function unknown
Uncharacterized protein	A0A286ZRH1	0.436629	23	27	0.01042	Function unknown
Tetraspanin	A0A480XE15	0.434032	2	12	0.01525	Function unknown
RING-type E3 ubiquitin transferase	A0A4X1V887	0.433298	11	27	0.000711	Function unknown
Uncharacterized protein	F1SBB3	0.432984	33	12	0.02414	Function unknown
Signal transducer and activator of transcription	A0A4X1W8D9	0.432841	11	16	0.009275	Positive regulation of cellular process; transcription factor activity, sequence-specific DNA binding
NEDD8 ultimate buster 1 isoform X1	A0A287A5K2	0.432701	18	29	0.001762	Regulation of proteolysis;
Mitochondrial genome maintenance exonuclease 1	A0A4X1UZ36	0.431877	11	45	0.009679	Function unknown
Protein kinase domain-containing protein	F1SA79	0.431855	2	5	0.004748	Function unknown
High density lipoprotein binding protein	A0A0B8S073	0.431185	1	51	0.03245	RNA binding
Tetratricopeptide repeat protein 4 isoform 1	A0A480WZ89	0.430387	2	5	0.03095	Function unknown
WD_REPEATS_REGION domain-containing protein	A0A286ZJE5	0.427908	2	7	0.03252	Function unknown
Integrin-associated protein	A0A287BQR8	0.427504	2	20	0.006455	Positive regulation of cellular process; thrombospondin receptor activity
Fibroblast growth factor-inducible 14	B7SLY2	0.42709	2	23	0.04116	Positive regulation of cellular process;

UBC core domain-containing protein	A0A4X1VUG6	0.427039	4	14	0.004627	Binding
Interferon regulatory factor 6	E9LK28	0.426388	4	12	0.01968	Regulation of nitrogen compound metabolic process; RNA polymerase II transcription factor activity, sequence-specific DNA binding
Uncharacterized protein	F1RUN3	0.423591	2	5	0.01344	Intracellular protein transport; molecular carrier activity
RNA-binding protein 12	I3LLW0	0.422556	2	16	0.006122	Binding
Phosphoinositide phospholipase C	A0A480J5N4	0.421995	11	21	0.008664	Function unknown
Max-like protein X isoform gamma	F1S1D8	0.421918	3	17	0.03145	Regulation of transcription from RNA polymerase II promoter; protein dimerization activity
Threonine synthase-like 2	I3L5P4	0.420544	4	12	0.03255	Function unknown
Uncharacterized protein	A0A287BGU5	0.420386	1	52	0.01861	MRNA processing; poly-purine tract binding
Beta-galactoside-binding lectin	Q7M2S1	0.41883	1	77	0.02667	Function unknown
Claudin	C3VML0	0.418121	3	21	0.02533	Positive regulation of cellular process; transmembrane transporter activity
Nudix hydrolase domain-containing protein	F1RUP7	0.417357	6	36	0.006537	Catalytic activity
Uncharacterized protein	A0A287AIG3	0.416925	2	10	0.006219	Function unknown
Uncharacterized protein	A0A4X1TBN2	0.416648	1	1	0.04652	Function unknown
Alkylated DNA repair protein alkB homolog 8	I3LA93	0.416636	8	18	0.001743	Function unknown
Uncharacterized protein	A0A4X1TDN4	0.415118	18	26	0.04776	Outer dynein arm assembly; protein binding
Uncharacterized protein	A0A5G2R1G6	0.412415	4	53	0.01926	Function unknown
Tyrosine 3/tryptophan 5-monooxygenase activation protein zeta polypeptide (Fragment)	Q00P21	0.411388	1	46	0.03882	Regulation of nitrogen compound metabolic process;

Phospholipid scramblase	I3LHI3	0.410767	4	20	0.02548	Sterol homeostasis; protein binding
ILEI domain-containing protein	A0A5G2R617	0.41007	4	21	0.02499	Function unknown
Uncharacterized protein	I3LKY5	0.409672	14	55	0.007143	Function unknown
Protein FAM173A isoform 1	A0A481BZ18	0.409438	6	40	0.01832	Protein methyltransferase activity
RNAse_Zc3h12a domain-containing protein	A0A4X1STC0	0.407979	1	8	0.01467	Function unknown
Uncharacterized protein	A0A4X1THV0	0.407291	3	39	0.007339	Function unknown
ELAV-like protein	A0A4X1WDI2	0.407127	4	13	0.01569	Binding
Ig-like domain-containing protein	A0A4X1VGD3	0.406984	1	4	0.02367	Function unknown
ER membrane protein complex subunit 10	A0A480I708	0.406619	3	21	0.038	Function unknown
Ubiquitinyl hydrolase 1	A0A4X1SI54	0.406411	3	16	0.001159	Macromolecule metabolic process; protein binding
Uncharacterized protein	A0A5G2R8T2	0.406233	2	7	0.03379	Function unknown
Receptor protein-tyrosine kinase	F1SUT8	0.406043	23	37	0.03996	Positive regulation of cellular process; transmembrane receptor protein kinase activity
Uncharacterized protein	F1RNK3	0.40556	4	16	0.01227	Function unknown
Uncharacterized protein	I3LB77	0.404818	5	17	0.01193	Positive regulation of cellular process; mRNA 3'-UTR binding
rRNA adenine N(6)-methyltransferase	A0A480VKC7	0.40369	6	32	0.01159	Binding
SEC7 domain-containing protein	A0A4X1U1Q3	0.40361	1	2	0.02757	Function unknown
ADAR adenosine deaminase RNA specific	K9J4M6	0.403247	43	46	0.02206	RNA processing; RNA binding
Ubiquitin carboxyl-terminal hydrolase	A0A4X1UQ80	0.401956	12	17	0.01977	Function unknown

RRM domain-containing protein	A0A5G2QT30	0.401688	1	7	0.02203	Function unknown
CW-type domain-containing protein	A0A5G2QF40	0.399677	8	10	0.01386	Function unknown
SERPIN domain-containing protein	A0A4X1U6E7	0.398961	7	17	0.01668	Function unknown
PHD-type domain-containing protein	F1RK19	0.397678	7	21	0.03805	Metal ion binding
Arrestin domain-containing protein 3 isoform a	A0A480YTQ3	0.397422	2	4	0.04763	Positive regulation of cellular process; receptor binding
UPF0669 protein C6orf120 isoform 2	I3LL77	0.395113	2	17	0.01395	Function unknown
Mitotic interactor and substrate of PLK1	A0A287ALE7	0.393942	8	11	0.006481	Function unknown
Hepatocyte growth factor receptor	Q2QLE0	0.392729	33	33	0.01336	Positive regulation of cellular component biogenesis; transmembrane signaling receptor activity
Dynactin subunit 5	A0A286ZK88	0.392721	2	10	0.002037	Artery development;
Uncharacterized protein	F1RJ87	0.392602	5	16	0.02094	Translational termination; translation release factor activity
Cysteine protease	A0A5G2Q8U4	0.391922	5	25	0.03793	Process utilizing autophagic mechanism; cysteine-type endopeptidase activity
Uncharacterized protein	A0A286ZWB1	0.391089	7	35	0.0488	Function unknown
HSF_DOMAIN domain-containing protein	A0A4X1UPN4	0.389285	4	8	0.02948	Function unknown
ANK_REP_REGION domain-containing protein	A0A4X1VJ09	0.388617	5	7	0.04452	Function unknown
Ferric oxidoreductase domain-containing protein	A0A4X1UFS1	0.388235	7	21	0.008876	Chemical homeostasis; metal ion binding

FAD-binding PCMH-type domain-containing protein	A0A4X1SXF0	0.388212	9	23	0.03026	Function unknown
Neutral amino acid transporter A isoform 1	I3LAL2	0.38764	2	4	0.04976	Function unknown
Thymidine kinase	A0A286ZID2	0.387416	10	45	0.004381	Function unknown
Uncharacterized protein	F1RZ68	0.386245	11	13	0.009878	Negative regulation of cellular process;
BH3-interacting domain death agonist	Q4JHS0	0.385522	7	56	1.90E-05	Positive regulation of cellular process; ubiquitin-like protein ligase binding
DNA helicase	A0A286ZQR4	0.385254	2	2	0.01025	Function unknown
3'-5' exoribonuclease 1 isoform X1	A0A287AID0	0.384788	2	7	0.001151	Function unknown
Probable tRNA (uracil-O(2)-)-methyltransferase	A0A4X1SU08	0.384379	2	4	0.03331	Function unknown
IF rod domain-containing protein	A0A4X1WBS5	0.38355	2	10	0.02304	Function unknown
Uncharacterized protein	A0A286ZVA8	0.383493	1	60	0.01702	Function unknown
Methionine--tRNA ligase	A0A4X1VG66	0.383384	12	30	0.0102	Function unknown
Uncharacterized protein	A0A4X1SXM2	0.382329	1	8	0.01509	Signal transduction; receptor binding
FAM110_C domain-containing protein	A0A4X1UP72	0.38147	3	10	0.00149	Function unknown
tRNA-splicing endonuclease subunit Sen2	F1SQ91	0.380746	7	20	0.007143	RNA processing; catalytic activity, acting on a tRNA
Rap1 GTPase-activating protein 1 isoform a	I3LMS2	0.380304	5	10	0.007709	Function unknown
Exonuclease domain-containing protein	I3L5D1	0.379946	5	21	0.02673	Function unknown

Polypeptide N-acetylgalactosaminyltransferase	F1RPN0	0.379795	8	21	0.02935	Function unknown
Transmembrane protein 186	A0A287AQT2	0.378524	2	5	0.02112	Function unknown
Dolichyl-diphosphooligosaccharide--protein glycosyltransferase subunit 2	F1SEM2	0.376005	1	52	0.03133	Function unknown
ATPase WRNIP1 isoform 1	F1RWX5	0.375516	3	5	0.01805	Function unknown
Ig-like domain-containing protein	F1RPU6	0.375416	6	22	0.01728	Regulation of biological process;
Ubiquitin-like modifier-activating enzyme 7	A0A480VER4	0.375129	6	8	0.001627	Function unknown
3-hydroxy-3-methylglutaryl coenzyme A synthase	A0A4X1SVG4	0.374889	1	56	0.03264	Organic hydroxy compound metabolic process; catalytic activity
Uncharacterized protein	A0A4X1VEH9	0.374835	2	8	0.03559	Anion transmembrane transporter activity
GCFC domain-containing protein	I3L6B6	0.374464	1	1	0.03676	Function unknown
Uncharacterized protein	A0A4X1TAJ0	0.374419	9	8	0.01385	Function unknown
Uncharacterized protein	A0A4X1VHT0	0.373898	21	33	0.0265	Positive regulation of cellular process; transferase activity, transferring pentosyl groups
Uncharacterized protein	A0A4X1SMZ2	0.373571	6	33	0.004126	Synaptic signaling; binding
Uncharacterized protein	A0A4X1TMJ0	0.372596	1	1	0.00364	Function unknown
Uncharacterized protein	A0A4X1WDI5	0.372385	1	30	0.001976	Function unknown
2-5A-dependent ribonuclease	A0A286ZPJ7	0.371818	19	37	0.01578	MRNA processing; phosphotransferase activity, alcohol group as acceptor
Uncharacterized protein	F1S091	0.371759	3	55	0.00615	Cellular response to organonitrogen compound; small molecule binding
Serine/threonine-protein	A0A4X1UYN5	0.37154	4	30	0.006173	Function unknown

kinase PLK						
SH3 domain-containing protein	I3LS55	0.371356	1	1	0.01494	Function unknown
Coronin	A0A4X1TZD0	0.370795	3	7	0.01909	Function unknown
Uncharacterized protein	A0A4X1TWZ9	0.370606	6	2	0.03308	Function unknown
Uncharacterized protein	A0A4X1SHH8	0.370458	1	2	0.03144	Function unknown
39S ribosomal protein L43, mitochondrial isoform X2	F1S8U4	0.370293	6	35	0.0143	Translation; structural constituent of ribosome
Multifunctional methyltransferase subunit TRM112-like protein	F1RQP1	0.3701	6	54	0.01861	Protein heterodimerization activity
Uncharacterized protein	A0A4X1U855	0.368524	7	17	0.02096	Function unknown
Integrin beta	A0A4X1V025	0.368325	1	47	0.03017	Function unknown
Class E basic helix-loop-helix protein 41	A0A287AYR6	0.36825	2	4	0.02874	Function unknown
Ubiquitinyl hydrolase 1	F1RXF7	0.367771	8	38	0.02913	Thiol-dependent ubiquitin-specific protease activity
Epiplakin isoform X1-like (Fragment)	A0A480PQZ4	0.366945	14	30	0.018	Function unknown
Polynucleotide phosphorylase 1	A0A287AMV5	0.366848	44	67	0.01016	RNA processing; organic cyclic compound binding
Apoptosis-inducing factor 2 isoform X4	A0A480J3T0	0.36667	7	31	0.02832	Function unknown
Uncharacterized protein	A0A4X1SPI0	0.3664	5	10	0.04642	Potassium ion import across plasma membrane; potassium ion transmembrane transporter activity
Pannexin_like domain-containing protein	A0A286ZWM5	0.366106	7	10	0.007317	C4-dicarboxylate transport; channel activity

Phosphatidate cytidylyltransferase, mitochondrial	A0A4X1UGZ8	0.36551	10	36	0.0252	Phosphatidylglycerol metabolic process; cytidylyltransferase activity
Uncharacterized protein	F1SRY9	0.364988	6	20	0.04888	Function unknown
Cytochrome c oxidase assembly factor 6 homolog isoform X1	A0A480IWY5	0.364205	9	7	0.01714	Ribonucleotide binding
Uncharacterized protein	F1SG83	0.363225	9	10	0.004686	Function unknown
MARVEL domain-containing protein	A0A5G2QU86	0.362552	2	28	0.005986	Function unknown
Uridine phosphorylase	A0A480S6M1	0.360586	12	50	0.03899	Function unknown
Protein kinase domain-containing protein	A0A4X1U7M2	0.360327	8	25	0.0258	Ribonucleotide binding
Uncharacterized protein	A0A4X1TB13	0.36009	8	21	0.01712	Protein N-linked glycosylation via asparagine; binding
ADP/ATP translocase 3	A0A287AXS7	0.359841	7	65	0.03464	ADP transmembrane transporter activity
Protein-tyrosine sulfotransferase	A0A480JPS8	0.359432	2	5	0.001727	Sulfur compound metabolic process; binding
[Histone H3]-lysine(4) N-trimethyltransferase	A0A287A913	0.359284	1	0	0.01427	Function unknown
Glycylpeptide N-tetradecanoyltransferase	A0A287B6G4	0.358486	1	13	0.0234	N-terminal peptidyl-glycine N-myristoylation; glycylpeptide N-tetradecanoyltransferase activity
Smad nuclear-interacting protein 1	I3LQU1	0.358454	6	19	0.02953	Function unknown
UDP-glucuronate decarboxylase 1	A0A480TRQ6	0.358189	9	32	0.025	UDP-glucuronate decarboxylase activity
Feline leukemia virus	B7U652	0.358039	3	8	0.003564	Function unknown

subgroup C receptor 1						
TNF receptor-associated factor	A0A4X1SD40	0.357723	7	12	0.01481	Positive regulation of cellular process; phosphatase binding
Uncharacterized protein	A0A287AYH3	0.357008	7	42	0.007064	Function unknown
RHD domain-containing protein	A0A286ZJ10	0.356202	9	27	0.004497	Function unknown
Uncharacterized protein	A0A287BK29	0.355567	3	9	0.01411	Function unknown
Dual specificity protein kinase CLK3 isoform X5	F1SIE6	0.355388	8	18	0.005973	Cellular process; heterocyclic compound binding
Essential for reactive oxygen species protein	A0A5G2QFI7	0.35538	4	15	0.009897	Function unknown
Uncharacterized protein	F1SHQ6	0.354665	4	14	0.0108	Function unknown
F-box only protein 28 isoform a	A0A480TQ31	0.354502	3	9	0.04111	Function unknown
Protein S100-A12	P80310	0.354194	4	57	0.008724	Positive regulation of cellular process; zinc ion binding
Semaphorin-4B isoform 1	A0A287A766	0.353721	7	9	0.0204	Function unknown
Nuclear envelope integral membrane protein 1 isoform a	F1SL61	0.35348	5	10	0.04871	Function unknown
Coatomer subunit delta	A0A287A480	0.35291	1	54	0.007025	Protein transport;
Uncharacterized protein	A0A4X1V9Q2	0.35206	5	25	0.02307	Function unknown
TRUD domain-containing protein	A0A4X1TT57	0.351643	8	13	8.30E-05	Function unknown
Glycoprotein (Fragment)	Q9XSA8	0.351317	1	75	0.03703	Positive regulation of cellular process; mannose binding
Uncharacterized protein	A0A4X1TVW1	0.351287	4	4	0.006243	Function unknown
Protein kinase C	A0A4X1SI40	0.351055	2	5	0.009379	Signal transduction; ribonucleotide binding
NAGPA domain-containing	A0A287AFK0	0.350858	5	13	0.01107	Function unknown

protein						
Sodium/hydrogen exchanger 1	P48762	0.350075	3	5	0.01187	Cation transmembrane transport; active ion transmembrane transporter activity
60S acidic ribosomal protein P2 (Fragment)	A0A480V6V6	0.349333	1	54	0.002102	Translational elongation; structural constituent of ribosome
HECT-type E3 ubiquitin transferase	F1SHF6	0.349112	1	20	0.002418	Protein ubiquitination; ligase activity
Uncharacterized protein	F1S5D4	0.349063	11	18	0.0225	Function unknown
Cytochrome c oxidase copper chaperone	D2XUP7	0.348895	3	59	0.005066	Positive regulation of cellular process; molecular carrier activity
Uncharacterized protein	A0A4X1UHY6	0.348871	2	27	0.03725	Function unknown
Uncharacterized protein	I3LJA8	0.348587	7	7	0.008051	Cell-substrate junction organization;
Arginase	A0A5S8LD81	0.347969	3	13	0.03436	Positive regulation of cellular process; ion binding
SLA-8	Q6S7D1	0.347946	9	41	0.003237	Response to stimulus;
Ubiquitin carboxyl-terminal hydrolase	A0A4X1W4U5	0.347605	9	48	0.00072	Positive regulation of cellular process; peptidase activity, acting on L-amino acid peptides
Uncharacterized protein	A0A287BRT1	0.347528	6	14	0.02	Regulation of biological process; binding
DNAJ homolog subfamily A member 1 isoform 1	F1SE73	0.347295	19	65	0.01441	Positive regulation of cellular process; low-density lipoprotein particle receptor binding
Uncharacterized protein	F1SCM9	0.347243	14	31	0.001509	Cellular component organization or biogenesis;
WD_REPEATS_REGION domain-containing protein	A0A4X1ST95	0.346315	5	14	0.002357	Positive regulation of cellular process; methylated histone binding
Uncharacterized protein	A0A5G2QEV8	0.344341	7	10	0.008928	Function unknown
Uncharacterized protein	A0A4X1UWU7	0.343856	1	1	0.005068	Function unknown
HMG domain-containing protein 4	A0A287B9A3	0.343847	1	3	0.0409	Function unknown
DNA-directed RNA	A0A480YTU7	0.343048	10	15	0.03558	Function unknown

polymerase III subunit RPC5 isoform X1						
t-SNARE coiled-coil homology domain-containing protein	F1RI88	0.343019	8	30	0.01115	Function unknown
Apolipoprotein A2 (Fragment)	Q7YRR7	0.342979	1	53	0.02916	Function unknown
Peptidylprolyl isomerase	A0A287B5T6	0.341704	4	43	0.02346	Peptidyl-prolyl cis-trans isomerase activity
Aurora/IPL1-related kinase 1	A4UTN8	0.340952	6	18	0.03434	Positive regulation of cellular process; small molecule binding
EABR domain-containing protein	A0A287AZV2	0.34076	13	32	0.02132	Anatomical structure development; binding
Uncharacterized protein	A0A287AK23	0.340564	1	1	0.01567	Function unknown
Non-specific serine/threonine protein kinase	A0A286ZTS2	0.339764	1	2	0.04594	Function unknown
Solute carrier family 25 member 33 (Fragment)	A0A480JH88	0.339191	5	17	0.02975	Positive regulation of cellular process; transmembrane transporter activity
Kinesin-like protein KIF11	F1SC89	0.338679	32	36	0.001555	Microtubule-based movement; small molecule binding
Uncharacterized protein	A0A286ZYM2	0.338601	7	30	0.03822	Cation transmembrane transport; macromolecular complex binding
Uncharacterized protein	A0A4X1VV86	0.338169	4	76	0.01653	Negative regulation of cellular process; protein binding
CBFD_NFYB_HMF domain-containing protein	A0A4X1V1W7	0.337311	3	24	0.03406	DNA metabolic process; catalytic activity, acting on DNA
Netrin-1	A0A4X1V6Z8	0.336867	3	8	0.009071	Positive regulation of cellular process;
ATPase H(+)-transporting lysosomal accessory protein 2	A0A5G2QNQ8	0.336662	1	26	0.01751	Function unknown
TMEM189_B_dmain domain-containing protein	A0A4X1UGG4	0.336059	5	25	0.03447	Ether biosynthetic process; plasmanylethanolamine desaturase activity
Uncharacterized protein	F1RIB1	0.333734	3	24	0.01984	Function unknown
Twinkle protein, mitochondrial	I3LNP8	0.333468	4	8	0.00322	Function unknown

isoform A						
C2 domain-containing protein 5 isoform a	A0A480LG90	0.33331	2	3	0.03395	Binding
Proteasome inhibitor PI31 subunit	A0A4X1SLZ8	0.333295	8	41	0.0463	Function unknown
Uncharacterized protein	A0A4X1TG55	0.331991	4	48	0.02449	Positive regulation of cellular process; chromatin binding
NAD-dependent protein deacylase sirtuin-5, mitochondrial	A0A287AF07	0.331798	4	18	0.005802	Negative regulation of cellular process; zinc ion binding
Proteasome subunit beta	A0A4X1V3H8	0.331163	6	28	0.004506	Macromolecule metabolic process; peptidase activity, acting on L-amino acid peptides
Arylamine N-acetyltransferase (Fragment)	C5MLY5	0.330344	1	48	0.03633	Function unknown
DNA polymerase delta subunit 2	A0A4X1UG85	0.330184	9	26	0.005542	DNA biosynthetic process; organic cyclic compound binding
Serine/threonine-protein kinase A-Raf	O19004	0.330035	11	31	0.002364	Regulation of proteolysis; binding
Uncharacterized protein	I3LGI4	0.328383	15	75	0.002551	Function unknown
Uncharacterized protein	I3L8N8	0.328259	3	3	0.02961	Function unknown
Transcription factor 7-like 2 isoform X19	A0A480NGE4	0.327797	1	2	0.02717	Function unknown
Lipoprotein lipase	I3LUA0	0.327353	6	20	0.03753	Primary metabolic process; triglyceride lipase activity
Uncharacterized protein	A0A5G2RLA2	0.326731	2	2	0.004157	Function unknown
Transforming growth factor beta-1 proprotein	P07200	0.324801	9	27	0.04651	Phosphorus metabolic process; receptor binding
B30.2/SPRY domain-containing protein	A0A4X1V4M2	0.324587	8	21	0.02232	Function unknown

Haloacid dehalogenase-like hydrolase domain-containing 5 isoform 2	A0A480JH79	0.324502	12	50	0.02867	Function unknown
RNA polymerase III subunit C3	F1SDD7	0.324311	16	37	0.001239	Function unknown
AAA domain-containing protein	A0A4X1VNQ4	0.324066	1	44	0.0465	Function unknown
Exocyst complex component	A0A480UT78	0.322082	4	8	0.03629	Cellular localization;
Fas apoptosis inhibitory molecule 1	F2YI04	0.321928	2	10	0.004236	Negative regulation of cellular process;
Uncharacterized protein	A0A4X1SLA7	0.321314	5	7	0.04702	Function unknown
Palmitoyl acyl-COA oxidase 1 (Fragment)	A7J0A9	0.320662	1	53	0.01855	Cellular metabolic process; binding
Glycosaminoglycan xylosylkinase	F1S6Y3	0.320054	8	27	0.02554	Phosphorus metabolic process; transferase activity, transferring phosphorus-containing groups
Transmembrane protein 115	F1SJ20	0.320046	8	23	0.02048	Vesicle-mediated transport; binding
SANT domain-containing protein	A0A4X1SJM1	0.31986	3	5	0.03798	Function unknown
Calcium/calmodulin-dependent protein kinase	A0A480Q1T9	0.319682	6	23	0.007103	Function unknown
Mitochondrial tRNA-specific 2-thiouridylase 1	F1SM74	0.31867	8	21	0.008486	Function unknown
N-acylglucosamine 2-epimerase	P17560	0.318449	11	32	0.04328	Organic acid catabolic process; N-acylglucosamine 2-epimerase activity
Interferon alpha/beta receptor 1	A0A4X1TWF4	0.317277	1	3	0.03656	Type I interferon signaling pathway; protein binding
Anaphase-promoting complex	A0A4X1VSN0	0.317071	10	7	0.03966	Function unknown

subunit 1						
IF rod domain-containing protein	A0A4X1WBK2	0.31658	31	76	0.01334	Function unknown
PPM-type phosphatase domain-containing protein	A0A4X1U8I2	0.316027	15	40	0.003217	Phosphorus metabolic process; binding
Ubiquitinyl hydrolase 1	A0A5G2R5Y5	0.314815	3	12	0.02353	Positive regulation of cellular process; peptidase activity, acting on L-amino acid peptides
TRAF-type zinc finger domain-containing protein 1	A0A480LZ70	0.314557	2	7	0.005955	Function unknown
Uncharacterized protein	A0A4X1TZ89	0.314298	2	4	0.01104	Mitotic cell cycle checkpoint;
Hippocalcin-like protein 1	Q06AT0	0.31404	6	34	0.01729	Binding
G protein beta subunit-like	F1RFA8	0.313718	2	5	0.03261	Positive regulation of cellular process; enzyme regulator activity
T-complex protein 1 subunit epsilon	A0A480ZFS4	0.313499	1	74	0.02585	Positive regulation of cellular process; anion binding
Non-receptor tyrosine-protein kinase TNK1 isoform X1	A0A480QM13	0.312517	9	19	0.0106	Function unknown
Tubulin beta chain	A0A4X1VU50	0.311691	5	69	0.009208	Function unknown
Mitochondrial import inner membrane translocase subunit TIM17	A0A4X1VJI6	0.31144	7	51	0.02255	Cellular process; transmembrane transporter activity
Cytochrome P450	A7KZR2	0.311349	2	46	0.004796	Oxidoreductase activity
Secretory carrier-associated membrane protein	A0A4X1VTN9	0.311256	3	14	0.04467	Function unknown
Trimeric intracellular cation channel type B	A0A286ZVK0	0.311213	3	13	0.03284	Negative regulation of cellular process; cation transmembrane transporter activity
Uncharacterized protein	A0A4X1W3E2	0.31091	1	2	0.00557	Function unknown
Large neutral amino acids	K9IVK6	0.31049	8	14	0.004316	Function unknown

transporter small subunit 1						
Erf4 domain-containing protein	A0A5G2QGA7	0.31049	5	39	0.01843	Vesicle-mediated transport to the plasma membrane;
Lysosome membrane protein 2	F1RYT3	0.310236	10	25	0.01026	Protein transport; quaternary ammonium group binding
High mobility group AT-hook 1 transcript variant 2	B6CVL5	0.309805	1	51	0.03036	Function unknown
Uncharacterized protein	F1SG89	0.309721	3	3	0.02448	Function unknown
Uncharacterized protein	A0A287BS07	0.309344	10	37	0.01814	Function unknown
Uncharacterized protein	A0A4X1TN62	0.308466	7	24	0.01658	Function unknown
Integrin alpha-5	A0A480X7T0	0.307242	16	21	0.003666	Positive regulation of cellular process; integrin binding
Uncharacterized protein	A0A4X1VM68	0.307029	1	16	0.04126	Function unknown
Mothers against decapentaplegic homolog	A0A286ZNF5	0.306219	3	8	0.00413	Positive regulation of cellular process; transcription factor activity, sequence-specific DNA binding
Unconventional myosin-Ie	A0A287BEA3	0.303296	26	28	0.003582	Ribonucleotide binding
Aldehyde dehydrogenase	A0A480KA98	0.302723	1	24	0.006033	Function unknown
Uncharacterized protein	A0A4X1TJ15	0.302707	1	3	0.003242	Function unknown
Major facilitator superfamily domain-containing protein 6	A1DWM3	0.302297	3	7	0.007263	Function unknown
Formyltetrahydrofolate synthetase	A0A480VMB4	0.302203	2	47	0.005589	Function unknown
Phosphate transporter	A0A4X1U8Y8	0.301195	5	11	0.01188	Function unknown
Radical S-adenosyl methionine domain-containing protein	A0A5G2R1S6	0.300949	1	2	0.00058	Aromatic compound biosynthetic process; iron-sulfur cluster binding
Semaphorin-3C	A0A4X1SIW8	0.300829	16	27	0.002753	Function unknown
Natural resistance-associated	A0A4X1WCA8	0.3002	3	5	0.02671	Metal ion transmembrane transporter activity

macrophage protein 1						
Uncharacterized protein	A0A287B470	0.29986	6	9	0.02888	Function unknown
Protein C-ets-1 isoform 4	A0A480XB33	0.299502	3	14	0.02453	Function unknown
RNA helicase	A6M928	0.299287	14	75	0.001266	Translational initiation; translation initiation factor activity
Uncharacterized protein	A0A4X1UJN1	0.298791	4	14	0.02141	Function unknown
Transforming acidic coiled-coil-containing protein 3	F1S8S7	0.298761	8	16	0.01376	Cellular component organization or biogenesis;
Cleft lip and palate transmembrane protein 1-like protein	A0A4X1TLU2	0.298417	1	37	0.005909	Function unknown
CKAP2_C domain-containing protein	A0A4X1SQC7	0.298363	17	31	0.007493	Function unknown
Heat shock 27 kDa protein	A0A5S6G3Y8	0.297926	10	28	0.00086	Regulation of protein phosphorylation; protein binding
Uncharacterized protein	A0A287BNX8	0.297593	9	13	0.02438	Function unknown
Uncharacterized protein	A0A4X1TTA3	0.297198	4	4	0.0123	Function unknown
Uncharacterized protein	A0A286ZMQ7	0.296909	8	26	0.01519	Function unknown
RING-type domain-containing protein	A0A287A6L6	0.296899	13	34	0.006895	Positive regulation of cellular process; peptidase inhibitor activity
Uncharacterized protein	A0A4X1STR1	0.296832	1	1	0.003541	DNA metabolic process; tyrosyl-DNA phosphodiesterase activity
40S ribosomal protein S23 (Fragment)	Q29298	0.296738	1	23	0.03345	Function unknown
Importin N-terminal domain-containing protein	A0A4X1U4Q1	0.296325	5	16	0.004003	Cellular process; GTPase binding
Protein NEDD1 isoform a	A0A480SZT8	0.295783	3	7	0.03672	Function unknown
Protein kinase domain-containing protein	A0A5G2RHW3	0.295468	10	44	0.006294	Function unknown

Aa_trans domain-containing protein	A0A4X1TLM5	0.294733	5	10	0.02129	Function unknown
ADP-ribosylation factor-related protein 1 isoform X1	A0A287AP37	0.294482	8	55	0.005971	GTP binding
Cytoskeleton-associated protein 4	A0A480YZR2	0.293807	33	64	0.01406	Function unknown
ER membrane protein complex subunit 1	A0A5G2QQ04	0.293111	1	47	0.0332	Function unknown
Peroxin-14	A0A4X1W7K3	0.293051	8	23	0.006595	Function unknown
Uncharacterized protein	A0A4X1VZD8	0.292286	20	64	0.015	Function unknown
Methyl-CpG-binding protein 2	A0A5G2QL75	0.291948	10	27	0.02958	Function unknown
Integrin beta	A0A480QHL7	0.291561	2	48	0.000325	Function unknown
Ubiquitin-like modifier-activating enzyme ATG7	D7RA25	0.291381	8	16	0.006586	Positive regulation of cellular process; ligase activity, forming carbon-sulfur bonds
Poly(ADP-ribose) glycohydrolase	A0A4X1VEI7	0.290901	8	10	0.02957	Function unknown
SAM_MT_RSMB_NOP domain-containing protein	A0A4X1UJD8	0.290825	6	22	0.03407	Methylation; binding
Uncharacterized protein	A0A4X1UQ65	0.290505	4	28	0.02349	Positive regulation of cellular process; sugar-phosphatase activity
Calcium channel flower homolog isoform a	A0A286ZPA5	0.290348	1	5	0.04185	Function unknown
Torsin-1B isoform 1 (Fragment)	A0A481DX94	0.290196	3	14	0.02696	Function unknown
N6-adenosine-methyltransferase catalytic subunit	F1S8J8	0.290161	10	24	0.02974	MRNA processing; mRNA (2'-O-methyladenosine-N6-

Uncharacterized protein	A0A4X1W7C1	0.289805	8	30	0.003703	Function unknown
Uncharacterized protein	F1SJ93	0.289607	2	47	0.02474	Binding
Uncharacterized protein	A0A4X1T3F8	0.289594	10	50	0.01262	Negative regulation of cellular process; protein binding
Protein tweety homolog	A0A4X1TMB9	0.289535	3	5	0.03644	Function unknown
Uncharacterized protein	A0A5G2R2D5	0.289521	18	40	0.004011	Function unknown
Uncharacterized protein	F1S6F1	0.28937	2	6	0.02247	Function unknown
Transmembrane channel-like protein	A0A4X1UTJ4	0.289037	4	7	0.04297	Function unknown
Protein kinase domain-containing protein	A0A4X1SUE0	0.28841	8	18	0.01718	Function unknown
L-serine ammonia-lyase	A0A287ASY9	0.287988	1	5	0.03602	Function unknown
DNA-directed RNA polymerase	M3V845	0.287843	15	16	0.01598	Function unknown
60S ribosomal protein L21	P49666	0.287789	12	52	0.002775	Translation; RNA binding
Serine/threonine-protein kinase TBK1	A0A480NCW3	0.287513	10	17	0.005557	Positive regulation of cellular process; small molecule binding
Charged multivesicular body protein 4a (Fragment)	A0A480WNJ0	0.287163	4	17	0.02253	Function unknown
Uncharacterized protein	A0A4X1UD59	0.287049	15	34	0.01991	RNA binding
RNA-binding protein PNO1	A0A287B6J3	0.286904	8	49	0.02837	Binding
Uncharacterized protein	A0A287BPL8	0.28685	18	21	0.006515	Cell-cell adhesion via plasma-membrane adhesion molecules; calcium ion binding
Uncharacterized protein	A0A5G2QE37	0.286616	1	2	0.018	Muscle tissue development; isomerase activity
Ribonucleoside-diphosphate reductase	A0A287AJ98	0.28506	13	43	0.003843	Function unknown
Phospholipid scramblase	A0A5G2QX76	0.284918	5	18	0.02383	Function unknown
Ig-like domain-containing	A0A4X1VVJ0	0.283234	3	47	0.005706	Biological adhesion;

protein						
Uncharacterized protein	A0A4X1TYZ6	0.28294	4	15	0.03366	Function unknown
IST1 homolog	A0A287A426	0.282689	15	57	0.00458	Function unknown
Serine/threonine-protein kinase Nek6	A0A5S6G0N1	0.282603	2	9	0.02898	Ribonucleotide binding
Uncharacterized protein	A0A4X1VV82	0.282522	17	18	0.009253	Function unknown
PIN2/TERF1-interacting telomerase inhibitor 1 isoform 1	A0A287AHL8	0.282099	6	27	0.02847	Function unknown
Zyxin	F1SRV9	0.281514	1	42	0.002164	Ion binding
Cytochrome c oxidase subunit	A0A287BGN0	0.281204	2	43	0.01687	Hydrogen ion transmembrane transporter activity
SpoU_sub_bind domain-containing protein	A0A5G2R9B5	0.280591	4	15	0.01278	RNA processing; RNA binding
NADH dehydrogenase [ubiquinone] 1 alpha subcomplex assembly factor 3	F1SKI6	0.27978	5	45	0.0222	Function unknown
PH domain-containing protein	A0A4X1W5J1	0.279376	32	34	0.001466	Function unknown
Cyclin M4 (Fragment)	K9IWA2	0.278089	7	12	0.004061	Function unknown
Membrane cofactor protein	A0A4X1TD61	0.277373	9	39	0.01213	Function unknown
ADP-ribosylation factor-like protein 6-interacting protein 4	A0A287AL31	0.277228	3	18	0.02418	Function unknown
Uncharacterized protein	A0A4X1T4W4	0.276835	26	13	0.02143	Function unknown
GTP-binding protein 1	A0A4X1VLR6	0.276833	6	15	0.004588	Positive regulation of cellular process; ribonucleotide binding
Charged multivesicular body protein 2a	A0A481CY36	0.27662	6	17	0.005283	Function unknown
Uncharacterized protein	F1RF76	0.276212	14	24	0.04582	Negative regulation of cellular process; purine ribonucleotide binding

Non-specific serine/threonine protein kinase	A0A480JSC9	0.276105	17	15	0.02387	Function unknown
4HBT domain-containing protein	A0A4X1VNM5	0.275689	4	26	0.01655	Function unknown
J domain-containing protein	A0A5G2QJP3	0.275662	8	22	0.02282	Function unknown
GPI-anchor transamidase	A0A480JKQ6	0.275357	8	34	0.009032	Attachment of GPI anchor to protein; peptidase activity, acting on L-amino acid peptides
RanBP-type and C3HC4-type zinc finger-containing protein 1	F1S7J3	0.275243	2	6	0.0125	Function unknown
Chloride channel protein	A0A4X1SK26	0.275139	3	5	0.04122	Function unknown
Leukocyte elastase inhibitor	M3TYG7	0.274917	12	34	0.002002	Negative regulation of cellular process; serine-type endopeptidase inhibitor activity
FABP domain-containing protein	F6PVY0	0.274716	5	21	0.005333	Binding
Uncharacterized protein	A0A4X1V080	0.27399	3	6	0.02072	Positive regulation of cellular component biogenesis;
Uncharacterized protein	A0A5G2Q9F2	0.273844	16	21	0.01372	Function unknown
Dolichyl-phosphate-mannose--protein mannosyltransferase	A0A481BKM6	0.273248	3	7	0.0418	Function unknown
Macro domain-containing protein	A0A4X1SU91	0.273227	3	7	0.03794	Function unknown
RISC-loading complex subunit TARBP2 isoform a	A0A480W1S2	0.273173	4	13	0.03236	Positive regulation of cellular process; siRNA binding
DBB domain-containing protein	A0A4X1UEY2	0.273007	25	39	0.001563	Function unknown
Sulfurtransferase	A0A287BPT5	0.272857	7	31	0.0129	Function unknown
RING-CH-type domain-containing protein	A0A4X1V023	0.271871	10	44	0.01104	Positive regulation of cellular process;
Beta-parvin (Fragment)	A0A480MYU1	0.271476	7	29	0.003278	Function unknown

PH domain-containing protein	A0A287BEJ3	0.270749	26	44	0.01116	Function unknown
Uncharacterized protein	F1STI9	0.27066	13	34	0.03268	Binding
V-type proton ATPase subunit a	A0A4X1STL1	0.270438	13	21	0.03479	Function unknown
Solute carrier family 30 member 5	B6ECZ1	0.270395	6	10	0.004122	Function unknown
Transmembrane protein 201 isoform 1	A0A480NUA5	0.270323	9	17	0.01464	Nuclear migration; binding
Formin-like protein 2 isoform X5	A0A480RHY5	0.269951	14	19	0.01386	Function unknown
Ribonucleases P/MRP protein subunit POP1	F1S0M3	0.269949	34	42	0.01089	RNA processing; catalytic activity, acting on a tRNA
Protein-S-isoprenylcysteine O-methyltransferase	A0A481C9N4	0.269679	3	18	0.01732	Methyltransferase activity
BTB/POZ domain-containing adapter for CUL3-mediated RhoA degradation protein 2	I3LM60	0.269621	3	16	0.00055	Negative regulation of cellular process; catalytic activity, acting on a protein
Mitochondrial import inner membrane translocase subunit Tim10 B	A0A5G2R1A5	0.269582	3	48	0.04843	Protein transport; metal ion binding
Uncharacterized protein	A0A4X1VJF1	0.269478	22	31	0.03647	MRNA processing;
Caspase-10 isoform 1 preproprotein	A0A286ZWN2	0.268833	10	26	0.007925	Regulation of biological process; peptidase activity
Uncharacterized protein	A0A286ZQP1	0.268673	21	29	0.009253	Function unknown
Uncharacterized protein	A0A4X1U6S6	0.268401	8	46	0.03467	Function unknown
Uncharacterized protein	A0A4X1SJU0	0.268233	6	21	0.01714	Function unknown

WD_REPEATS_REGION domain-containing protein	A0A4X1UEH1	0.26814	3	4	0.02654	Regulation of biological process;
BcL10	A0A4P8ETX9	0.268048	4	16	0.001104	Positive regulation of cellular process; protein binding
Methenyltetrahydrofolate cyclohydrolase	F1SLH0	0.267124	15	57	0.003007	Function unknown
Bromodomain-containing protein 9 isoform 1	A0A480TXN7	0.267025	2	9	0.0106	Function unknown
Uncharacterized protein	A0A5G2QMR2	0.266626	5	16	0.003521	Function unknown
Uncharacterized protein	A0A287BPU8	0.266336	6	8	0.006906	Function unknown
Zyxin	A0A481AUZ6	0.266226	1	42	0.02571	Ion binding
Uncharacterized protein	A0A5G2R4I4	0.26601	1	4	0.0121	Function unknown
Uncharacterized protein	A0A4X1U8D6	0.264936	5	17	0.04284	Function unknown
ATP-binding cassette, sub-family C (CFTR/MRP), member 13	K9J4N7	0.264796	20	22	0.00938	Function unknown
N-myc downstream-regulated gene 3 protein	F1S479	0.264245	8	28	0.002158	Function unknown
Phosphatidate phosphatase	D2I970	0.26381	6	10	0.005328	Function unknown
Uncharacterized protein	A0A4X1U7W6	0.263763	11	42	0.02621	Intracellular transport;
**Downregulated proteins in PK-15-infected cells (a total of 263 proteins)**						
Uncharacterized protein	A0A4X1VPY8	-1.73844	1	0	0.0311	Function unknown
Nucleolar and coiled-body phosphoprotein 1 isoform 3	A0A480HP04	-1.075915	2	32	0.004571	Function unknown
Costars domain-containing protein	A0A4X1VHY8	-1.070759	2	47	0.02314	Regulation of cellular process;
Tropomyosin alpha-1 chain	A0A4X1TT60	-1.02607	1	48	0.001151	Function unknown

Succinate dehydrogenase cytochrome b560 subunit, mitochondrial	D0VWV4	-1.023671	2	12	0.005618	Cellular metabolic process; binding
FXYD domain-containing ion transport regulator	Q58K79	-0.946206	2	42	0.02542	Potassium ion import across plasma membrane; sodium channel regulator activity
Glucose-6-phosphate 1-dehydrogenase	A0A0B8RVW4	-0.881936	2	68	0.02565	Function unknown
Olfactory receptor	A0A287AX70	-0.879124	1	2	0.02989	Signal transduction; olfactory receptor activity
Na(+)/H(+) exchange regulatory cofactor NHE-RF	A0A481BZK7	-0.85337	1	56	0.01396	Binding, bridging
Uncharacterized protein	A0A5G2QSQ7	-0.827559	1	30	0.008274	Response to stimulus; protein binding
Heterogeneous nuclear ribonucleoprotein K	A0A287A699	-0.795753	1	60	0.001239	Positive regulation of cellular process; transcriptional activator activity, RNA polymerase II transcription regulatory region sequence-specific binding
Syndecan	F1S0L5	-0.793107	5	21	0.003058	Function unknown
Low-density lipoprotein receptor-related protein 2	C0HL13	-0.778382	2	24	0.01125	Function unknown
Protein jagunal homolog 1	A0A480ZDX8	-0.744131	1	7	0.004547	Function unknown
DEP domain-containing protein	A0A4X1UQ57	-0.733291	1	1	0.00978	System process;
Uncharacterized protein	A0A4X1TC08	-0.723131	2	43	0.0103	Ruffle assembly;
39S ribosomal protein L51, mitochondrial	A0A4X1UUV6	-0.718027	2	22	0.006842	Function unknown
ATP synthase membrane subunit f	A0A4X1U911	-0.715818	2	16	0.02133	Function unknown
Uncharacterized protein	A0A287BLS0	-0.707426	2	29	0.003809	Positive regulation of cellular process; organic cyclic compound binding
Uncharacterized protein	A0A4X1UEL7	-0.698497	2	8	0.003356	Function unknown

Solute carrier family 3 member 1	B2D2K8	-0.676169	10	17	0.002449	Function unknown
Acylphosphatase	J9JIK5	-0.664148	2	12	0.02357	Acylphosphatase activity
Methyltransferase like 7A	A0A0B8RSL6	-0.650465	1	26	0.00421	Function unknown
Mab-21 domain-containing protein	A0A4X1TXP6	-0.64769	1	3	0.000456	Positive regulation of cellular process; small molecule binding
Uncharacterized protein	A0A5G2R3Z4	-0.628623	30	53	0.04522	Function unknown
DNA-directed RNA polymerases I, II, and III subunit RPABC5	A0A4X1VYD0	-0.616299	2	30	0.001167	Aromatic compound biosynthetic process; zinc ion binding
Uncharacterized protein	A0A5G2QST4	-0.615868	1	27	0.03032	Function unknown
ATP synthase subunit a	Q1HBH9	-0.61512	2	10	0.003376	Cation transmembrane transport; cation transmembrane transporter activity
Uncharacterized protein	A0A4X1VD17	-0.599825	17	44	0.04624	Nucleoside phosphate metabolic process; fatty-acyl-COA synthase activity
Uncharacterized protein	A0A4X1T382	-0.599098	1	34	0.000937	Positive regulation of cellular component biogenesis; binding
Uncharacterized protein	A0A4X1W2Y2	-0.590491	15	32	0.005908	Function unknown
Uncharacterized protein	A0A287APU9	-0.590039	3	5	0.02762	Function unknown
Na(+)/H(+) exchange regulatory cofactor NHE-RF	B6EAV5	-0.586255	1	56	0.03215	Binding, bridging
60S acidic ribosomal protein P1	A0A4X1T568	-0.582802	4	96	0.008206	Translation; enzyme regulator activity
Sickle tail protein homolog isoform 1	A0A480S813	-0.5752	3	28	0.03532	Function unknown
39S ribosomal protein L33, mitochondrial	A0A4X1TLP9	-0.574189	3	49	0.01714	Translation; structural constituent of ribosome
Envelope protein	Q6W4V3	-0.571872	4	38	0.00603	Function unknown
Dystrobrevin	A0A480U1E0	-0.571399	1	11	0.02634	Function unknown

ATP synthase protein 8	Q71K49	-0.561017	2	39	0.001069	Cation transmembrane transport; transmembrane transporter activity
Uncharacterized protein	A0A4X1V3D9	-0.556866	6	53	0.04805	Function unknown
Alpha-1-microglobulin	A0A480P2R0	-0.554327	2	7	0.004685	Protein metabolic process; protein dimerization activity
VWFA domain-containing protein	A0A5G2RG33	-0.553539	4	3	0.01385	Function unknown
LITAF domain-containing protein	A0A4X1VJC1	-0.55313	1	4	0.01371	Regulation of NIK/NF-kappab signaling; RNA polymerase II core promoter proximal region sequence-specific DNA binding
Uncharacterized protein	A0A5G2QVV0	-0.543857	1	68	0.03396	Function unknown
Mitochondrial NDUFA4	A1XQS3	-0.541115	6	56	0.002355	Function unknown
Protein cordon-bleu isoform b	A0A480RTP6	-0.535727	7	12	0.02446	Function unknown
Phosphoinositide phospholipase C	A0A4X1SQG1	-0.52998	1	0	0.000466	Negative regulation of cellular process; calcium-dependent phospholipase C activity
Uncharacterized protein	A0A5G2QGQ5	-0.522642	1	41	0.03352	Function unknown
SUI1 domain-containing protein	A0A5G2QLD8	-0.520002	3	78	0.01101	Regulation of translation; translation initiation factor activity
NADH-ubiquinone oxidoreductase chain 3	B5KN66	-0.51547	1	13	0.001701	Oxidoreductase activity
UDP-glucuronosyltransferase	F1SM28	-0.514762	11	51	0.04233	Function unknown
Uncharacterized protein	A0A4X1TPF2	-0.50651	6	14	0.02736	Function unknown
Methyltransf_11 domain-containing protein	A0A4X1W6X4	-0.505324	1	26	0.001346	Function unknown
Complex I-MNLL	F1SD73	-0.501815	2	34	0.001911	Function unknown
Threonyl-tRNA synthetase	A0A480F5A7	-0.501167	1	53	0.009859	Function unknown
Complex I-B17	A0A480KM09	-0.495333	4	20	0.007112	Mitochondrial electron transport, NADH to ubiquinone;
Mannose-P-dolichol utilization defect 1 protein	Q1EG61	-0.490797	3	15	0.000828	Carbohydrate biosynthetic process;

Uncharacterized protein	A0A287B8I3	-0.486289	5	18	0.03733	Function unknown
WD_REPEATS_REGION domain-containing protein	A0A5K1UE68	-0.479411	4	6	0.004916	Function unknown
Microsomal glutathione S-transferase 3	Q2EN77	-0.476284	3	25	0.04562	Leukotriene biosynthetic process; glutathione peroxidase activity
SOSS complex subunit C	A0A480MDH4	-0.47363	2	23	0.006029	DNA metabolic process;
Uncharacterized protein	A0A4X1V1E4	-0.469897	3	17	0.000469	Generation of precursor metabolites and energy;
Caldesmon isoform X1	A0A480TAI8	-0.467487	19	40	0.03452	System process; calmodulin binding
Gag protein	R4JTP7	-0.466897	2	24	0.002376	Symbiosis, encompassing mutualism through parasitism; binding
Peptidylprolyl isomerase	I3LR51	-0.465877	11	43	0.01648	Isomerase activity
Uncharacterized protein	A0A5G2QTG5	-0.464974	5	50	0.007785	Regulation of biological process; disulfide oxidoreductase activity
60S ribosomal protein L11	A0A4X1VYM5	-0.464038	1	40	0.008529	Positive regulation of cellular process; anion binding
Protein-tyrosine-phosphatase	A0A480JYK3	-0.461254	2	2	0.04534	Phosphoprotein phosphatase activity
Leucyl-tRNA synthetase	A0A480J1H2	-0.460836	2	55	0.0185	Function unknown
3 beta-hydroxysteroid dehydrogenase/Delta 5-->4-isomerase	Q9N119	-0.458885	3	10	0.004437	Response to steroid hormone; steroid delta-isomerase activity
Uncharacterized protein	A0A286ZQ23	-0.457321	12	32	0.01621	Catalytic activity
Small nuclear ribonucleoprotein Sm D3	A0A4X1V3C3	-0.457133	4	48	0.03059	Protein binding
VPS10 domain-containing protein	A0A287A1U0	-0.455375	11	13	0.03193	Function unknown
PKD domain-containing protein	A0A286ZJF6	-0.453646	7	13	0.007594	Function unknown
6-pyruvoyl tetrahydrobiopterin synthase	A0A4X1TBY4	-0.450699	6	46	0.0441	Organic hydroxy compound metabolic process; protein binding

Uncharacterized protein	I3LNG8	-0.448904	1	65	0.04414	Response to organic substance; binding
HAUS augmin-like complex subunit 8 isoform b	A0A287AK92	-0.444763	2	5	0.04419	Function unknown
Cold-inducible RNA-binding protein	A0A4X1VUM6	-0.44282	4	24	0.01364	RNA binding
Anaphase-promoting complex subunit 4	I3LFB6	-0.442734	3	5	0.002608	Function unknown
Protein archease	A0A4X1VY44	-0.437222	2	16	0.03707	Function unknown
Uncharacterized protein	A0A4X1UGK0	-0.436728	6	14	0.00111	Function unknown
Cytochrome c oxidase subunit 1	A0A097P5T5	-0.435674	2	7	0.01063	Cation transmembrane transport; transmembrane transporter activity
Uncharacterized protein	A0A4X1THY8	-0.434969	2	8	0.01766	Negative regulation of cellular process; glutathione binding
Uncharacterized protein	A0A5K1VSX1	-0.432688	1	41	0.02495	Function unknown
Inositol polyphosphate 1-phosphatase isoform X1	F1SN51	-0.431851	11	35	0.03587	Small molecule metabolic process; inositol phosphate phosphatase activity
Uncharacterized protein	A0A4X1TNF0	-0.430897	6	80	0.04255	Function unknown
Sorting nexin-3	A0A5G2R4Z1	-0.428617	5	56	0.01151	Positive regulation of neuron projection development; protein binding
Glutamyl-tRNA(Gln) amidotransferase subunit C, mitochondrial	A0A4X1T4U0	-0.422173	5	51	0.004879	Small molecule metabolic process; ribonucleotide binding
4a-hydroxytetrahydrobiopterin dehydratase	A0A4X1TCL6	-0.420495	3	37	0.02287	Positive regulation of cellular process; phenylalanine 4-monooxygenase activity
CARD domain-containing protein	A0A4X1UPG3	-0.413925	4	32	0.03238	Regulation of biological process;
Uncharacterized protein	A0A4X1VHN4	-0.412898	2	3	0.001515	Function unknown
Armadillo repeat-containing	I3LLX9	-0.411838	8	47	0.001317	Function unknown

protein 10 isoform b						
Low molecular weight cytosolic acid phosphatase	A0A4X1UMF6	-0.408275	3	65	0.01346	Hydrolase activity, acting on ester bonds
Dynamin-like 120 kDa protein, form S1	F1SFG7	-0.405501	1	52	0.004694	Negative regulation of cellular process; pyrophosphatase activity
Neuferricin isoform 1	A0A287B291	-0.401838	4	17	0.01051	Positive regulation of cellular process; tetrapyrrole binding
Galectin	A0A5G2R7M4	-0.39914	10	40	0.007919	Binding
APC-binding protein EB1	F6Q3L0	-0.397089	12	57	0.03179	Positive regulation of cellular component biogenesis; tubulin binding
NADH dehydrogenase [ubiquinone] 1 beta subcomplex subunit 8, mitochondrial	A0A480QR88	-0.395266	7	53	0.003135	Mitochondrial electron transport, NADH to ubiquinone; oxidoreductase activity, acting on NAD(P
MFS domain-containing protein	A0A4X1VJW9	-0.395207	1	2	0.03333	Function unknown
Uncharacterized protein	A0A5G2RKU4	-0.393543	1	4	0.002038	Function unknown
Cleavage and polyadenylation specificity factor subunit 5	A0A5G2QJ53	-0.39266	12	53	0.001959	Positive regulation of cellular process; mRNA 3'-UTR binding
Keratinocyte-associated transmembrane protein 2	A0A287B7K2	-0.392271	2	9	0.000871	Function unknown
Protein S100 (Fragment)	A0A480ZBF9	-0.389632	5	29	0.04552	Positive regulation of cellular process; receptor binding
Serine protease HTRA1	A0A480SWE4	-0.389112	6	19	0.01697	Function unknown
Hepatocyte nuclear factor 1-beta	A0A4X1T4P3	-0.387548	1	24	0.02617	Function unknown
Moesin	A0A4X1VJV5	-0.387237	28	58	0.03177	Positive regulation of cellular process; receptor binding
Beta-lactamase-like protein 2	F1RU12	-0.386954	14	59	0.01864	Zinc ion binding
Uncharacterized protein	A0A4X1UK48	-0.383815	10	19	0.01835	RNA binding

Uncharacterized protein	A0A5G2RCE3	-0.382638	46	27	0.001959	Inositol 1,4,5 trisphosphate binding
U1 small nuclear ribonucleoprotein C	A0A287BG27	-0.382273	3	25	0.002356	mRNA splice site selection; zinc ion binding
NADH dehydrogenase [ubiquinone] 1 alpha subcomplex subunit 6	A0A480TSQ7	-0.37908	7	59	0.002628	Metabolic process;
Tyrosine-protein kinase	A0A0B8RVQ8	-0.378626	18	52	0.001386	Function unknown
Hepatoma-derived growth factor-related protein 2	A0A0B8RTM7	-0.376639	13	19	0.01555	Function unknown
LIM zinc-binding domain-containing protein	A0A4X1TWY1	-0.375821	3	31	0.03765	Signal transduction; heterocyclic compound binding
Uncharacterized protein	A0A286ZLW6	-0.375311	1	44	0.04	Function unknown
60S ribosomal protein L13a	A0A4X1VYE4	-0.374779	11	45	0.01564	Negative regulation of cellular process; structural constituent of ribosome
Low-density lipoprotein receptor relative with 11 ligand-binding repeats	I3L8K1	-0.373727	27	17	0.02035	Function unknown
Activated RNA polymerase II transcriptional Coactivator p15	A0A287AUW4	-0.372939	8	61	0.01696	Positive regulation of cellular process; catalytic activity, acting on DNA
Histone-lysine N-methyltransferase SETD7	I3LI15	-0.372514	5	16	0.04309	Cellular metabolic process; catalytic activity, acting on a protein
Uncharacterized protein	A0A4X1UJN2	-0.372104	7	16	0.005373	Function unknown
Uncharacterized protein	A0A287A3A3	-0.371506	11	26	0.02334	Function unknown
AMP-binding domain-containing protein	A0A287B8R8	-0.369988	27	51	0.000338	Function unknown
Complex I-15 kDa	A0A4X1VX77	-0.368641	7	64	0.001678	Function unknown
Paired domain-containing	A0A4X1UJ76	-0.368016	8	24	0.0083	Function unknown

protein						
Rab11 family-interacting protein 2	A0A287BJL3	-0.366828	1	5	0.0469	Signal transduction; protein dimerization activity
Ubiquinol-cytochrome c reductase complex	Q2EN79	-0.365493	4	63	0.001805	Respiratory chain complex III assembly;
Uncharacterized protein	A0A286ZRK0	-0.3645	1	48	0.04036	Function unknown
Uncharacterized protein	I3LJU0	-0.363339	5	7	0.03454	Function unknown
Uncharacterized protein	A0A5G2R8F7	-0.363134	7	52	0.03574	Positive regulation of cellular process; metal ion binding
Uncharacterized protein	A0A287AUY1	-0.361456	1	7	0.04789	Positive regulation of cellular process; proline-rich region binding
Alpha-galactosidase	A0A4X1W6B2	-0.35897	16	47	0.009742	Metabolic process;
Uncharacterized protein	A0A4X1VGM0	-0.35891	2	11	0.007724	Function unknown
Heterogeneous nuclear ribonucleoprotein D0 isoform c	A0A480M1B4	-0.358755	15	50	0.004162	RNA binding
Ribonuclease P protein subunit p25-like protein	F1SEB9	-0.358183	3	25	0.01125	Binding
Inhibitor of nuclear factor kappa-B kinase subunit alpha	A0A4X1U537	-0.356545	1	2	0.04751	Ribonucleotide binding
Enhancer of rudimentary homolog	A0A4X1TZH0	-0.355545	5	31	0.002802	Cell cycle;
Sorbin and SH3 domain-containing protein 2	A0A4X1VW01	-0.354712	4	26	0.03358	Function unknown
Cyclin-dependent kinase 2-associated protein	F1RFL1	-0.353623	2	20	0.02467	Positive regulation of cellular process; protein binding
Uncharacterized protein	F1SES5	-0.349758	14	28	0.01374	Function unknown
RNA helicase	I3LB32	-0.349354	6	13	0.002823	Ribonucleotide binding
UDPGT domain-containing	A0A4X1V679	-0.346963	10	19	0.001739	Transferase activity, transferring hexosyl groups

protein						
AT-rich interactive domain-containing protein 5B	A0A5G2R8R9	-0.346144	2	2	0.01695	Function unknown
Ensconsin isoform 3	A0A480SUG5	-0.346053	12	19	0.006664	Function unknown
KOW domain-containing protein	A0A4X1V977	-0.345615	2	35	0.001278	Positive regulation of cellular process; ion binding
NAD kinase 2, mitochondrial	A0A287BBX3	-0.345564	1	42	0.004743	Nicotinamide nucleotide metabolic process; ribonucleotide binding
Uncharacterized protein	A0A5G2RAN0	-0.34496	8	22	0.01272	Positive regulation of cellular process;
Uncharacterized protein	A0A4X1T835	-0.343661	4	3	0.01532	Function unknown
Aldehyde dehydrogenase	I6L6E1	-0.343562	1	21	0.01918	Function unknown
Uncharacterized protein	A0A4X1TWS7	-0.342364	14	68	0.003919	Positive regulation of cellular process; protein binding
Stearoyl-COA 9-desaturase	F6Q284	-0.342252	9	24	0.03004	Function unknown
Septin 10	A0A173G6G7	-0.341978	12	40	0.02112	GTP binding
Protein GNAS isoform GNASS	A0A480UPA5	-0.341721	5	43	0.02086	Positive regulation of cellular process; pyrophosphatase activity
Fes1 domain-containing protein	F1RGI9	-0.341664	7	20	0.00463	Function unknown
Tetraspanin-13	F1SEI8	-0.339873	3	21	0.01147	Function unknown
Uncharacterized protein	A0A286ZXF7	-0.339327	1	50	0.01629	Function unknown
Plexin-B2 isoform X2	A0A287BLW6	-0.338751	2	48	0.0267	Function unknown
Calponin	A0A4X1VU33	-0.335713	11	55	0.001207	Function unknown
Cyclin-dependent kinases regulatory subunit	A0A4X1W0Z6	-0.335331	2	33	0.03362	Positive regulation of cellular process; enzyme regulator activity
AP complex subunit sigma	A0A5G2QYA7	-0.334773	6	42	0.007825	Vesicle-mediated transport; clathrin adaptor activity
Complex I-9kD	A0A4X1TFI3	-0.334553	1	37	0.002332	Function unknown
Cathepsin B	B2CNZ7	-0.334198	10	41	0.02029	Decidualization; catalytic activity, acting on a protein
SWI/SNF-related matrix-	F1RXE6	-0.333233	16	36	0.005748	Negative regulation of cellular process; receptor binding

associated actin-dependent regulator of chromatin subfamily E member 1						
Protein kish-B	A0A4X1TF33	-0.331898	2	10	0.03749	Secretion;
