# Supplementary material for: Comparative Proteomic Analysis Reveals Mx1 Inhibits Senecavirus A Replication in PK-15 Cells by Interacting with the Capsid Proteins VP1, VP2 and VP3
Source: Viruses. 2022 Apr 21;14(5):863. doi: 10.3390/v14050863 (PMC9147370; doi:10.3390/v14050863)
Supplement: Supplementary file 1 [file viruses-14-00863-s001.zip › Supplementary Table S3.pdf]

Supplementary Table S3 The differentially expressed proteins identified by TMT labeling coupled with LC-MS/MS analysis of PK-15 cells in response to SVA infection at 36 hpi

Protein name	Uniport accession No.	Log_2 ratios (infection/control)	peptides	Sequence coverage (%)	P-values	functions
**Upregulated proteins in PK-15-infected cells (a total of 426 proteins)**						
DNA topoisomerase I	I3LTP0	1.608012	1	2	0.008676	Ribonucleotide binding
DNA_MISMATCH_REPAIR_2 domain-containing protein	I3L5Z8	1.389998	2	2	0.008423	Function unknown
FIP-RBD domain-containing protein	A0A4X1UDM9	1.374843	1	32	0.01386	Immune system process;
Vitronectin	P48819	1.369345	2	4	0.009992	Function unknown
Uncharacterized protein	A0A4X1V7R2	1.344087	1	0	0.001621	Positive regulation of cellular process; binding
Protein S100-A14 isoform X1	F1SFV3	1.341646	2	15	0.01064	Function unknown
Uncharacterized protein	A0A4X1U802	1.340422	1	0	0.007536	Function unknown
Nebulin isoform X24	A0A480QQC5	1.303187	1	0	0.00395	Defense response to other organism; peptidase activity, acting on L-amino acid peptides
2'-5'-oligoadenylate synthase-like protein isoform a	F1RIV0	1.275293	13	44	0.0376	Organic cyclic compound binding
Uncharacterized protein	A0A5G2QLT7	1.179408	2	4	0.006756	Function unknown
mRNA-capping enzyme	A0A4X1VEE3	1.179021	3	7	0.01494	Function unknown
Uncharacterized protein	A0A4X1WD16	1.165596	1	6	0.003765	Function unknown
UPAR/Ly6 domain-containing protein	A0A287B805	1.163197	3	45	0.004268	Function unknown
FAM184 domain-containing	I3LV88	1.130018	1	1	0.004733	Function unknown

protein						
Required for excision 1-B domain-containing protein isoform 2	A0A287BRG2	1.116044	2	11	0.03597	Function unknown
CCHC-type domain-containing protein	F1SMW0	1.116037	2	3	0.01683	Function unknown
Gc-globulin	A0A4X1T9I2	1.113611	2	8	0.004535	Receptor binding
Mitochondrial fission factor	A0A4X1U473	1.112803	2	53	0.02686	Negative regulation of cellular process;
Apolipoprotein D	A0A5K1TWC1	1.096848	3	14	0.000102	Function unknown
Exocyst complex component 2	A0A287AU70	1.080402	1	23	0.00272	Function unknown
Ribonuclease H	A0A5G2R922	1.065412	1	7	0.04733	Peptidase activity, acting on L-amino acid peptides
Uncharacterized protein	A0A5G2RNJ9	1.02267	1	2	0.02215	Negative regulation of blood coagulation; protein binding
Plasminogen	P06867	1.003582	10	15	0.02445	Function unknown
DALR anticodon-binding domain-containing protein 3 isoform 1	F1SKI7	0.962701	1	2	0.03331	Negative regulation of cellular process;
Uncharacterized protein	A0A287A6X8	0.954095	1	2	0.00691	Positive regulation of cellular process;
Apolipoprotein A-I	K7GM40	0.940785	5	20	0.00224	Function unknown
RNF111_N domain-containing protein	A0A4X1URL2	0.929774	1	6	0.003849	Function unknown
Collagen IV NC1 domain-containing protein	A0A4X1U499	0.927763	1	1	0.03392	Peptidase inhibitor activity
Apolipoprotein H	A0A140TAK8	0.927612	3	13	0.01121	Function unknown
Uncharacterized protein	A0A287AZI1	0.918229	2	9	0.001764	Cholesterol efflux; peptidase activity, acting on L-amino acid peptides
Apolipoprotein M	A0A287AVQ5	0.917947	4	25	0.01145	Function unknown
RING-type domain-containing	A0A5G2QXW7	0.915669	3	16	0.01611	Function unknown

protein						
Uncharacterized protein	A0A5G2QJ57	0.8835	2	5	0.02457	Function unknown
Transmembrane protein 147	A0A4X1TJ50	0.863388	2	13	0.04743	Positive regulation of cellular process;
Steroidogenic acute regulatory protein, mitochondrial	Q5Q0U1	0.838784	1	2	0.001353	Cellular biosynthetic process;
Alpha-1,4-N-acetylglucosaminyltransferase	A0A0B8RZT0	0.833832	3	12	0.03689	Biological adhesion;
Ig-like domain-containing protein	A0A5G2QW86	0.816222	2	44	0.003373	Nitric oxide metabolic process; peptidase activity, acting on L-amino acid peptides
Nitric-oxide synthase (NADPH) (Fragment)	B4YYD8	0.798366	1	1	0.03782	Transferase activity, transferring hexosyl groups
ANK_REP_REGION domain-containing protein	A0A4X1U612	0.794568	1	14	0.008972	Function unknown
Nuclear autoantigen Sp-100 isoform 4	R4L6V8	0.7902	2	28	0.001253	Positive regulation of cellular process;
C3-beta-c	I3LTB8	0.777353	4	2	0.01585	Function unknown
Uncharacterized protein	A0A4X1SYC3	0.777089	1	2	0.03824	Binding
Vitellogenin domain-containing protein	F1SCV9	0.767211	16	3	0.004199	Function unknown
Uncharacterized protein	A0A4X1V704	0.764547	1	1	0.03549	Function unknown
CCR4-NOT transcription complex subunit 11	F1STG2	0.760944	4	16	0.008723	Scavenger receptor activity
Uncharacterized protein	A0A5G2QG99	0.751707	1	0	0.02094	Function unknown
Uncharacterized protein	A0A4X1TX07	0.749423	2	14	0.003484	Function unknown
Alpha-2-macroglobulin isoform a	A0A480SMD8	0.746027	4	2	0.02013	Function unknown

RING-type domain-containing protein	A0A287B7H5	0.733725	1	2	0.002055	Hydrogen peroxide metabolic process;
Hemoglobin subunit epsilon	F1RII6	0.73326	1	13	0.005017	Function unknown
Uncharacterized protein	A0A4X1UW90	0.722423	27	18	0.01868	Function unknown
Galectin	A0A287A342	0.720528	11	45	0.0139	Function unknown
Homeobox protein cut-like	A0A4X1U3F5	0.71351	2	2	0.03109	Function unknown
VWFA domain-containing protein	A0A4X1SK62	0.699449	1	9	0.002507	Function unknown
J domain-containing protein	A0A4X1VG11	0.689434	5	46	0.007554	Function unknown
MYND-type domain-containing protein	A0A4X1VY99	0.687404	5	18	0.005013	Extracellular structure organization; binding
GFO_IDH_MocA domain-containing protein	A0A4X1UEI6	0.685007	1	2	0.01032	Signal transduction;
Uncharacterized protein	A0A4X1SW44	0.681491	3	2	0.04938	Multicellular organism development; nucleoside-triphosphatase regulator activity
Translocon-associated protein subunit beta	A0A480W8D2	0.681344	1	3	0.01545	Function unknown
Ferritin	A0A4X1VYT5	0.677644	7	37	0.00614	Oxidoreductase activity
Tumor necrosis factor receptor superfamily member 10C	A0A480PT21	0.67473	1	6	0.007144	Function unknown
Serine incorporator 3	A0A480PEC3	0.673034	2	3	0.000596	Nucleoside-triphosphatase activity
Aminopeptidase	K9J6I5	0.670245	3	38	0.005152	Ribonucleotide binding
Voltage-dependent anion-selective channel protein 2	A0A4X1UZQ9	0.667953	1	15	0.003067	Lipid binding
Uncharacterized protein	F1SM15	0.667645	1	1	0.004416	Function unknown
Armadillo repeat-containing protein 4 isoform X3	A0A480MFY5	0.665857	2	2	0.006089	Function unknown

Reverse transcriptase domain-containing protein	A0A5G2QY09	0.665604	1	3	0.001643	Integrin binding
Uncharacterized protein	A0A4X1VY50	0.665239	1	1	0.001571	Positive regulation of cellular process;
DNA-dependent activator of IFN-regulatory factor	B8XX91	0.661183	6	46	0.0129	Function unknown
Arf-GAP with Rho-GAP domain, ANK repeat and PH domain-containing protein 1 isoform c	A0A481BP67	0.657348	1	10	0.008637	Receptor binding
GTPase-activating Rap/Ran-GAP domain-like protein 3 isoform X1	I3LIY1	0.655506	1	1	0.007202	Odontogenesis;
Aquaporin-5	A0A4X1WCJ0	0.65041	1	2	0.02023	Protein binding
Protein FAM173A isoform 1	A0A481BZ18	0.64809	6	40	9.80E-05	Triglyceride lipase activity
Uncharacterized protein	A0A5G2RAX9	0.639818	1	1	0.03078	Function unknown
Phospholipid-transporting ATPase	M3TYF0	0.639109	1	1	0.009552	Nitrogen compound metabolic process;
Ribosomal RNA processing protein 36 homolog isoform 1	A0A480TNH7	0.635574	5	24	0.02842	Type I interferon signaling pathway; protein heterodimerization activity
Interferon alpha/beta receptor 1	A0A4X1TWF4	0.635331	1	3	0.001839	Positive regulation of cellular process;
Clusterin	A0A5S8KLN1	0.631485	12	34	0.01402	Protein binding
Peptidase M12B domain-containing protein	F1SHP1	0.626314	9	14	0.002429	Molecular carrier activity
Protein kinase domain-containing protein	A0A287AK13	0.617633	4	7	0.004804	Negative regulation of cellular process;

RING-type domain-containing protein	F1STG1	0.615879	2	5	0.03779	Peptidase inhibitor activity
3-hydroxy-3-methylglutaryl coenzyme A reductase	A0A4X1SQE9	0.615048	8	11	0.00588	Positive regulation of cellular process;
Histone acetyltransferase	A0A4X1UTM8	0.614914	10	26	0.01368	Function unknown
Aprotinin (Fragment)	B0LXF7	0.611489	1	26	0.004965	Microtubule-based movement; peptidase activity, acting on L-amino acid peptides
Kinesin-like protein	A0A4X1TA22	0.609534	6	12	0.01341	Macromolecule glycosylation;
Beta-1,4-glucuronyltransferase 1	V9LLL5	0.607815	4	15	0.004261	Transmembrane transporter activity
Olfactory receptor	A0A5G2QYK1	0.598738	1	2	0.04429	Function unknown
dTMP kinase	A0A480PP50	0.588833	2	60	0.006871	Function unknown
Complex I-KFYI	A1XQS6	0.587608	2	24	0.04307	Function unknown
CCDC66 domain-containing protein	I3L7C7	0.580054	1	1	0.04985	Aromatic compound catabolic process;
CCR4-NOT transcription complex subunit 9	A0A5G2QHJ4	0.579651	9	34	0.003771	Function unknown
Putative E3 ubiquitin-protein ligase HERC6 isoform 1	A0A480M1Q1	0.579436	1	33	0.01541	Response to stimulus; protein binding
Importin N-terminal domain-containing protein	A0A4X1ST59	0.578024	1	43	0.00387	Positive regulation of cellular process;
Glycoprotein (Fragment)	Q9XSA8	0.576034	1	75	0.00077	DNA metabolic process;
CBFD_NFYB_HMF domain-containing protein	A0A287AUX2	0.571157	3	33	0.02543	Positive regulation of protein phosphorylation;
Insulin	P01315	0.570898	2	26	0.002678	Hormone activity
MHC class I antigen	A1YH92	0.565385	4	22	0.03329	Heterocyclic compound binding
WD_REPEATS_REGION	A0A4X1VPC0	0.564199	2	4	0.000832	Function unknown

domain-containing protein						
SERPIN domain-containing protein	A0A4X1U844	0.562966	7	20	0.007007	Alcohol transmembrane transporter activity
Uncharacterized protein	A0A4X1UQJ8	0.557041	2	10	0.01275	Function unknown
SERPIN domain-containing protein	A0A4X1U6E7	0.555549	7	17	0.007761	Ribonucleotide binding
Uncharacterized protein	A0A4X1VV99	0.544986	2	4	0.00179	Metal ion transport;
Ferritin	A0A4X1VGP9	0.544938	8	56	0.01707	Function unknown
Uncharacterized protein	A0A5G2QG05	0.544345	3	11	0.00704	Ubiquitin-like protein ligase activity
PRELI domain-containing protein 3B	A0A287AXX7	0.54228	4	34	0.01647	Protein modification process; organic cyclic compound binding
Glyco_transf_24 domain-containing protein	A0A5G2Q988	0.539164	1	49	0.009863	Ion binding
FERM domain-containing protein	A0A4X1SDT9	0.537789	2	3	0.0459	Function unknown
Uncharacterized protein	A0A4X1U394	0.534186	2	20	0.002162	Binding
Apolipoprotein A2 (Fragment)	Q7YRR7	0.532336	1	53	0.03981	DNA binding
Aminopeptidase	K9J4P1	0.531619	7	7	0.02988	Binding
Pleckstrin homology-like domain family A member 1	F1SGD8	0.531029	1	2	0.02619	Function unknown
Uncharacterized protein	F6PUJ1	0.529644	1	9	0.0156	RNA binding
Tripartite motif-containing protein 26	B6ICV1	0.529153	5	11	0.002027	Positive regulation of cellular process;
Sterol regulatory element-binding protein 1	O97676	0.52799	2	2	0.02317	Response to stimulus;
Prothrombin	A0A4X1VLZ9	0.526259	6	9	0.008947	Function unknown

Interferon-related developmental regulator 2	A0A480RN29	0.525085	3	12	0.003586	Positive regulation of cellular process;
Beta-2-microglobulin	A0A4X1VG41	0.523692	3	32	0.01886	Small molecule metabolic process;
Selenide, water dikinase 2	A1YIZ1	0.523492	5	18	0.03011	Function unknown
Integrin alpha-3 preproprotein	A0A480ZI48	0.522856	27	33	0.00386	Cellular component organization or biogenesis;
HAUS6_N domain-containing protein	A0A5G2QNR3	0.521341	8	12	0.000392	Function unknown
Uncharacterized protein	F1SRY9	0.51995	6	20	0.04941	Function unknown
Ubiquitin carboxyl-terminal hydrolase	A0A4X1UQ80	0.517469	12	17	0.002366	Inositol phosphate phosphatase activity
RING-type domain-containing protein	F1S3S2	0.514378	21	33	0.005338	Protein binding
Zinc finger protein 75D isoform 1	A0A480WRF9	0.512281	2	6	0.04484	Function unknown
Uncharacterized protein	A0A4X1T205	0.509032	8	35	0.000136	Function unknown
Uncharacterized protein	F1SL31	0.506237	1	34	0.001654	Function unknown
Carbohydrate sulfotransferase (Fragment)	A0A480V2S8	0.503091	3	9	0.01965	Regulation of microtubule cytoskeleton organization;
Spindle and kinetochore-associated protein 1	A0A4X1UHG2	0.501485	2	10	0.000423	Function unknown
Uncharacterized protein	A0A4X1VCI3	0.500037	1	7	0.02448	Positive regulation of cellular process;
C-type lectin domain-containing protein	A0A4X1V3B9	0.500018	1	3	0.006962	Ion binding
TLDc domain-containing protein	A0A4X1UQ31	0.499408	7	43	0.03456	Protein dimerization activity
Uncharacterized protein	A0A5G2QC13	0.497516	2	8	0.001074	Function unknown

Uncharacterized protein	F1SBB3	0.490343	33	12	0.002933	Function unknown
Bifunctional apoptosis regulator isoform 1	A0A480KEY4	0.489213	2	8	0.001272	Organic cyclic compound binding
Uncharacterized protein	A0A4X1UAA6	0.488351	6	5	0.04062	Function unknown
t-SNARE coiled-coil homology domain-containing protein	F1RI88	0.487794	8	30	0.002533	UDP-glycosyltransferase activity
Tyrosine-protein phosphatase non-receptor type 3 isoform X3	A0A480PP88	0.485733	3	4	0.04502	Function unknown
TLDc domain-containing protein	I3LMG2	0.484843	1	45	0.04937	Morphogenesis of an epithelium;
Protein fem-1 homolog B	I3L6T4	0.484429	5	10	0.02108	Negative regulation of cellular process;
Albumin	A0A287BAY9	0.483031	9	12	0.002365	Thrombospondin receptor activity
Tetratricopeptide repeat protein 14 isoform a	A0A287AVL3	0.480315	1	1	0.02335	Chromosome organization;
Follicle-stimulating hormone primary response protein 1 (Fragment)	Q5K2Q1	0.47309	1	14	0.03252	Protein ubiquitination;
Ankyrin repeat and SOCS box protein 3 isoform X1	F1SQJ4	0.472508	1	2	0.03683	Fatty acid elongation, saturated fatty acid;
Elongation of very long chain fatty acids protein (Fragment)	B3VHV3	0.472016	2	6	2.50E-05	Transferase activity, transferring hexosyl groups
DNAJ homolog subfamily C member 7	A0A480W4L0	0.471488	1	53	0.04066	Function unknown
MateRNAl embryonic leucine zipper kinase	F1ST84	0.468102	4	8	0.04776	Macromolecule metabolic process; protein binding

Proteasome subunit beta	Q09YA9	0.46701	7	33	0.02331	Function unknown
Prostaglandin F2 receptor negative regulator	A0A480JG52	0.46445	2	40	0.0344	Function unknown
Uncharacterized protein	A0A4X1THV0	0.463487	3	39	0.003107	Catalytic activity, acting on DNA
Eyes absent homolog	A0A4X1TSB9	0.460583	1	12	0.04591	Positive regulation of cellular process;
WD_REPEATS_REGION domain-containing protein	A0A4X1ST95	0.459332	5	14	0.000418	Function unknown
AMMECR1 domain-containing protein	A0A4X1VSW4	0.458672	3	17	0.001042	Positive regulation of cellular process; translation initiation factor activity
ADP-ribosylation factor-like protein 6-interacting protein 1	K9IVS1	0.457816	3	23	0.01166	Function unknown
Laminin subunit gamma-2 isoform a	F1S662	0.455673	36	38	0.01372	Function unknown
Uncharacterized protein	F1S0N9	0.455627	6	2	0.03153	Negative regulation of cellular process; sugar-phosphatase activity
Tsukushin	A0A480V1E4	0.45443	1	8	0.000382	Function unknown
Protein kinase domain-containing protein	A0A4X1V396	0.452426	2	3	0.008773	Regulation of biological process;
VWFA domain-containing protein	A0A4X1TDX0	0.450492	3	3	0.001859	Function unknown
Tether containing UBX domain for GLUT4 isoform 2	A0A480NU10	0.449803	3	34	0.01429	Regulation of blood coagulation;
Antithrombin-III	A0A480P2S5	0.449632	6	12	0.000731	Positive regulation of cellular process;
PH domain-containing protein	A0A4X1SI48	0.442961	1	7	0.005458	Function unknown
Uncharacterized protein	A0A4X1VFS0	0.440331	4	11	0.007646	Microtubule cytoskeleton organization; binding
Kinetochore protein Nuf2	A0A480SGE8	0.439108	8	22	0.001652	Function unknown
Uncharacterized protein	K7GMF6	0.438335	9	15	0.00131	Response to stimulus; mRNA binding

SLA-8	Q6S7D1	0.434535	9	41	0.002318	Positive regulation of cellular process;
Uncharacterized protein	F1RM98	0.433283	6	19	0.01873	Function unknown
Uncharacterized protein	A0A287A841	0.432281	2	34	0.000963	Transmembrane transporter activity
ASD2 domain-containing protein	A0A4X1TVX4	0.431572	1	13	0.04487	Myeloid dendritic cell activation;
StAR-related lipid transfer protein 7, mitochondrial	F1SU33	0.428269	15	44	0.001089	Function unknown
[Histone H3]-trimethyl-L-lysine(9) demethylase	A0A4X1W805	0.425629	7	8	0.000702	Positive regulation of cellular process;
Claudin	C3VML0	0.425049	3	21	0.02395	Function unknown
G_PROTEIN_RECEP_F3_4 domain-containing protein	A0A4X1V7D7	0.424796	4	14	0.02649	Regulation of biological process;
Glutaredoxin domain-containing protein	A0A5G2QMG6	0.424339	5	21	0.03122	Function unknown
Uncharacterized protein	A0A4X1VM59	0.418637	6	59	0.005662	Function unknown
Little elongation complex subunit 1	A0A480ZUS4	0.418542	5	2	0.03733	Function unknown
Component of oligomeric Golgi complex 2	A0A480K7Y8	0.418149	10	17	0.01765	Binding
Uncharacterized protein	A0A4X1U745	0.417722	6	8	0.003205	Function unknown
Hyaluronoglucosaminidase	F1SJB9	0.417343	3	2	0.02041	Binding
Integrator complex subunit 14	A0A5K1U644	0.41712	5	16	0.005408	Function unknown
Uncharacterized protein	K7GR94	0.416758	7	22	0.000846	UDP-glucuronate decarboxylase activity
Serine/threonine-protein kinase PLK	A0A4X1UYN5	0.415803	4	30	0.009773	Transcription, DNA-templated;
SHS2_Rpb7-N domain-	F1SC14	0.413302	5	18	0.000754	Function unknown

containing protein						
Solute carrier family 30 member 2	B5A8K5	0.41314	1	3	0.01211	Signal transduction;
Delta-like protein	A0A480SJP0	0.412715	1	1	0.04024	Function unknown
ER membrane protein complex subunit 10	A0A480I708	0.412182	3	21	0.000662	Function unknown
Uncharacterized protein	A0A4X1U6L2	0.410847	2	1	0.003555	Positive regulation of cellular process; binding
RFX-type winged-helix domain-containing protein	A0A4X1W7L4	0.408226	1	1	0.002736	Protein catabolic process;
Proteasome subunit beta	Q2PYM7	0.406666	6	37	0.03296	Protein transport;
Mitochondrial import inner membrane translocase subunit Tim10 B	A0A5G2R1A5	0.405398	3	48	0.008737	Function unknown
XIAP-associated factor 1	K9IVW8	0.404605	6	29	0.02781	Positive regulation of cellular process;
Metalloproteinase inhibitor 3	A0A286ZZX3	0.404373	3	12	0.00138	Function unknown
Alkylated DNA repair protein alkB homolog 8	I3LA93	0.40321	8	18	0.000905	Function unknown
Formyltetrahydrofolate synthetase	A0A480VMB4	0.402403	2	47	0.0319	Function unknown
C2H2-type domain-containing protein	A0A4X1VQG1	0.402077	4	39	0.02873	Positive regulation of cellular process;
UBC core domain-containing protein	A0A4X1U068	0.401283	9	66	0.00532	Cellular metabolic process; thrombospondin receptor activity
Palmitoyl acyl-CoA oxidase 1 (Fragment)	A7J0A9	0.401204	1	53	0.002402	Protein heterodimerization activity
Nuclear envelope pore	A0A480M484	0.40109	11	11	0.004646	Function unknown

membrane protein POM 121 isoform X1						
Uncharacterized protein	A0A286ZWB1	0.398841	7	35	0.000665	Function unknown
EEF1A lysine methyltransferase 1	A0A481DEK1	0.396999	4	21	0.008492	Function unknown
RanBP-type and C3HC4-type zinc finger-containing protein 1	F1S7J3	0.395297	2	6	0.0287	Function unknown
High mobility group AT-hook 1 transcript variant 2	B6CVL5	0.394024	1	51	0.003348	Function unknown
Uncharacterized protein	A0A4X1TIC9	0.393926	1	21	0.01303	Binding
RNA-binding protein 12	I3LLW0	0.39217	2	16	0.008001	Positive regulation of cellular component biogenesis; lysophospholipid acyltransferase activity
Hepatocyte growth factor receptor	Q2QLE0	0.391816	33	33	0.000129	Translational termination;
Uncharacterized protein	F1RJ87	0.391233	5	16	0.03001	Function unknown
Aa_trans domain-containing protein	A0A4X1TLM5	0.390207	5	10	0.0194	Function unknown
Protein tweety homolog	A0A4X1TMB9	0.389735	3	5	0.002111	Function unknown
TGF-beta receptor type-2	K7GL93	0.388737	6	11	0.004107	Function unknown
Uncharacterized protein	A0A4X1U767	0.388497	15	40	0.000157	Function unknown
Uncharacterized protein	A0A5G2RBZ9	0.38803	6	13	0.00635	Transcription factor activity, sequence-specific DNA binding
Sulfiredoxin	A0A4X1SMI5	0.38784	5	36	0.01874	Transferase activity, transferring hexosyl groups
Inter-alpha-trypsin inhibitor heavy chain H3 preproprotein (Fragment)	A0A480KGP4	0.387723	4	7	0.000499	Function unknown
Uncharacterized protein	A0A4X1TI35	0.387714	1	53	0.01874	Protein transport; RNA 7-methylguanosine cap binding

AP-1 complex subunit gamma	F1SS42	0.387697	9	15	0.01399	Regulation of biological process;
Ig-like domain-containing protein	F1RPU6	0.387144	6	22	0.005653	Function unknown
Envelope protein	Q6W4V3	0.38701	4	38	0.02135	Macromolecule metabolic process; GTP binding
TYR_PHOSPHATASE_2 domain-containing protein	A0A4X1W6Q1	0.385963	3	11	0.02979	Macromolecular complex binding
Sema domain-containing protein	A0A4X1ULG5	0.385117	2	47	0.0287	Function unknown
Laminin subunit alpha-1 (Fragment)	A0A480XDX5	0.383068	1	17	0.02095	Function unknown
UPF0669 protein C6orf120 isoform 2	I3LL77	0.382963	2	17	0.008982	Function unknown
Vesicular integral-membrane protein VIP36 (Fragment)	A0A480VX24	0.38261	10	49	0.004954	Negative regulation of cellular process; mannose binding
Uncharacterized protein	A0A4X1U5R9	0.382575	2	67	0.02371	Translation release factor activity
p21-activated protein kinase-interacting protein 1	A0A480YUK6	0.382338	14	51	0.02774	Function unknown
Pentatricopeptide repeat-containing protein 1, mitochondrial	A0A480UCS6	0.382082	6	9	0.000551	Positive regulation of cellular process;
Cyclin B1 transcript variant 1	D2IE28	0.380655	6	19	0.01711	Peptide metabolic process; phosphatidylinositol phosphate phosphatase activity
Gamma-secretase subunit APH-1A isoform 1	A0A287B0C6	0.379177	2	5	0.003874	Catalytic activity, acting on a protein
Mitochondrial genome maintenance exonuclease 1	A0A4X1UZ36	0.378964	11	45	0.002142	Function unknown
Uncharacterized protein	A0A4X1VZD8	0.378868	20	64	0.003449	Peptidase activity, acting on L-amino acid peptides

N-myc downstream-regulated gene 3 protein	F1S479	0.378783	8	28	0.002464	Artery development; galactosyltransferase activity
Dynactin subunit 5	A0A286ZK88	0.378607	2	10	0.03874	Function unknown
Uncharacterized protein	F1SRW7	0.376004	1	0	0.03556	RNA processing;
Ribosome biogenesis protein NSA2 homolog	A0A5G2QRK0	0.375581	11	45	0.001679	Synaptic signaling;
Uncharacterized protein	A0A4X1SMZ2	0.37489	6	33	0.01552	Cellular process;
Importin subunit alpha	I3LS70	0.372586	21	58	0.002582	Function unknown
Large neutral amino acids transporter small subunit 1	K9IVK6	0.371282	8	14	0.01764	Terpenoid transport;
Retinol-binding protein	F1SC80	0.371107	9	72	0.03345	Autopha; protein binding
Transmembrane protein 41B isoform X1	A0A286ZID7	0.37043	3	9	0.04224	Antigen processing and presentation of peptide antigen; 3-oxo-lignoceronyl-coa synthase activity
Ig-like domain-containing protein	A0A287A1Y1	0.370374	6	23	0.02009	Function unknown
Uncharacterized protein	A0A5G2R8T2	0.36903	2	7	0.003563	Cellular protein modification process; binding
TYR_PHOSPHATASE_2 domain-containing protein	A0A4X1W0Q7	0.365945	3	38	0.0376	Positive regulation of cellular process;
Solute carrier family 25 member 33 (Fragment)	A0A480JH88	0.365364	5	17	0.008218	Function unknown
Essential for reactive oxygen species protein	A0A5G2QFI7	0.364868	4	15	0.00833	Function unknown
Uncharacterized protein	A0A5G2QK26	0.364558	1	1	0.01697	Function unknown
Mitoribosomal protein bs6m, mrps6	A0A5K1V0G5	0.36346	6	46	0.01749	Organophosphate metabolic process;
1-acylglycerol-3-phosphate O-	D0G0C1	0.359012	5	13	0.01967	Binding

acyltransferase 4						
Uncharacterized protein	F1SG83	0.35864	9	10	0.002888	Function unknown
Uncharacterized protein	I3LKY5	0.358548	14	55	0.001858	Positive regulation of cellular process;
Arginase	A0A5S8LD81	0.357926	3	13	0.03813	Transferase activity, transferring glycosyl groups
Protein BANP	A0A481CG23	0.35778	2	6	0.03975	Positive regulation of cellular process;
Integrin-associated protein	A0A4X1SPG5	0.356919	2	14	0.007569	Function unknown
ILEI domain-containing protein	A0A5G2R617	0.356399	4	21	0.007804	Ribonucleotide binding
Integral membrane protein 2	A0A480V4R2	0.355964	3	70	0.005554	Serine-type peptidase activity
Cytochrome P450 3A4 isoform 1	A0A480VJX3	0.355695	1	42	0.000675	Ribonucleotide binding
Twinkle protein, mitochondrial isoform A	I3LNP8	0.351642	4	8	0.001883	Function unknown
G1 to S phase transition 2	F1RUJ9	0.351333	2	21	0.00399	Positive regulation of cellular process;
Uncharacterized protein	A0A4X1UQ65	0.350275	4	28	0.02476	Function unknown
Small integral membrane protein 26	I3LCG7	0.349361	3	31	0.001362	Peptide metabolic process;
Signal peptidase complex catalytic subunit SEC11	A0A481CTH0	0.346439	5	18	0.002162	Protein ubiquitination; binding
RING-type E3 ubiquitin transferase	A0A287ALB7	0.345939	2	6	0.02907	Function unknown
Uncharacterized protein	A0A5G2QEV8	0.345349	7	10	0.03842	Cation transmembrane transport;
Uncharacterized protein	A0A286ZYM2	0.344587	7	30	0.01904	Function unknown
AB hydrolase-1 domain-containing protein	A0A4X1VWB4	0.344448	2	9	0.02407	Positive regulation of cellular process; transmembrane transporter activity
Noelin-2 isoform 2	A0A480XA75	0.343091	1	2	0.02186	Iron-sulfur cluster binding
Tax1-binding protein 1 isoform	A0A480K2R0	0.342831	3	3	0.00308	Optic nerve development; inward rectifier potassium channel inhibitor activity

1						
Transmembrane protein 126A isoform 1	I3LJK4	0.340829	6	25	0.000183	Potassium ion import across plasma membrane;
Uncharacterized protein	A0A4X1SPI0	0.34037	5	10	0.02582	C4-dicarboxylate transport;
Pannexin_like domain-containing protein	A0A286ZWM5	0.340362	7	10	0.001193	Protein dimerization activity
40S ribosomal protein S23 (Fragment)	Q29298	0.340232	1	23	0.01787	Function unknown
Forty-two-three domain-containing protein 1	A0A4X1TP41	0.340093	3	14	0.0202	Binding
Uncharacterized protein	A0A4X1VTD1	0.340005	2	53	0.02167	DNA-directed RNA polymerase activity
Nucleolar transcription factor 1 isoform X1	F1RQY4	0.33834	1	40	0.01288	Cellular biosynthetic process; DNA binding
Hexosyltransferase	F1S937	0.338327	5	17	0.006929	Function unknown
Solute carrier organic anion transporter family member	A0A287A0K8	0.337834	2	2	0.04983	Cellular localization; channel activity
Exocyst complex component	A0A480UT78	0.337021	4	8	0.01824	Transferase activity, transferring hexosyl groups
CP-type G domain-containing protein	A0A4X1W3X9	0.336245	26	54	0.000464	Chromatin binding
Peptidyl-prolyl cis-trans isomerase	A0A287AP21	0.336014	7	38	0.0106	Function unknown
Tetraspanin	A0A4X1VZD4	0.335804	1	3	0.00146	RNA binding
SANT domain-containing protein	A0A4X1SJM1	0.335667	3	5	0.000668	Transcription factor activity, sequence-specific DNA binding
Methionine--tRNA ligase	A0A4X1VG66	0.335086	12	30	0.001792	TRNA aminoacylation for protein translation;
Histidine--tRNA ligase	F1RGD8	0.334276	11	41	0.002018	Small molecule metabolic process; active ion transmembrane transporter activity

Glyco_transf_64 domain-containing protein	A0A4X1TSD3	0.333328	1	2	0.01947	Function unknown
Uncharacterized protein	F1SS06	0.333051	1	1	0.006313	Response to endoplasmic reticulum stress;
J domain-containing protein	A0A4X1T6M4	0.331497	7	20	0.01098	Protein ubiquitination;
HECT-type E3 ubiquitin transferase	A0A4X1T1L2	0.330894	6	20	0.04313	Function unknown
Thymidine kinase	A0A286ZID2	0.330187	10	45	0.04214	RNA binding
Eukaryotic elongation factor 2 kinase	A0A480WZ84	0.328616	11	18	0.018	Mitotic cell cycle checkpoint;
DNA topoisomerase 2-alpha	O46374	0.328201	64	47	0.000515	Function unknown
Uncharacterized protein	F1S4Q6	0.328008	3	45	0.01816	Aromatic compound biosynthetic process;
Radical S-adenosyl methionine domain-containing protein	A0A5G2R1S6	0.326581	1	2	0.01058	Regulation of biological process; ubiquitin-like protein ligase activity
PCNA-associated factor	A0A5G2QZV8	0.325984	1	6	0.03608	DNA metabolic process;
CBFD_NFYB_HMF domain-containing protein	A0A4X1V1W7	0.32572	3	24	0.01174	Lipase activity
Uncharacterized protein	A0A5G2R2X8	0.324931	1	5	0.02414	Carbohydrate metabolic process;
4F2 cell-surface antigen heavy chain isoform b	A0A481D7K6	0.324575	20	46	0.005092	Function unknown
RNA helicase	A0A480I8F5	0.323738	9	16	0.01436	Function unknown
GCFC domain-containing protein	I3L6B6	0.323689	1	1	0.02888	Ribosomal large subunit assembly; metal ion binding
Ribosome assembly factor mrt4	A0A287A384	0.32281	14	54	0.007836	Signal transduction;
Uncharacterized protein	A0A4X1UNF8	0.322658	6	11	0.007857	Macromolecular complex binding

Kinetochore protein Spc24	A0A287B6S8	0.322607	8	45	0.004668	Function unknown
Uncharacterized protein	A0A4X1UJN1	0.322408	4	14	0.001864	Positive regulation of cellular process; transition metal ion binding
Nuclear cap-binding protein subunit 1 isoform 1	A0A480LT67	0.32209	4	45	0.0189	Anatomical structure development; integrin binding
EABR domain-containing protein	A0A287AZV2	0.321768	13	32	0.004702	Function unknown
Rhomboid domain-containing protein	A0A287B167	0.321206	6	16	0.03443	Anion binding
RING-type E3 ubiquitin transferase	A0A5G2R8C3	0.320853	2	3	0.009024	RNA processing; peptidyl-prolyl cis-trans isomerase activity
Ribosomal RNA-processing protein 43	A0A4X1T3H1	0.320023	8	33	0.001722	Macromolecule metabolic process;
Ubiquitinyl hydrolase 1	A0A4X1SI54	0.318744	3	16	0.02854	Positive regulation of cellular process;
Cytochrome c oxidase copper chaperone	D2XUP7	0.318482	3	59	0.01172	Function unknown
rRNA adenine N(6)-methyltransferase	A0A480VKC7	0.318415	6	32	0.0121	Function unknown
Exonuclease domain-containing protein	A0A4X1VXR2	0.317153	8	30	0.000623	Function unknown
DNAJ homolog subfamily B member 14 isoform 1	A0A287AP60	0.315087	6	15	0.01357	Protein binding
Non-receptor tyrosine-protein kinase TNK1 isoform X1	A0A480QM13	0.314991	9	19	0.01936	Cellular response to alcohol; oxidoreductase activity
Adrenodoxin, mitochondrial	P00258	0.31485	7	37	0.003011	Function unknown
FAD-binding PCMH-type domain-containing protein	A0A4X1SXF0	0.314644	9	23	0.006716	Organic hydroxy compound metabolic process;

GP-PDE domain-containing protein	A0A4X1VIV5	0.314504	7	23	0.02174	Function unknown
Peptidylprolyl isomerase	A0A287B5T6	0.314469	4	43	0.004683	Binding
Uncharacterized protein	A0A4X1UD59	0.314218	15	34	0.02837	Heterocyclic compound binding
AAA domain-containing protein	A0A287BJX1	0.314129	9	6	7.00E-06	Positive regulation of cellular process;
Integrin beta	A0A480JX12	0.313969	25	41	0.002313	Protein heterodimerization activity
Uncharacterized protein	A0A287BKK7	0.313873	5	17	0.000364	Regulation of transcription from RNA polymerase II promoter;
Max-like protein X isoform gamma	F1S1D8	0.313359	3	17	0.005952	RNA processing; binding
RNA-binding protein NOB1	F1S396	0.313008	15	44	0.003561	Endonuclease activity
Semaphorin-3C	A0A4X1SIW8	0.312767	16	27	0.006512	Function unknown
Hexosyltransferase	A0A287AWP7	0.312636	3	7	0.003797	Function unknown
Uncharacterized protein	A0A4X1V1X7	0.312217	3	5	0.01796	Function unknown
Uncharacterized protein	A0A4X1SVJ5	0.310974	25	34	0.00357	Transition metal ion binding
Major centromere autoantigen B	A0A480VMC6	0.310367	2	2	0.00599	Positive regulation of cellular process; protein binding
Sodium-coupled neutral amino acid transporter 2 isoform 1	K9J4Q4	0.310346	3	12	0.0159	RNA processing;
ADAR adenosine deaminase RNA specific	K9J4M6	0.309792	43	46	0.0175	Aromatic compound biosynthetic process; metal ion binding
DNA-directed RNA polymerase I subunit D	A0A4X1TN10	0.309734	8	32	0.002069	Function unknown
ETS domain-containing protein	A0A4X1SJQ1	0.309576	2	4	0.009689	Potassium ion transmembrane transporter activity
Poly [ADP-ribose] polymerase	A0A4X1TD13	0.309349	45	31	0.04399	Receptor binding

Uncharacterized protein	A0A287BB92	0.309046	5	10	0.006345	Function unknown
Uncharacterized protein	A0A287ABK7	0.307761	12	12	0.000815	Function unknown
Nucleolar protein 10	A0A286ZQ67	0.307394	21	39	0.01232	Electron carrier activity
Proliferation marker protein Ki-67 isoform X1-like (Fragment)	A0A480VRH1	0.306846	2	44	0.04188	Function unknown
DNA-directed RNA polymerase	M3V845	0.306712	15	16	0.003357	Methylated histone binding
Uncharacterized protein	A0A5G2RD08	0.306695	1	54	0.02148	Function unknown
Folylpolyglutamate synthase	A0A4X1U4V3	0.306009	3	6	0.006191	Function unknown
Nicastrin	A0A4X1VVD1	0.304929	12	20	0.000152	Ubiquitin-dependent protein catabolic process; protein methyltransferase activity
UBC core domain-containing protein	A0A5G2R8X5	0.304311	4	24	0.02566	Transmembrane signaling receptor activity
Chitobiosyldiphosphodolichol beta-mannosyltransferase isoform 1	F1RKX2	0.30407	6	21	0.01854	Cellular metabolic process; organic cyclic compound binding
Succinate dehydrogenase [ubiquinone] cytochrome b small subunit, mitochondrial	A5GZW8	0.302987	1	4	0.03812	Macromolecule metabolic process; protein binding
ATP-dependent Clp protease ATP-binding subunit clpX-like, mitochondrial	A0A287ADU8	0.301699	30	54	6.50E-05	Sphin;
Serine palmitoyltransferase 3	F1SBJ7	0.301198	16	34	0.001862	Function unknown
Heparin cofactor 2	A0A480RKY5	0.300482	1	3	0.01494	Function unknown
UDP-glucuronate decarboxylase 1	A0A480TRQ6	0.300334	9	32	0.02099	Function unknown
Uncharacterized protein	A0A4X1W3E2	0.300106	1	2	0.005724	Function unknown

Apoptosis-inducing factor 2 isoform X4	A0A480J3T0	0.2996	7	31	0.01881	Function unknown
Proline dehydrogenase	M3V806	0.298944	8	18	0.02795	Transmembrane transporter activity
Centromere protein N	A0A480VG43	0.298703	2	9	0.03739	Function unknown
Uncharacterized protein	A0A4X1UNL2	0.297814	1	2	0.01112	Function unknown
PHD-type domain-containing protein	A0A4X1U359	0.297803	6	11	0.04966	Function unknown
Uncharacterized protein	A0A287BDP1	0.297694	1	25	0.008217	Endonucleolytic cleavage to generate mature 5'-end of SSU-rRNA from (SSU-rRNA, 5.8S rRNA, LSU-rRNA;
Activator of basal transcription 1	A0A287B3X6	0.296659	8	26	0.003525	High-density lipoprotein particle receptor binding
Uncharacterized protein	A0A287BNX8	0.296098	9	13	0.001687	Localization; binding
Solute carrier family 52, riboflavin transporter, member 2	A0A4X1UQJ3	0.295974	1	2	0.01098	Function unknown
Uncharacterized protein	A0A5G2Q7R0	0.295456	20	25	0.00101	Function unknown
DDB1- and CUL4-associated factor 13	A0A4X1TLA1	0.294688	14	38	0.003813	Ligase activity
Syndecan	A0A287B0S3	0.294624	3	9	0.0455	Macromolecule metabolic process;
Calumenin	A0A480YTG3	0.294576	1	73	0.04824	Heterocyclic compound binding
Cytochrome c oxidase assembly factor 5	A0A4X1W3F9	0.294573	7	16	0.004957	Peptidase activity, acting on L-amino acid peptides
Uncharacterized protein	F1RNK3	0.294531	4	16	0.01318	RNA binding
Eukaryotic translation initiation factor 1A domain-containing protein	F1RU32	0.293978	6	46	0.01701	Function unknown

Uncharacterized protein	A0A4X1SJF6	0.292888	14	33	0.000894	Positive regulation of cellular process; catalytic activity
Ras-related protein Rap-2a	Q06AU2	0.292687	6	63	0.007432	Function unknown
Centromere protein X	A0A5G2QZG8	0.292343	3	21	0.01992	Function unknown
Phosphoinositide phospholipase C	A0A480J5N4	0.291701	11	21	0.01357	3'-5'-exoribonuclease activity
Uncharacterized protein	A0A4X1T4W4	0.291254	26	13	0.04611	Function unknown
CD44 antigen	A0A4X1SP57	0.290887	8	22	0.01458	Cation transmembrane transport; transcription regulator activity
Uncharacterized protein	A0A4X1UJI9	0.290377	19	21	0.000377	Macromolecule metabolic process;
Proteasome subunit beta	A0A4X1V3H8	0.289135	6	28	0.008264	Negative regulation of cellular process;
Histone H2A	F2Z5L6	0.289076	2	70	0.01254	Enzyme regulator activity
Uncharacterized protein	A0A4X1U855	0.28767	7	17	0.01554	Function unknown
Multifunctional methyltransferase subunit TRM112-like protein	F1RQP1	0.287004	6	54	0.04026	RRNA binding
Hermansky-Pudlak syndrome 5 protein homolog	A0A4X1T7H6	0.286583	7	8	0.009038	Positive regulation of cellular process;
Baculoviral IAP repeat-containing protein 5	Q9GLN5	0.286404	8	62	0.006409	Small molecule metabolic process;
Alpha-fetoprotein	Q8MJ76	0.286236	3	4	0.01263	Function unknown
Kinesin-like protein	F1SIT8	0.285424	38	52	0.000282	Primary metabolic process;
Lipoprotein lipase	I3LUA0	0.284655	6	20	0.002814	Macromolecular complex binding
RNA polymerase III subunit C3	F1SDD7	0.284615	16	37	0.04637	Function unknown
Uncharacterized protein	F1SE89	0.284292	2	2	0.007334	Positive regulation of cellular process; small molecule binding
Integrin-associated protein	A0A287BQR8	0.284201	2	20	0.0207	Function unknown
Uncharacterized protein	A0A287ATS5	0.283852	2	6	0.03862	RNA processing;
S4 RNA-binding domain-	F1SJ73	0.283082	10	66	0.007847	Function unknown

containing protein						
LisH domain-containing protein	A0A287B9T7	0.283025	6	6	0.01429	Protein binding
Complex I assembly factor TIMMDC1, mitochondrial	A0A287BCV5	0.282626	2	9	0.03731	Phosphoprotein phosphatase activity
Uncharacterized protein	A0A4X1TMY0	0.282203	3	4	0.006574	Regulation of translation;
Pumilio homolog 3	A0A481CG74	0.281818	27	44	0.01491	Function unknown
Uncharacterized protein	F1RFN6	0.280773	21	23	0.002804	Pyridoxal phosphate binding
Uncharacterized protein	A0A4X1UQE7	0.279765	8	26	0.003737	Small molecule binding
Uncharacterized protein	A0A4X1UUL9	0.279094	1	30	0.03004	RNA processing; disulfide oxidoreductase activity
Polynucleotide phosphorylase 1	A0A287AMV5	0.278915	44	67	0.009326	Protein localization to organelle;
WD_REPEATS_REGION domain-containing protein	A0A4X1SFI9	0.278651	14	59	0.000112	Function unknown
MICOS complex subunit MIC10	A0A4X1W4D1	0.278615	1	10	0.01305	Function unknown
SURF6 domain-containing protein	A0A4X1SZR9	0.277941	12	39	0.005286	Small molecule metabolic process;
Inositol-1-monophosphatase	A0A287AVN0	0.27761	11	40	0.00396	Function unknown
Ubiquitin-like domain-containing protein	I3LPR9	0.277451	3	16	0.01288	Function unknown
Cationic amino acid transporter 2	A0A4X1TZW6	0.275748	2	5	0.008805	Carbohydrate derivative binding
MICOS complex subunit MIC60	A0A287AMM6	0.275167	2	64	0.04329	Active ion transmembrane transporter activity
BRCA2 and CDKN1A-	A0A4X1V9L9	0.275114	6	33	0.009938	Function unknown

interacting protein						
Nucleolar protein 16	I3LMC8	0.275082	11	59	0.002028	Cytoskeleton-dependent cytokinesis; mRNA binding
Uncharacterized protein	A0A287AGK4	0.274613	2	6	0.001911	Lipid binding
Uncharacterized protein	I3LF70	0.274169	27	40	0.007002	Intracellular transport;
Uncharacterized protein	A0A4X1U7W6	0.273407	11	42	0.001854	Function unknown
Ubiquitin-like protein 5	A0A4X1SNZ7	0.272834	4	41	0.001813	Positive regulation of cellular process;
Epithelial cell adhesion molecule	Q75QW1	0.272824	10	30	0.000463	Function unknown
NADPH:adrenodoxin oxidoreductase, mitochondrial	A0A4X1SGU8	0.27221	22	58	0.002425	RNA processing;
KRR1 small subunit processome component	A0A287BCI5	0.271598	18	41	0.006162	Function unknown
GBD/FH3 domain-containing protein	A0A4X1TYL3	0.271397	4	26	0.02175	Function unknown
Uncharacterized protein	A0A4X1T474	0.27065	7	20	0.04523	Function unknown
SH3 domain-containing protein	F1RW07	0.270505	11	7	0.003725	Function unknown
Uncharacterized protein	I3L5I1	0.269754	22	40	0.002682	Function unknown
Uncharacterized protein	F1STI9	0.269729	13	34	0.004674	Negative regulation of cellular process;
Transmembrane protein 109	F1RIA7	0.269641	4	13	0.000878	RNA processing;
Ribosome biogenesis protein WDR12	A0A4X1V7X7	0.269523	16	49	0.03658	Function unknown
Reticulon	A0A287B5Q4	0.269461	3	58	0.01966	Function unknown
Anion exchange protein	A0A480L1L0	0.26943	10	14	0.01888	Metal ion transmembrane transporter activity
Formyltetrahydrofolate synthetase	A0A4X1VLC6	0.268597	19	55	0.003057	Function unknown

J domain-containing protein	A0A5G2QJP3	0.268394	8	22	0.006396	Small molecule binding
Uncharacterized protein	A0A4X1T6P1	0.267592	1	3	0.003698	Positive regulation of cellular process;
Caveolin	A0A4X1VYQ8	0.266615	3	37	0.000957	Function unknown
Smad nuclear-interacting protein 1	I3LQU1	0.266605	6	19	0.006496	Cellular response to stress; ferric iron binding
Vitamin-K-epoxide reductase (warfarin-sensitive)	A0A5K1VIT7	0.266443	4	22	0.01449	Cellular localization;
Uncharacterized protein	I3LD98	0.266102	5	14	0.01958	Function unknown
ERA-like protein 1	A0A4X1TUP5	0.264921	1	21	0.01507	Function unknown
Gamma-tubulin complex component	A0A480PH59	0.264032	4	4	0.04159	Positive regulation of cellular process;
RNA helicase	A0A1D8MMA5	0.263411	50	67	0.002526	Function unknown
**Downregulated proteins in PK-15-infected cells (a total of 342 proteins)**						
L-lactate dehydrogenase	A0A0K0KW08	-1.65569	1	4	0.008237	Potassium ion import across plasma membrane; oxidoreductase activity
FXYD domain-containing ion transport regulator	Q58K79	-1.41767	2	42	0.000648	Nucleoside phosphate metabolic process; sodium channel regulator activity
Uncharacterized protein	A0A4X1VD17	-1.058658	17	44	0.000516	Fatty-acyl-coa synthase activity
Fructose-bisphosphate aldolase	A0A287B8F3	-1.025747	3	68	0.005583	Terpenoid biosynthetic process;
Cellular retinol binding protein 1	Q53J08	-1.002595	2	57	0.004152	Binding
Sickle tail protein homolog isoform 1	A0A480S813	-0.959734	3	28	0.001971	Function unknown
Retinol-binding protein 2	F1SL53	-0.937778	9	67	0.000146	Retinal binding
Uncharacterized protein	A0A5G2R3Z4	-0.935147	30	53	0.001086	Function unknown
Kinesin light chain	A0A5G2QYG0	-0.879443	1	7	0.004041	Function unknown

Tropomyosin alpha-1 chain	A0A4X1TT60	-0.845727	1	48	0.002443	Function unknown
Uncharacterized protein	A0A5G2R1N7	-0.827496	1	1	0.002978	Positive regulation of cellular process;
Amphiregulin	Q9BDH4	-0.821443	2	11	0.006965	Positive regulation of cellular process; growth factor receptor binding
Uncharacterized protein	A0A4X1UFQ6	-0.815827	18	58	0.000685	Integrin binding
Acyl-CoA-binding protein	A0A4X1SLV1	-0.773105	2	33	0.000278	Function unknown
Low-density lipoprotein receptor-related protein 2	C0HL13	-0.756897	2	24	0.01257	Cation transmembrane transport;
Cytochrome c oxidase subunit 1	A0A097P5T5	-0.737993	2	7	0.000336	Regulation of cellular process; transmembrane transporter activity
Costars domain-containing protein	A0A4X1VHY8	-0.730803	2	47	0.008894	Function unknown
Hypoxanthine phosphoribosyltransferase	A0A4X1WAA9	-0.72164	1	49	0.04817	Cellular metabolic process;
Diamine acetyltransferase 2	Q7PCJ9	-0.707385	7	38	0.000591	Binding
Alkaline phosphatase	A0A287BSC3	-0.707286	3	4	0.002059	Function unknown
Protein cordon-bleu isoform b	A0A480RTP6	-0.705338	7	12	0.003087	Negative regulation of cellular process;
SERPIN domain-containing protein	A0A5K1UE53	-0.677183	1	1	0.001041	Positive regulation of cellular process; serine-type endopeptidase inhibitor activity
Insulin-like growth factor-binding protein 2	P24853	-0.676014	11	47	0.004905	Regulation of endothelial cell proliferation; protein binding
Uncharacterized protein	A0A4X1VRC0	-0.670443	4	32	0.01036	System process; heparin binding
Caldesmon isoform X1	A0A480TAI8	-0.670386	19	40	0.001695	Calmodulin binding
Uncharacterized protein	I3L9K5	-0.654332	5	29	0.0012	Function unknown
1,2-dihydroxy-3-keto-5-methylthiopentene dioxygenase (Fragment)	A0A481CLJ7	-0.638811	1	32	0.00106	Positive regulation of cellular process;

Phosphoenolpyruvate carboxykinase, cytosolic [GTP]	A0A4X1UM84	-0.632784	12	32	0.007105	Small molecule metabolic process; small molecule binding
L-lactate dehydrogenase	A0A480X8T8	-0.628783	17	55	0.000186	Oxidoreductase activity
Na(+)/H(+) exchange regulatory cofactor NHE-RF	B6EAV5	-0.627117	1	56	0.000711	Negative regulation of blood coagulation; binding, bridging
Vitamin K-dependent protein S	A0A5G2R920	-0.618917	15	30	0.001772	Ruffle assembly; metal ion binding
Uncharacterized protein	A0A4X1TC08	-0.616733	2	43	0.0178	Function unknown
Uncharacterized protein	A0A287APU9	-0.59527	3	5	0.000101	Function unknown
Uncharacterized protein	A0A4X1W2Y2	-0.594373	15	32	0.01214	Regulation of multicellular organismal development;
BZIP domain-containing protein	A0A4X1VLW8	-0.589067	2	23	0.00119	Double-stranded DNA binding
Uncharacterized protein	A0A287AXR4	-0.580111	2	9	0.001667	Function unknown
Uncharacterized protein	A0A5G2QST4	-0.579922	1	27	0.001039	Function unknown
Glutathione transferase	Q29057	-0.576697	1	59	0.002137	Catalytic activity
Triosephosphate isomerase	A0A288CFT0	-0.573473	19	77	0.000588	Function unknown
Fumarylacetoacetase	A0A4X1UNC5	-0.567782	11	29	0.000886	Positive regulation of cellular process;
RanBP2-type domain-containing protein	A0A287AD58	-0.562028	1	4	0.01838	Transcription regulator activity
Ankyrin-3 isoform X12	A0A480UC11	-0.551487	15	14	0.00081	Function unknown
Uncharacterized protein	A0A4X1U9G6	-0.550245	3	5	0.03181	Function unknown
Quinone oxidoreductase	F6Q2A5	-0.549311	7	23	0.003908	Function unknown
Lipocln_cytosolic_FA-bd_dom domain-containing protein	A0A4X1V211	-0.544069	1	7	0.0046	Negative regulation of cellular process; retinal binding
Ketimine reductase mu-crystallin	F1RPC8	-0.542448	5	26	0.001233	Chemical homeostasis; oxidoreductase activity, acting on the CH-NH2 group of donors, NAD or NADP as acceptor
Secretagogin	Q06A97	-0.535653	10	46	0.001982	Binding

Uncharacterized protein	A0A287A4W1	-0.53213	1	9	0.02859	Positive regulation of cellular process;
Protein S100	A0A5G2QR31	-0.53104	2	23	0.0385	Protein dimerization activity
1,2-dihydroxy-3-keto-5-methylthiopentene dioxygenase	A0A4X1UJZ2	-0.526069	1	54	0.000985	Cation transmembrane transport;
ATP synthase subunit a	Q1HBH9	-0.523885	2	10	0.00665	Organophosphate metabolic process; cation transmembrane transporter activity
Creatine kinase	A0A4X1TV43	-0.518675	15	60	0.000989	Regulation of nucleotide metabolic process; binding
Phosphoglycerate mutase	A0A287AJQ2	-0.517577	11	69	0.000793	Small molecule metabolic process; protein binding
Ribokinase	A0A5G2RG09	-0.515072	5	20	0.000592	Ribonucleotide binding
Perilipin	K4P7U5	-0.510151	9	23	0.000418	Small molecule metabolic process;
Isocitrate dehydrogenase [NADP]	A0A287A8M1	-0.505866	26	69	0.000683	Positive regulation of cellular process; isocitrate dehydrogenase (NADP+
Osteopontin	P14287	-0.505452	10	40	0.0364	Phosphorus metabolic process; integrin binding
Glucose-6-phosphate isomerase	P08059	-0.505432	24	51	0.001042	Organic substance metabolic process; small molecule binding
Glutathione transferase	F1RL37	-0.504809	4	16	0.01433	Small molecule metabolic process;
Malate dehydrogenase, cytoplasmic	A0A5G2QP21	-0.499727	1	63	0.01003	Positive regulation of cellular process; organic cyclic compound binding
Dipeptidyl peptidase 4	A0A5G2Q7G7	-0.497271	17	20	0.005219	Catalytic activity, acting on a protein
Adenylate kinase isoenzyme 1	A0A286ZQ79	-0.496369	10	41	0.000282	Function unknown
5-oxoprolinase isoform X1	A0A480MWT6	-0.495855	1	19	0.01018	Positive regulation of cellular process;
Phosphoglycerate kinase	F1RPH0	-0.492595	30	78	0.000998	Negative regulation of cellular process; small molecule binding
Uncharacterized protein	A0A4X1TTH9	-0.491744	12	82	0.000566	Function unknown
MFS domain-containing protein	A0A4X1VJW9	-0.49092	1	2	0.03072	Function unknown
Uncharacterized protein	A0A4X1W0P5	-0.489548	16	9	0.000956	Small molecule metabolic process;

Malate dehydrogenase	A0A5G2QFC2	-0.487374	4	42	0.001696	Cytoskeleton organization; organic cyclic compound binding
Uncharacterized protein	I3LQD0	-0.487216	5	21	0.00619	Binding
Uncharacterized protein	A0A5G2QCC6	-0.485885	7	49	0.00041	Function unknown
Uncharacterized protein	A0A5G2QMA5	-0.484465	1	20	0.008195	Negative regulation of cellular process;
Chloride intracellular channel protein	A0A287ADH9	-0.479114	15	66	0.000353	Transmembrane transporter activity
Protein LBH	A0A481B2K6	-0.478698	2	34	0.01035	Unsaturated fatty acid metabolic process;
15-oxoprostaglandin 13-reductase	A0A4X1TM83	-0.477052	9	36	0.000669	15-oxoprostaglandin 13-oxidase activity
Alpha-aminoadipic semialdehyde dehydrogenase	A0A481ARR7	-0.472792	25	57	0.000896	Function unknown
Uncharacterized protein	A0A4X1ULB5	-0.465157	1	1	0.02613	Negative regulation of cellular process;
Uncharacterized protein	A0A4X1THY8	-0.464468	2	8	0.006306	Metabolic process; glutathione binding
PKS_ER domain-containing protein	A0A4X1U010	-0.461184	1	5	0.003119	Protein transport; protein dimerization activity
t-SNARE coiled-coil homology domain-containing protein	A0A4X1VQQ8	-0.460293	2	20	0.01615	SNARE binding
Na(+)/H(+) exchange regulatory cofactor NHE-RF	A0A481BZK7	-0.459909	1	56	0.02682	Positive regulation of cellular process; binding, bridging
Villin-1	A0A480TKM7	-0.45918	30	39	0.000144	ATP generation from ADP; peptidase regulator activity
Fructose-bisphosphate aldolase	A0A4X1U602	-0.458422	1	69	0.006081	Positive regulation of cellular process; fructose-bisphosphate aldolase activity
CCAAT/enhancer-binding protein	E5Q8C6	-0.457309	5	15	0.01147	Transcription factor activity, sequence-specific DNA binding
Coiled-coil domain-containing protein 72	F2Z5V1	-0.456174	2	30	0.02078	Function unknown

VWFA domain-containing protein	A0A5G2RG33	-0.454652	4	3	0.0342	Function unknown
Non-specific lipid-transfer protein	A0A480SKS9	-0.454459	2	19	7.40E-05	Function unknown
LID domain-containing protein	A0A4X1TXN0	-0.451471	3	10	0.002021	Signal transduction;
Protein kinase C delta type	I3LR85	-0.448885	1	39	0.01016	Positive regulation of cellular process; ribonucleotide binding
COP9 signalosome complex subunit 9	I3LEN0	-0.446648	1	35	0.02383	Function unknown
UDP-sugar diphosphatase (Fragment)	Q865Y9	-0.444618	4	30	0.001242	Positive regulation of cellular process;
2-iminobutanoate/2-iminopropanoate deaminase	A0A286ZNH3	-0.442288	9	93	0.003565	MRNA binding
Cytochrome c oxidase subunit 7C, mitochondrial	Q1W0Y2	-0.436684	2	29	0.01166	Metabolic process; hydrogen ion transmembrane transporter activity
3-hydroxyacyl-[acyl-carrier-protein] dehydratase	A0A4X1U8L3	-0.435149	3	64	0.01657	Terpenoid biosynthetic process; binding
Farnesyl pyrophosphate synthase isoform a	A0A481DNJ7	-0.434844	1	25	0.003303	Geranyltranstransferase activity
VPS10 domain-containing protein	A0A287A1U0	-0.433849	11	13	0.01286	Response to steroid hormone;
3 beta-hydroxysteroid dehydrogenase/Delta 5-->4-isomerase	Q9N119	-0.43365	3	10	0.005512	Steroid delta-isomerase activity
Uncharacterized protein	A0A4X1TPF2	-0.432141	6	14	0.001942	Positive regulation of cellular process;
F-actin-uncapping protein LRRC16A isoform 1	A0A480L9D2	-0.431623	1	27	0.000468	Function unknown

Ribonucleoside-diphosphate reductase	A0A5G2QL80	-0.431519	2	59	0.007466	Cellular process;
Glycine amidinotransferase, mitochondrial	P50441	-0.431421	6	22	0.01179	Microtubule cytoskeleton organization; glycine amidinotransferase activity
Tubulin alpha chain	A0A480NAY0	-0.430773	3	57	0.001763	Ribonucleotide binding
Uncharacterized protein	A0A5G2QGQ5	-0.429475	1	41	0.02591	Small molecule metabolic process;
Adenosine kinase	A0A287ASQ2	-0.429458	20	70	0.000279	Positive regulation of cellular process; adenosine kinase activity
Activated RNA polymerase II transcriptional coactivator p15	A0A287AUW4	-0.427913	8	61	6.40E-05	Regulation of biological process; catalytic activity, acting on DNA
Insulin-like growth factor-binding protein 7 isoform 1	A0A481CLV1	-0.42724	10	51	0.01341	Insulin-like growth factor binding
Uncharacterized protein	A0A4X1UJK2	-0.427218	12	25	0.01799	Function unknown
Uncharacterized protein	A0A287B8I3	-0.426349	5	18	0.01353	Function unknown
Potassium channel tetramerization domain containing 12	F1RHE6	-0.42501	4	16	0.001075	Function unknown
Leydig cell tumor 10 kDa protein homolog	F1SD77	-0.423463	7	47	0.000233	Function unknown
Protein S100-A13	K9J4R8	-0.42314	5	42	0.001127	Signal transduction;
Protein kinase C	A0A287BBY6	-0.422849	1	38	0.03132	Small molecule metabolic process; ribonucleotide binding
Inositol polyphosphate 1-phosphatase isoform X1	F1SN51	-0.422821	11	35	0.003251	Regulation of biological process; inositol phosphate phosphatase activity
Peroxiredoxin-5	A0A5G2R9D6	-0.421862	10	75	0.000893	Oxidoreductase activity
NADH-ubiquinone oxidoreductase chain 3	B5KN66	-0.42056	1	13	0.02684	Oxidoreductase activity
COesterase domain-	A0A4X1T6P2	-0.420305	1	63	0.007994	Positive regulation of cellular process;

containing protein						
Peroxiredoxin-6	Q9TSX9	-0.419427	17	83	0.001948	Peroxiredoxin activity
Uncharacterized protein	A0A5G2RCE3	-0.418171	46	27	0.001815	Inositol 1,4,5 trisphosphate binding
Protein phosphatase 1 regulatory subunit 14B	Q8MIK9	-0.417237	3	48	0.003429	Glucose metabolic process; protein serine/threonine phosphatase inhibitor activity
Aldose 1-epimerase	A0A480TMR5	-0.412892	9	32	0.001497	Binding
Uncharacterized protein	A0A5G2RKV1	-0.412651	1	20	0.03449	Function unknown
Methyltransf_11 domain-containing protein	A0A4X1W6X4	-0.41247	1	26	0.02942	Function unknown
Peptidylprolyl isomerase	I3LR51	-0.411175	11	43	0.002514	Isomerase activity
Uncharacterized protein	A0A4X1T842	-0.409731	25	58	0.002014	Function unknown
Uncharacterized protein	A0A287BNG4	-0.406349	6	17	0.00834	Peptidase activity
Uncharacterized protein	A0A287AG72	-0.405239	7	25	0.00111	Positive regulation of cellular process;
Tyrosine-protein phosphatase non-receptor type	A0A5G2QDD3	-0.404551	1	48	0.01652	Negative regulation of cellular process; insulin receptor binding
39S ribosomal protein L59, mitochondrial	A0A4X1T8J5	-0.403347	8	40	0.001932	Function unknown
Complex I-MNLL	F1SD73	-0.40302	2	34	0.001222	Function unknown
Uncharacterized protein	A0A4X1SSQ0	-0.402023	2	5	0.001051	Function unknown
Transmembrane 4 L6 family member 4	F1SJP1	-0.401571	1	8	0.003379	Function unknown
15-oxoprostaglandin 13-reductase	A0A287B7R3	-0.401287	15	57	0.001302	Function unknown
GRAM domain-containing protein	A0A287BMM1	-0.401	2	3	0.04357	Function unknown
Non-specific lipid-transfer	D0G7F1	-0.400355	2	19	0.001096	Function unknown

protein						
Acylphosphatase	J9JIK5	-0.399343	2	12	0.024	Acylphosphatase activity
Uncharacterized protein	A0A5G2QLE5	-0.399108	7	9	0.00495	Leukotriene biosynthetic process;
Microsomal glutathione S-transferase 3	Q2EN77	-0.398983	3	25	0.01127	Cellular biosynthetic process; glutathione peroxidase activity
Spermine synthase isoform X1	K7GPY8	-0.398757	21	74	0.000671	Regulation of mRNA metabolic process; catalytic activity
CSD_1 domain-containing protein	A0A4X1VPW4	-0.398064	5	62	0.004481	Negative regulation of cellular process; mRNA binding
ATP-dependent dihydroxyacetone kinase	A0A480YP74	-0.397405	2	52	0.000575	Small molecule binding
Asparaginyl endopeptidase	F1SD70	-0.396942	11	38	0.001487	Catalytic activity
Nicotinate phosphoribosyltransferase	A0A480KYX1	-0.395898	16	43	0.002441	Positive regulation of cellular process;
14-3-3 protein theta	A0A4X1UGF0	-0.393481	9	54	0.000136	Small molecule metabolic process; protein binding
Asparaginase-like protein 1	A0A4X1VNE4	-0.392228	11	42	0.00258	Regulation of viral genome replication; hydrolase activity, acting on carbon-nitrogen (but not peptide
Peptidyl-prolyl cis-trans isomerase A	P62936	-0.392093	1	76	0.001367	Binding
Protein archease	A0A4X1VY44	-0.391691	2	16	0.000305	Function unknown
Beta-lactamase-like protein 2	F1RU12	-0.3887	14	59	0.001267	Reproductive process; zinc ion binding
AMP-binding domain-containing protein	A0A4X1W4H3	-0.387772	27	66	0.003053	COA-ligase activity
Uncharacterized protein	F1SH42	-0.38733	6	19	0.02766	Function unknown
DNA helicase	A0A480HA17	-0.386132	3	8	0.04134	Function unknown
Uncharacterized protein	F1SES5	-0.38322	14	28	0.000196	Function unknown
Delta-aminolevulinic acid	A0A481BAE5	-0.382531	10	39	7.20E-05	ATP generation from ADP;

dehydratase						
Phosphoglycerate mutase	A0A286ZQ31	-0.382129	9	38	0.0329	Cellular metabolic process; isomerase activity
NADH-ubiquinone oxidoreductase chain 5	T1P3V6	-0.381783	6	12	0.008396	Oxidoreductase activity, acting on NAD(P
Carboxymethylenebutenolidase homolog	A0A480VUT1	-0.380726	2	40	0.000527	Positive regulation of cellular process;
USP1-associated factor 1	A0A4X1TUZ9	-0.380324	13	28	0.03082	Protein binding
Uncharacterized protein	A0A4X1UXT4	-0.379015	2	20	0.001309	Oxidoreductase activity
Urokinase plasminogen activator surface receptor	A0A0B8RSD8	-0.378512	4	16	0.0353	Cation transmembrane transport;
ATP synthase protein 8	Q71K49	-0.378512	2	39	0.0363	Transmembrane transporter activity
Nucleoside diphosphate-linked moiety X motif 19	A0A4X1TSV9	-0.378463	1	39	0.00018	Function unknown
Uncharacterized protein	A0A4X1U8I9	-0.378433	8	22	0.01004	Small molecule metabolic process;
Aminoacylase-1	P37111	-0.377821	17	63	0.002884	Mitochondrial electron transport, NADH to ubiquinone; peptidase activity, acting on L-amino acid peptides
NADH dehydrogenase [ubiquinone] 1 beta subcomplex subunit 8, mitochondrial	A0A480QR88	-0.377331	7	53	0.006076	Negative regulation of cellular process; oxidoreductase activity, acting on NAD(P
LIM domain and actin-binding protein 1	A0A5G2QDK2	-0.376528	3	49	0.01122	Function unknown
Beta-1 metal-binding globulin	A0A5G2R5N9	-0.376009	6	11	0.002634	Peptide metabolic process;
GST class-pi	M3V836	-0.375516	8	75	0.001505	Catalytic activity
Aldehyde dehydrogenase	I6L6E1	-0.374492	1	21	0.004509	Regulation of cellular component organization;
Dihydropyrimidinase-related	G9F6X9	-0.373856	23	69	0.001038	Cytoskeletal protein binding

protein 2						
Uncharacterized protein	F1RSL4	-0.370593	9	7	0.001693	Small molecule metabolic process;
Acyl-coenzyme A oxidase	A0A4X1VH96	-0.370109	11	22	0.003602	Acyl-COA oxidase activity
Coronin	A0A5G2R8I5	-0.370078	19	37	0.00094	Function unknown
Uncharacterized protein	I3LJU0	-0.369657	5	7	0.02259	Cellular metabolic process;
D-3-phosphoglycerate dehydrogenase	A0A480HK41	-0.369523	22	48	0.00065	Glycosyl compound biosynthetic process; binding
Hypoxanthine-guanine phosphoribosyltransferase	Q45FY6	-0.369405	2	67	0.000323	Positive regulation of cellular process; small molecule binding
Nucleoside diphosphate kinase (Fragment)	A0A481D0P1	-0.366904	7	60	0.02676	G-quadruplex DNA binding
Uncharacterized protein	A0A287BL36	-0.366077	2	15	0.04863	Function unknown
Na(+)/H(+) exchange regulatory cofactor NHE-RF	F6PYE5	-0.365401	18	56	0.00281	Function unknown
Aldehyde oxidase 1	A0A287A5W9	-0.365293	8	9	0.003035	Function unknown
Heme-binding protein 1	A0A286ZSH3	-0.364906	12	67	0.000734	Heterocyclic compound binding
SMB domain-containing protein	I3L9E7	-0.364027	6	17	0.04134	Function unknown
ADP-ribosyl cyclase/cyclic ADP-ribose hydrolase	A0A4X1VFI7	-0.361585	7	27	0.003481	Phosphorus metabolic process;
UMP-CMP kinase	Q29561	-0.360476	9	54	5.90E-05	Negative regulation of vascular permeability; anion binding
Uncharacterized protein	F1SNE5	-0.360176	7	38	0.008498	Positive regulation of cellular process; protein binding
Moesin	A0A4X1VJV5	-0.357329	28	58	0.008235	Receptor binding
Doublecortin domain-containing protein 2	A0A480EUM6	-0.356438	2	28	0.001429	Function unknown
Carbonyl reductase 3	A0A0K1TQQ7	-0.356221	15	70	0.002014	Oxidoreductase activity

PDZ domain-containing protein GIPC2 isoform 1	A0A287BNZ1	-0.354778	12	40	0.01202	Binding
Cytochrome P450	A7KZR2	-0.354691	2	46	0.01894	Regulation of biological process; oxidoreductase activity
GTP cyclohydrolase 1 feedback regulatory protein	A0A4X1UVQ7	-0.353935	2	50	0.001424	Function unknown
Uncharacterized protein	A0A286ZRK0	-0.353695	1	48	0.004348	Function unknown
N-myc downstream-regulated gene 1 protein	A0A4X1SPG7	-0.352586	12	53	0.005601	Regulation of cellular protein metabolic process;
M20_dimer domain-containing protein	F1S0E1	-0.351764	3	9	0.01083	Peptidase activity, acting on L-amino acid peptides
FERM domain-containing protein 4B	A0A287B6L4	-0.351271	1	1	0.02184	Positive regulation of cellular process;
Stathmin	Q6DUB7	-0.350542	8	46	0.004706	Positive regulation of cellular process; protein binding
Uncharacterized protein	I3LB77	-0.350338	5	17	0.02929	MRNA 3'-UTR binding
Intraflagellar transport protein 27 homolog	A0A480Z612	-0.34918	9	59	0.002595	Function unknown
Hepatoma-derived growth factor-related protein 2	A0A0B8RTM7	-0.34882	13	19	0.004738	Lipid droplet formation;
Seipin	A0A4X1VK87	-0.348695	1	5	0.0481	Lipid binding
Phosphodiesterase	A0A481A4V1	-0.347988	7	16	0.04711	Function unknown
Uncharacterized protein	A0A286ZSE5	-0.347469	1	37	0.02082	Protein metabolic process;
Alpha-1-microglobulin	A0A480P2R0	-0.347466	2	7	0.01708	Regulation of biological process; protein dimerization activity
CARD domain-containing protein	A0A4X1UPG3	-0.347257	4	32	0.007917	Function unknown
Protein XRP2	A0A480E0X6	-0.34678	6	18	0.004732	Binding
Arginyl-tRNA--protein	A0A480M856	-0.346753	5	17	0.008626	Function unknown

transferase 1						
Hepatocyte nuclear factor 1-beta	A0A4X1T4P3	-0.345726	1	24	0.02009	Signal transduction;
Uncharacterized protein	A0A287B0I3	-0.345709	8	54	0.002706	Protein binding
Aldolase_II domain-containing protein	A0A4X1TEQ8	-0.345664	15	27	0.02353	Structural molecule activity
Epididymal-specific lipocalin-9 isoform X1	A0A287A022	-0.344294	1	5	0.03129	Negative regulation of cellular process; binding
Uveal autoantigen with coiled-coil domains and ankyrin repeats isoform X3	A0A481D4V5	-0.343429	1	9	0.02512	Positive regulation of cellular process;
Interferon regulatory factor	A0A5S6GDS9	-0.340442	5	24	0.006196	Unsaturated fatty acid metabolic process; transcription factor activity, sequence-specific DNA binding
Aldo-keto reductase	Q2TJA5	-0.340027	16	70	7.50E-05	Catalytic activity
Uncharacterized protein	I3LJJ4	-0.339319	14	60	0.00134	Function unknown
2,4-dienoyl-CoA reductase 1	D6QST6	-0.337855	12	44	0.001467	Positive regulation of cellular process;
Macrophage migration inhibitory factor	P80928	-0.335845	3	37	0.003433	Dopachrome isomerase activity
UDENN domain-containing protein	I3LCU0	-0.335461	2	2	0.006546	Function unknown
Transcription factor jun-D	A0A4X1UAA4	-0.335168	3	19	0.0142	Function unknown
Uncharacterized protein	A0A4X1UK48	-0.334823	10	19	0.02741	Translation; RNA binding
60S acidic ribosomal protein P1	A0A4X1T568	-0.333922	4	96	0.04705	Enzyme regulator activity
Protein-ribulosamine 3-kinase	A0A4X1U3C7	-0.333868	4	20	0.0446	Negative regulation of cellular process;
Tubulin beta chain	A0A480UV25	-0.331949	3	79	0.03349	Anion binding

CXXC motif containing zinc binding protein	F1S765	-0.330612	9	74	0.000435	Zinc ion binding
Phosphorylase b kinase regulatory subunit	A0A4X1TYG1	-0.330364	5	6	0.007787	Function unknown
Cystatin-B	A0A4X1TD05	-0.328578	3	24	0.01215	Negative regulation of cellular process;
Rab GDP dissociation inhibitor	I3L893	-0.328292	15	64	0.004224	Enzyme regulator activity
Rab9 effector protein with kelch motifs isoform X1	A0A480XCZ0	-0.327652	8	31	0.002356	Cellular metabolic process;
NEDD8	A0A4X1SPV9	-0.327639	3	32	0.00901	Positive regulation of cellular process; ubiquitin-like protein ligase binding
4a-hydroxytetrahydrobiopterin dehydratase	A0A4X1TCL6	-0.326302	3	37	0.01391	Phenylalanine 4-monooxygenase activity
Uncharacterized protein	I3LU51	-0.325978	1	37	0.01667	Function unknown
Methylthioribulose-1-phosphate dehydratase	A0A4X1SQ75	-0.32458	14	68	0.000778	Function unknown
Solute carrier family 37 (Glucose-6-phosphate transporter), member 4	K9IVK4	-0.323228	5	15	0.003125	Function unknown
mRNA decay activator protein ZFP36L2 (Fragment)	A0A480Z3B2	-0.323004	9	19	0.032	Ion binding
Uncharacterized protein	F1RGT4	-0.321767	3	43	0.02293	Function unknown
Ubiquitin carboxyl-terminal hydrolase	A0A287B8P7	-0.320536	3	6	0.002042	Function unknown
Myosin_tail_1 domain-containing protein	A0A287AXZ5	-0.319969	48	43	0.000903	Regulation of secretion by cell; motor activity
Uncharacterized protein	A0A287A0S1	-0.319833	7	33	0.00256	Phosphatidylinositol binding
Arpin	I3LLA5	-0.319553	5	32	0.004924	Regulation of nitrogen compound metabolic process;

ATP-binding cassette sub-family F member 1 isoform a	A0A480PQG3	-0.319493	2	46	0.01028	Ribonucleotide binding
Cytochrome c oxidase subunit 6C	A0A4X1TXH1	-0.31939	5	53	0.000105	Oxidoreductase activity
Microtubule-actin cross-linking factor 1 isoform X9	A0A480X1F7	-0.319279	1	43	0.007265	Function unknown
Uncharacterized protein	A0A4X1VVM2	-0.318648	4	49	0.03181	Function unknown
Protein-serine O-palmitoleoyltransferase porcupine isoform A	A0A480H9U3	-0.318461	1	2	0.01995	Function unknown
Tetratricopeptide repeat protein 38	A0A4X1W3T6	-0.318274	7	22	0.01275	Function unknown
Sugar transporter SWEET	A0A4X1W094	-0.31813	1	4	0.02112	Signal transduction;
LIM zinc-binding domain-containing protein	A0A4X1TWY1	-0.317759	3	31	0.001044	Heterocyclic compound binding
Uncharacterized protein	I3LLQ7	-0.317397	2	15	0.006309	Function unknown
LRRcap domain-containing protein	A0A4X1T7J4	-0.316314	10	36	0.000586	Function unknown
PKD domain-containing protein	A0A286ZJF6	-0.316025	7	13	0.0233	Function unknown
39S rib	A0A4X1UFR4	-0.314965	2	21	0.01324	Function unknown
Dimethylargininase	A0A287B1T7	-0.314855	9	39	0.02	Function unknown
Uncharacterized protein	A0A4X1UEJ0	-0.313322	1	36	0.001966	MRNA processing;
Zinc finger Ran-binding domain-containing protein 2	A0A5S6FXB9	-0.312628	11	34	0.000698	RNA binding
Histone deacetylase	A0A287BQF9	-0.31203	2	2	0.01073	Protein catabolic process;

Lys	A0A287B836	-0.311839	1	10	0.01384	Cellular modified amino acid metabolic process; protein binding
Glutathione transferase	A0A4X1TGN8	-0.311547	3	27	0.03108	Function unknown
3-ketoacyl-CoA thiolase, mitochondrial	D0G0B3	-0.311267	17	71	0.000508	Anatomical structure development;
Uncharacterized protein	A0A4X1V9E1	-0.31075	24	59	0.002265	Macromolecule metabolic process; macromolecular complex binding
Proteasome subunit beta type-7	A1XQU1	-0.309783	7	39	0.009788	Intermediate filament-based process; peptidase activity, acting on L-amino acid peptides
Dystonin isoform X21	A0A480SGQ6	-0.308739	2	31	0.02895	Nucleoside phosphate metabolic process; protein binding
Propionate--CoA ligase	A0A480Y097	-0.308192	7	12	0.000728	Ligase activity
Stearoyl-CoA 9-desaturase	F6Q284	-0.307807	9	24	0.03895	Function unknown
UBC core domain-containing protein	A0A5G2RGI4	-0.307711	4	58	0.001254	Function unknown
Superoxide dismutase [Cu-Zn]	I3LUD1	-0.307703	5	29	0.01039	Carboxylic acid catabolic process; oxidoreductase activity
Alcohol dehydrogenase NADP(+)	A0A480PFL9	-0.307536	20	73	0.001441	Positive regulation of cellular process; allyl-alcohol dehydrogenase activity
Cystathionine gamma-lyase	F1S9N9	-0.307278	17	52	0.002712	Negative regulation of cellular process; small molecule binding
PWWP domain-containing protein	A0A4X1UJT9	-0.30443	5	28	0.004086	Protein binding
Uncharacterized protein	A0A287B3Q3	-0.303128	2	1	0.006746	Function unknown
Cytochrome c oxidase subunit	A0A4X1TG97	-0.302959	7	73	0.006029	Function unknown
Uncharacterized protein	F1SR85	-0.302472	17	30	0.002697	Response to organic substance;
Uncharacterized protein	I3LNG8	-0.301398	1	65	0.04209	Binding
AdoHcyase_NAD domain-containing protein	A0A286ZSJ9	-0.30099	2	26	0.001114	Signal transduction;
Alpha-carbonic anhydrase domain-containing protein	A0A4X1SLG9	-0.30092	7	35	0.007198	Positive regulation of cellular process; hydro-lyase activity

Serine/threonine-protein ph	P67776	-0.29966	1	54	0.000352	Protein phosphatase 2A binding
Uncharacterized protein	A0A287A3A3	-0.299088	11	26	0.01543	Function unknown
14-3-3 protein beta/alpha (Fragment)	A0A480LG53	-0.298991	6	43	0.000558	Insulin-like growth factor binding
NAD(P)H dehydrogenase quinone 1	F1S395	-0.298987	15	43	0.000379	Positive regulation of cellular process; oxidoreductase activity
ENTH domain-containing protein	A0A4X1U6J4	-0.297517	1	43	0.03508	Low-density lipoprotein particle receptor binding
Uncharacterized protein	A0A4X1SD92	-0.296612	7	26	0.003171	Signal transduction;
Alpha-actinin-4 isoform 1	A0A480HSJ4	-0.295963	3	68	0.02896	Protein binding
Protein HID1 isoform X1	A0A286ZYE1	-0.295357	14	25	0.001474	Function unknown
Uncharacterized protein	A0A4X1UXN6	-0.294897	14	10	0.006181	Function unknown
Uncharacterized protein	A0A287AIZ9	-0.294619	12	21	0.01448	Protein transport;
ADP-rib	F1SPB1	-0.29382	2	6	0.002943	Enzyme activator activity
Anaphase-promoting complex subunit 4	I3LFB6	-0.293291	3	5	0.01304	Function unknown
Methyltransf_11 domain-containing protein	A0A287ATV3	-0.292708	4	15	0.000334	Function unknown
Uncharacterized protein	I3LV99	-0.292255	63	40	0.000458	Function unknown
Retinal dehydrogenase 1	I3LRS5	-0.291963	29	75	0.001811	Regulation of biological process;
Peroxiredoxin-2	A0A287AJ76	-0.290557	10	59	0.002314	Oxidoreductase activity
Beta-hex	A0A4X1SN58	-0.290186	14	28	0.009624	Function unknown
Uncharacterized protein	A0A4X1W0G3	-0.290106	5	44	0.01259	Regulation of biological process;
Rho guanine nucleotide exchange factor 18 isoform X6	A0A286ZWT3	-0.289858	7	11	0.01262	Binding
Uncharacterized protein	A0A286ZMY1	-0.288919	6	6	0.01225	Function unknown

COMM domain-containing protein 1	A0A4X1WCT2	-0.288754	3	27	0.003326	Function unknown
Uncharacterized protein	A0A4X1UHE2	-0.288496	9	36	0.001105	Function unknown
Procollagen-lysine 5-dioxygenase	F1SKE2	-0.28793	33	43	0.000387	Function unknown
Ensconsin isoform 3	A0A480SUG5	-0.286855	12	19	0.006653	Covalent chromatin modification;
Protein dpy-30 homolog isoform 1	F2Z5F7	-0.286744	4	47	0.006445	Protein dimerization activity
UBC core domain-containing protein	A0A5G2RDX3	-0.286304	7	65	0.000984	Binding
39S rib	A0A5G2QIA8	-0.286129	8	47	0.009369	Function unknown
Transaldolase	A0A287BQ72	-0.285256	12	30	0.01058	Function unknown
Glyceraldehyde-3-ph	A0A4X1TLU8	-0.284229	1	12	0.0124	Negative regulation of cellular process;
14-3-3 protein zeta/delta	A0A480PLY3	-0.283971	10	57	0.000104	Cadherin binding
Gamma-glutamylcyclotransferase	A0A4X1T868	-0.283782	9	49	0.004633	Gamma-glutamylcyclotransferase activity
RELT-like protein 1	F1S4K9	-0.283123	5	30	0.001757	DNA metabolic process;
S	A0A480MDH4	-0.282382	2	23	0.01873	Phosphorus metabolic process;
Bifunctional UDP-N-acetylgluc	A0A480TMZ9	-0.282209	14	23	0.000913	Phosphotransferase activity, alcohol group as acceptor
2-ph	A0A480R622	-0.282087	23	69	0.00122	MRNA processing;
AdoHcyase_NAD domain-containing protein	A0A5G2QQD1	-0.281702	10	42	0.000297	Positive regulation of cellular process; protein binding
Myotrophin	K9IVW4	-0.280889	6	57	0.003858	Oxidation-reduction process;
NADH dehydrogenase [ubiquinone] iron-sulfur protein 4, mitochondrial	A0A5G2QZZ2	-0.28049	3	22	0.0388	Oxidoreductase activity

Armadillo repeat-containing protein 10 isoform b	I3LLX9	-0.280353	8	47	0.0142	Peptide metabolic process;
Glutathione S-transferase kappa	F1SRV4	-0.280317	10	50	0.000738	Disulfide oxidoreductase activity
Oxysterol-binding protein	A0A4X1UAF6	-0.27994	6	13	0.005616	Positive regulation of cellular component biogenesis;
Uncharacterized protein	A0A4X1V5D3	-0.279128	1	34	0.0145	GTPase regulator activity
1,4-alpha-glucan branching enzyme	A0A480PP98	-0.278886	15	29	0.008577	Sulfur compound metabolic process;
Rhodanese domain-containing protein	A0A4X1VRY9	-0.278842	7	83	5.00E-05	Primary metabolic process; catalytic activity
N-acetylneuraminic acid synthase	D0G780	-0.2787	14	52	0.005312	Regulation of biological process;
Peroxiredoxin-1	A0A286ZND5	-0.278199	10	64	0.000405	Chromosome organization; oxidoreductase activity
Cohesin loading complex subunit SCC4 homolog	A0A4X1U555	-0.277883	4	12	0.000566	Positive regulation of cellular process; double-stranded DNA binding
Mitochondrial fission 1 protein	A0A287ADK7	-0.277162	6	34	0.005345	Positive regulation of cellular process;
Dynein light chain Tctex-type 1	B8Y650	-0.276979	2	30	8.70E-05	Nucleoside-triphosphatase activity
IFT81_CH domain-containing protein	A0A5G2QXF1	-0.276575	5	7	0.02051	Function unknown
Kelch-like protein 14	F1SAK5	-0.276079	4	8	0.00103	Function unknown
Erythrocyte membrane protein band 4.1 like 4A	I3LA05	-0.276033	2	4	0.02506	Function unknown
Aminomethyltransferase	A0A5G2RKE1	-0.275887	5	16	0.02581	Protein catabolic process;
Cathepsin D	P00795	-0.275814	1	38	0.02072	Aspartic-type endopeptidase activity
Immortalization up-regulated protein	A0A480XPD4	-0.275789	2	12	0.04449	Function unknown

5'-deoxynucleotidase HDDC2	F1S2V3	-0.275682	9	61	0.000612	Methylation; 5'-deoxynucleotidase activity
Thiopurine S-methyltransferase	A0A481DB59	-0.275546	2	74	0.00314	Negative regulation of cellular process; methyltransferase activity
Ph	A0A480TQY6	-0.274743	10	12	0.000433	SH3 domain binding
PWWP domain-containing protein	A0A4X1VZD7	-0.274181	16	61	0.00033	Function unknown
COMM domain-containing protein	A0A286ZXE0	-0.274044	4	47	0.01195	Function unknown
Tropomy	A0A287B5N7	-0.27358	9	63	0.006347	Regulation of translation;
Citrate hydro-lyase	A0A4X1UMC0	-0.273565	36	51	0.001354	Metal cluster binding
6-ph	A0A480YSA5	-0.273101	22	56	0.000334	Function unknown
LisH domain-containing protein ARMC9	A0A480YVZ9	-0.272983	2	3	0.006966	Function unknown
Urokinase-type plasminogen activator	A0A286ZXK2	-0.272819	9	24	0.03093	Positive regulation of cellular process;
AP-1 complex subunit gamma	A0A5K1VTT2	-0.272774	28	43	0.002358	Decidualization; protein binding
Cathepsin B	B2CNZ7	-0.272538	10	41	0.01872	Catalytic activity, acting on a protein
PH domain-containing protein	A0A5G2R3Q3	-0.271426	2	4	0.02066	Protein metabolic process;
Glutaredoxin domain-containing protein	A0A4X1SD79	-0.271117	2	26	0.006196	Disulfide oxidoreductase activity
F-BAR domain only protein 2	A0A5G2R436	-0.269438	6	10	0.001239	Reproductive process;
Acyl-CoA synthetase long-chain family member 4 (Fragment)	A6YQV5	-0.269122	1	38	0.01074	COA-ligase activity
